# Selective Nonenzymatic Formation of Biologically Common RNA Hairpins

**DOI:** 10.1002/anie.202417370

**Published:** 2024-11-26

**Authors:** Long‐Fei Wu, Junting Zhang, Robert Cornwell‐Arquitt, David A. Hendrix, Aleksandar Radakovic, Jack W. Szostak

**Affiliations:** ^1^ Howard Hughes Medical Institute The University of Chicago Chicago IL 60637 USA; ^2^ The University of Chicago Chicago IL 60637 USA; ^3^ Department of Biochemistry and Biophysics Oregon State University USA; ^4^ School of Electrical Engineering and Computer Science Oregon State University USA; ^5^ Current address: Frontiers Science Center for Transformative Molecules School of Chemistry and Chemical Engineering Shanghai Jiao Tong University Shanghai 200240 China

**Keywords:** origin of life, prebiotic chemistry, loop-closing ligation, RNA assembly, RNA structure

## Abstract

The prebiotic formation of RNA building blocks is well‐supported experimentally, yet the emergence of sequence‐ and structure‐specific RNA oligomers is generally attributed to biological selection via Darwinian evolution rather than prebiotic chemical selectivity. In this study, we used deep sequencing to investigate the partitioning of randomized RNA overhangs into ligated products by either splinted ligation or loop‐closing ligation. Comprehensive sequence‐reactivity profiles revealed that loop‐closing ligation preferentially yields hairpin structures with loop sequences UNNG, CNNG, and GNNA (where N represents A, C, G, or U) under competing conditions. In contrast, splinted ligation products tended to be GC rich. Notably, the overhang sequences that preferentially partition to loop‐closing ligation significantly overlap with the most common biological tetraloops, whereas the overhangs favoring splinted ligation exhibit an inverse correlation with biological tetraloops. Applying these sequence rules enables the high‐efficiency assembly of functional ribozymes from short RNAs without template inhibition. Our findings suggest that the RNA tetraloop structures that are common in biology may have been predisposed and prevalent in the prebiotic pool of RNAs, prior to the advent of Darwinian evolution. We suggest that the one‐step prebiotic chemical process of loop‐closing ligation could have favored the emergence of the first RNA functions.

## Graphical abstract

Template‐free RNA loop‐closing ligation selectively generates RNA tetraloops that are prevalent in biology, as demonstrated by high‐throughput sequencing. Utilizing high‐yield overhang sequences enables the efficient assembly of functional RNAs.

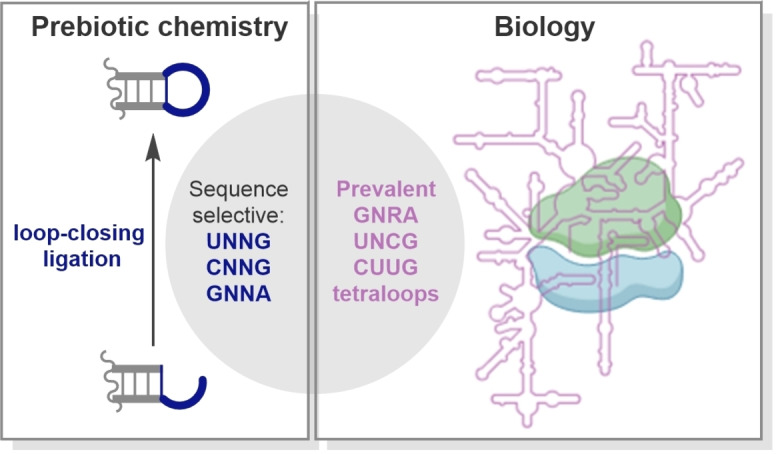

## Introduction

The mechanisms by which functional RNAs first emerged is a central topic of inquiry within the RNA world hypothesis of the origin of life. Like proteins, RNA functions depend on higher‐order structures that are dictated by linear sequences.[^1^, ^2^] Unlike proteins, RNA structures are characterized by stable and isolable secondary structures, which hierarchically connect the primary sequence to three‐dimensional structures.^[3]^ Typically, the energetic contributions of tertiary interactions between preformed RNA secondary structures are small compared to the greater stabilizing energy of base‐paired stems.[^1^, ^2^, ^3^] RNA hairpins are the most prevalent secondary structures^4^, and together with bulges and internal loops, they play crucial roles in biological RNAs. They function as nucleation sites during RNA folding and facilitate essential RNA‐RNA and RNA‐protein interactions.[^5^, ^6^] We propose that the initial selective formation of elementary secondary structures, such as hairpin stem‐loops, would have been crucial for the subsequent assembly of more complex structures and thus the emergence of functional RNAs.[^7^, ^8^, ^9^]

Two potentially prebiotic plausible processes, splint‐directed ligation and template‐free loop‐closing ligation have been employed in constructing full‐length functional RNAs from short oligonucleotides.[^10^, ^11^, ^12^] However, how these two nonenzymatic pathways could have constrained the sequence and structure of their products towards biological RNAs remains largely unknown.[^13^, ^14^] In an enzymatic template‐directed DNA ligation/replication model system, non‐folding sequences are enriched because the self‐folding property of hairpin structures suppresses their own amplification,^[15]^ in contrast to the prevalence of hairpins in functional RNAs. Similarly, both hairpins and their complements are known to block templated nonenzymatic copying chemistry,[^16^, ^17^] which typically requires unfolded templates. But common biological RNA tetraloops, such as UNCG (N representing A, C, G, or U) and GNRA (R representing A or G), fold rapidly and exhibit very high thermodynamic stability.[^18^, ^19^, ^20^, ^21^] This raises the question of how such functionally important RNA structures, with their stringent sequence constraints,^[22]^ could have been formed prior to the Darwinian evolution of highly active and processive RNA polymerase ribozymes.

**Figure 1:**
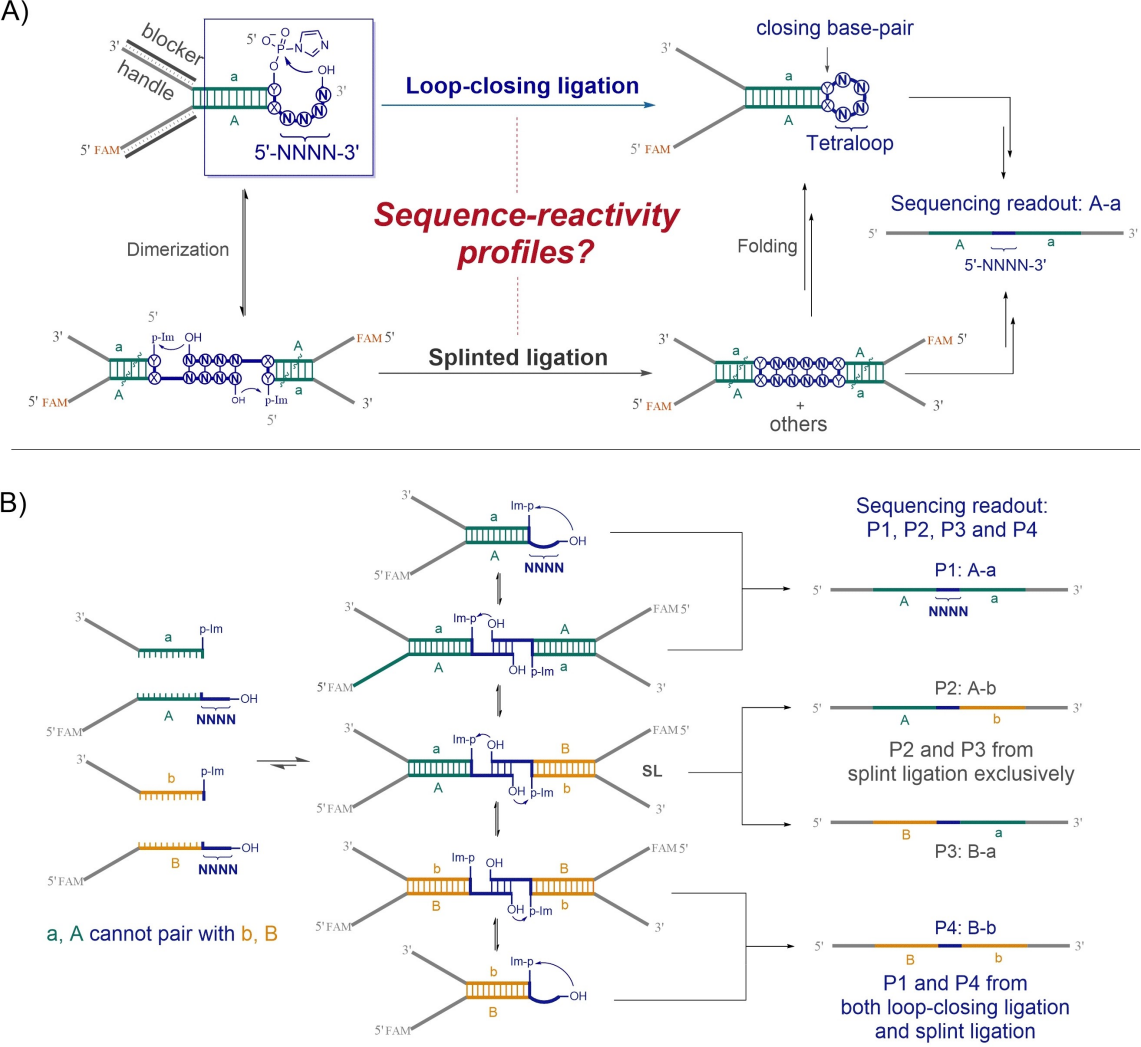
Determining the sequence‐reactivity profiles of RNA loop‐closing ligation. A) Schematic depiction of loop‐closing ligation and its competing nicked‐duplex ligation. The RNA design involves a 12‐nucleotide‐long core duplex (A : a, green), with two auxiliary handles (grey). The closing base‐pair (X : Y) is C : G, G : C, U : A, A : U, U : G, or G : U, in six parallel reactions. The randomized 3′‐overhang is denoted by NNNN (N representing A, C, G, and U). The 5′‐phosphate is converted to a 5′‐phosphorimidazolide for non‐enzymatic ligation. For detailed sequence information, see Table S1. B) Deconvolution of loop‐closing ligation and nicked‐duplex ligation through a four‐strand sequencing assay. P2 and P3 originate exclusively from nicked‐duplex ligation. The percentage of P2 and P3 relative to the sum of P1, P2, P3, and P4 provides a quantitative estimate of the (lower limit) contribution of nicked‐duplex ligation to total ligated products. Sequencing readout of P1 at low input strand concentration approximates loop‐closing ligation, while P2 and P3 represent profiles of nicked‐duplex ligation at any strand concentrations.

Loop‐closing ligation creates RNA hairpins from unstructured single strands that anneal to form short duplexes with single‐stranded overhangs, followed by a cross‐strand ligation to close the loop (see Figure 1A, top pathway).^[12]^ The RNAs required for loop‐closing ligation are short, rendering them accessible prebiotically.[^23^, ^24^] Loop closing ligation assembles RNA hairpin structures in a single step and is directed by the internal structure of the targeted RNA without the need for an external template. It inherently avoids template inhibition and the requirement for a hairpin RNA to act as unfolded template.[^10^, ^11^] Importantly, the assembled structure is heritable, but only its unstructured precursor fragments must be replicated.

In this study, we systematically explored the sequence‐reactivity profiles of the non‐enzymatic assembly of RNA tetraloops through loop‐closing ligation, in competition with splinted ligation, utilizing high‐throughput RNA sequencing.^[25]^ We found that hairpin structures with loop sequences UNNG, CNNG, and GNNA (where N represents A, C, G, or U) are formed efficiently and selectively. These sequence selectivity patterns persist even when more than half of the overhang sequences are partitioned to splinted ligation. The formation of favored versus disfavored tetraloops exhibited reactivity differences exceeding 300‐fold for certain sequences (e.g., between UNCG and RNNY, where R represents A or G, and Y represents C or U). These high‐efficiency loop‐closing sequences significantly overlap with the most common biological tetraloops, such as the conserved UNCG and GNRA motifs found in ribosomal RNAs.^[22]^ In contrast, competing splinted ligation products favored CG‐rich overhangs, displaying an inverse correlation with common tetraloops. Our results suggest that, at low strand concentrations, a one‐step chemical process could have selectively generated elementary RNA structures with sequences prevalent in biological systems. We discuss the implications of our findings for the emergence of functional RNAs during the onset of life, as well as other applications of the sequence rules established here.

## Results and Discussion

### Investigating RNA Loop‐Closing Ligation by High‐Throughput RNA Sequencing

We have focused on RNA tetraloops due to their prevalence in biological RNAs.[^4^, ^22^] However, the principles outlined here also apply to the study of other RNA secondary structures, such as bulge loops and internal loops. In our experimental setup, the positions within the hairpin structure that we evaluated included both the unpaired loop region (denoted as NNNN, where N represents A, C, G, or U; Figure 1A) and the closing base pair (X : Y, involving either Watson–Crick or wobble pairing)^[22]^. For loop‐closing ligation, we disconnected the hairpin tetraloop so as to leave the loop nucleotides as a 3′‐overhang (5′‐NNNN‐3′) with a 5′‐phosphate on the other strand of the duplex (Figure 1A, boxed). The stem region, consisting of 12 base pairs, was kept constant except for the closing base pair. Thus, the total number of sequence variants that we considered is 1536, given that there are 6 possible closing base pairs for each of the 256 randomized 4‐nt overhang sequences. We employed multiplexed, high‐throughput RNA sequencing to map the complete sequence‐activity profile for these variants across six parallel reactions, each featuring a unique closing base pair.

The RNA construct that we designed for sequence‐activity profiling consists of a core duplex with overhangs on both ends (Figure 1A, duplex A : a, highlighted in green). The duplex stem was designed to maintain full occupancy even at picomolar concentrations. The loop‐closing ligation junction is at the right‐hand side of the core duplex (blue font), with the 5′‐phosphate activated as a phosphorimidazolide. At the opposite (left hand) end, two sequence‐defined RNA handles (indicated in grey) were installed for subsequent reverse transcription (RT) and polymerase chain reaction (PCR) processes (see Table S1). To minimize undesired interference with the loop closing reaction, the handle overhangs are blocked by including complementary DNA oligonucleotides in the reaction mixture (dark grey in Figure 1A). Upon covalent closure of the nicked loop (the top pathway in Figure 1A), the ligation product can be easily separated from starting materials and amplified using the pre‐installed handles as RT and PCR primer binding sites (Figure S1). The reactions of loop‐closing ligation were incubated at 23 °C until all the 5′‐phosphorimidazolide was consumed by both ligation and hydrolysis.

Randomized single‐stranded overhangs are expected to dynamically anneal with complementary overhangs, potentially leading to splint‐directed (or nicked duplex) ligation in a concentration dependent manner (Figure 1A, bottom pathway). We used this concentration dependence to assess the competition between loop‐closing and splinted ligation processes, in a mixed one‐pot reaction. However, the sequencing readout alone cannot distinguish between the products of loop‐closing and splinted ligation since they yield same sequence products (A‐a in Figure 1A). Although the products of loop‐closing ligation and splinted ligation could be separated and purified by native PAGE (polyacrylamide gel electrophoresis) based on their distinct structure and size, we instead devised an alternative sequencing strategy to quantitatively monitor both reactions in one experiment (Figure 1B). In this design, strands B and b form the duplex B : b, while strands A and a form the duplex A : a. Each duplex A : a and B : b generates distinct products P1 (A‐a) and P4 (B‐b), respectively, resulting from both loop‐closing ligation and splinted ligation (Figure 1). Nicked duplex intermediates SL (Figure 1B), formed from dimerization of duplex A : a and B : b, generate the splinted ligation products P2 (A‐b) and P3 (B‐a) without any contribution from loop‐closing ligation. The distinct sequences of P1, P2, P3, and P4 enable direct quantification of the ratio of P2 and P3 to the total (P1+P2+P3+P4), which sets the lower limit of contribution of splinted ligation to the total ligation products since P1 and P4 also partially contribute to splinted ligation products. At sufficiently low input strand concentrations, when splint‐directed ligation is insignificant, P1 approximates the loop‐closing ligation profile while P2 (or P3) characterizes the splinted ligation.

### Impact of 3′‐Overhang NNNN Sequence on Loop‐Closing Ligation

**Figure 2:**
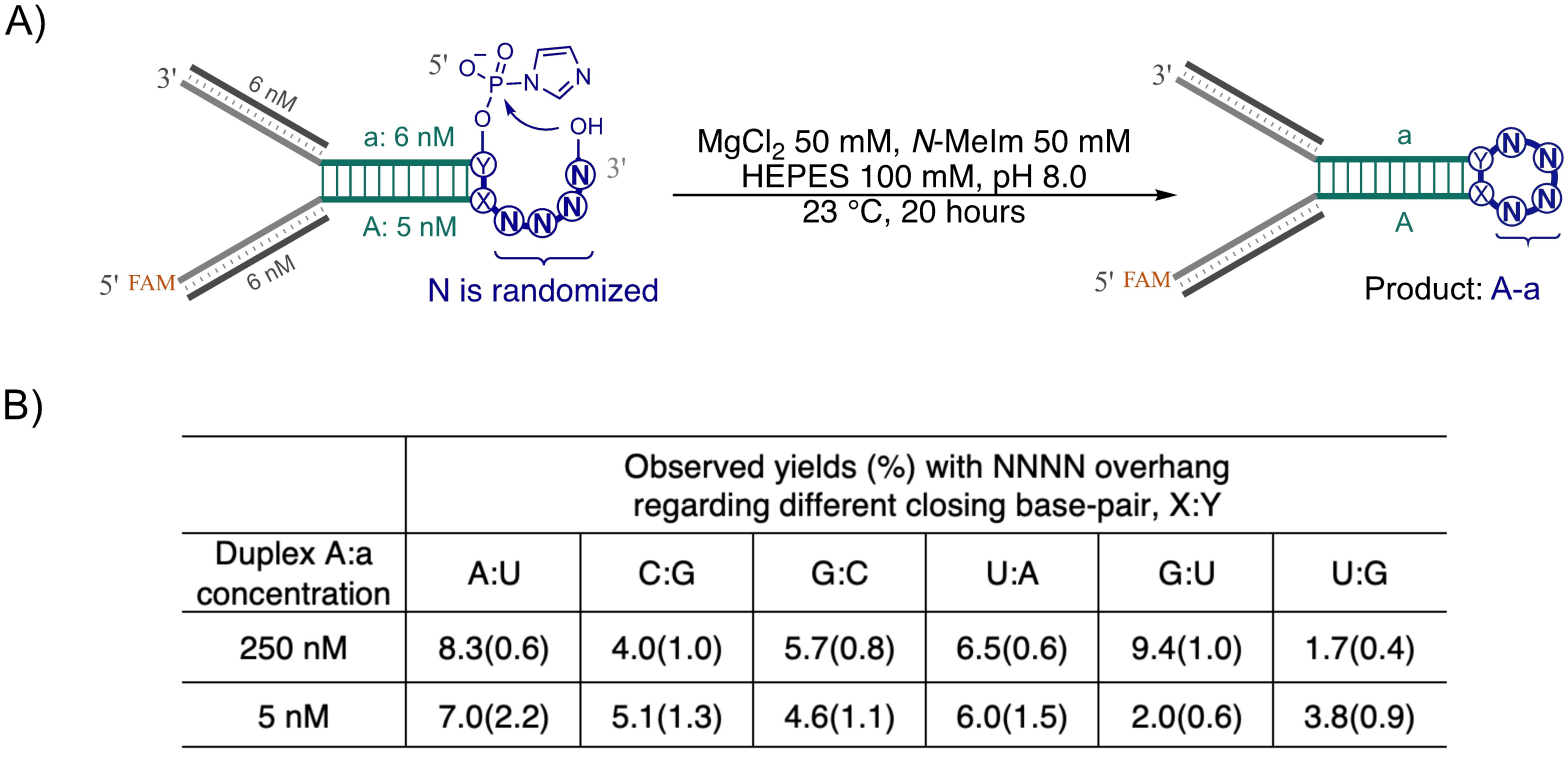
Impact of randomized 5′‐NNNN‐3′ overhangs in combination with different closing base‐pairs (X : Y) on loop‐closing ligation efficiency. A) Reaction Scheme and conditions for loop‐closing ligation. Strand a is in slight higher concentration than strand A to make the desired duplex A : a concentration. B) Observed yields of loop‐closing ligation with randomized NNNN in combination with X : Y. Yields are the average values (%) from triplicates, with standard deviations indicated in brackets.

We first assessed the efficiency of loop‐closing ligation with randomized overhangs in combination with each of the six closing base pairs (Figure 2A). The ligation yields varied from 2 % to 9 % after 20 hours when duplex A : a was at a concentration of 250 nM (Figure 2B and Figure S2). No significant decrease in ligation product was observed (ranging from 2 % to 7 %, Figure 2B and Figure 2C) when the concentration of A : a was reduced to 5 nM, a condition under which less splinted ligation product is expected (Figure 1A). The consistent yields across different duplex concentrations suggest that an increase in loop‐closing ligation product compensates for the reduced splinted ligation product at lower concentrations. The observed yields with a randomized overhang, particularly in the 5 nM reactions (Figure 2B), indicate that many overhang sequences may lead to efficient loop‐closing ligation.

**Figure 3:**
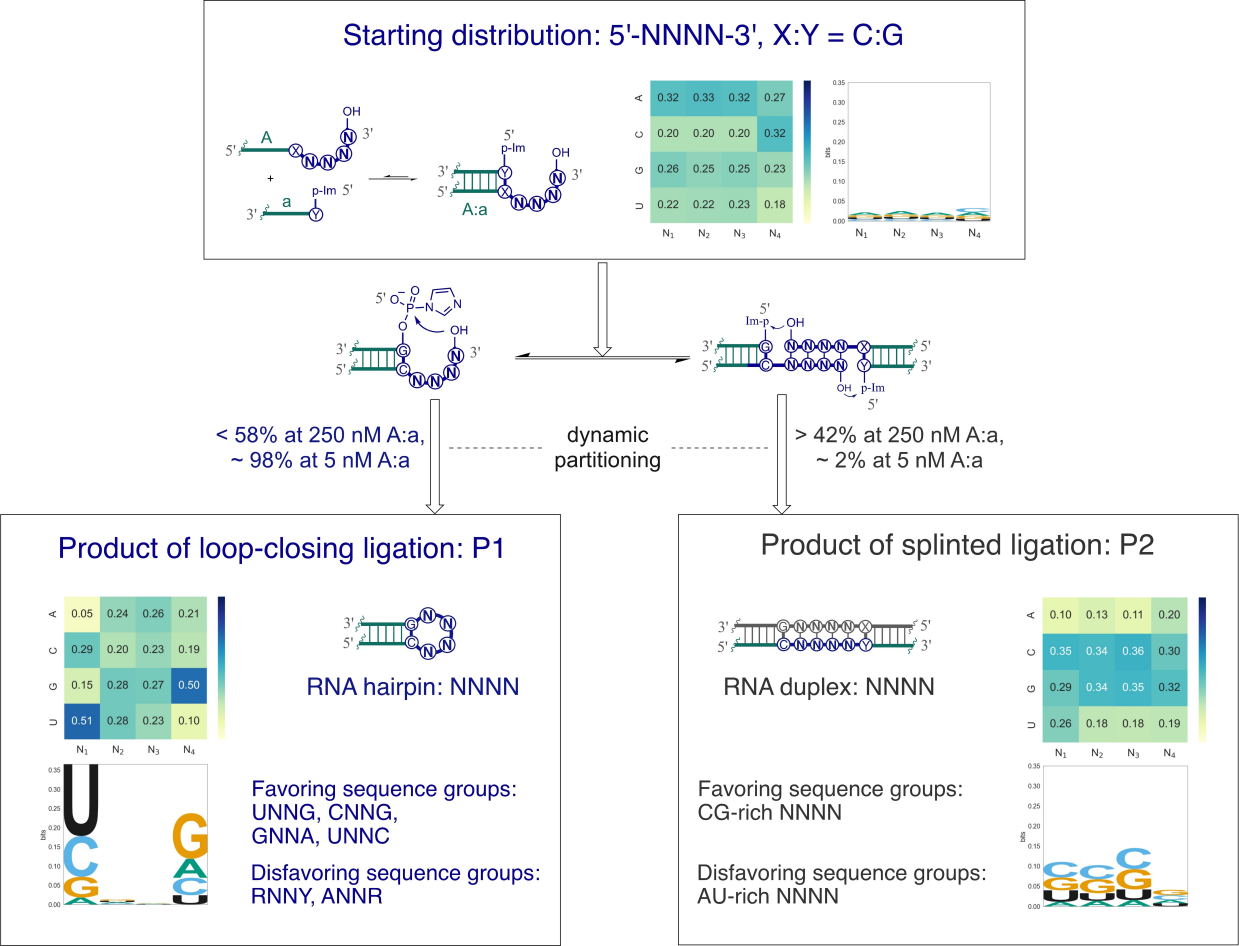
Sequence profiles of the starting material and ligated products of loop‐closing ligation and splint ligation. Illustrates the sequence changes of 5′‐NNNN‐3′ from starting material to ligated products, using reaction C : G as a representative result of the six reactions. Heat maps and sequence consensus logos, generated using the NGS data of all the 254 sequence readout, demonstrate the change of information content (from 0 to 2 bits) of each position. The partitioning of the 256 NNNN sequences between the two ligation pathways is highly dependent on input strand concentration. Major consensus sequence groups of the favored and disfavored sequences for both ligation processes are summarized.

Given that the randomized NNNN sequences produced by the DNA synthesizer are unlikely to be evenly distributed and may differ between batches, it is crucial to understand the input sequence distributions, in order to normalize the sequencing data from the ligation reactions. To this end, we ligated the randomized overhang 5′‐NNNN‐3′ nearly to completion (92 % to 96 % yields) using T4 RNA ligase 2 and a 5′‐preadenylated DNA phosphate donor strand (Figure S3). The ligated products were subjected to deep sequencing (Figure S1, and Supplementary methods), and sequence distributions were derived based on the read counts for each unique NNNN sequence (Table S2). We found that the base composition for the four strands with NNNN overhangs was A_0.33_>G_0.26_>U_0.22_>C_0.19_ (lower case number represents the average base frequency) for the first three positions and C_0.33_>A_0.28_>G_0.21_>U_0.18_ for the fourth position (Figure 3, top panel, Figure S4). The distinct base composition of the fourth position likely arises from it being the sole N derived from phosphoramidites reacting with the controlled pore glass support. The frequency of the most and least common sequences varied by 12‐fold on average (range from 8 to 20‐fold, Figure S5) across the four RNA strands with NNNN overhangs (Table S2), which would introduce a significant bias if an even distribution were assumed. Consequently, normalization factors (α_i_, where i ranges from 1 to 256) for each unique sequence were introduced (Table S2), calculated from the difference in their observed frequency compared to a hypothetical even distribution (e.g., α_i_=1 when the observed frequency is 1/256), to accurately calibrate the output of the ligation experiments.

### Sequence‐Activity Profiles for Loop‐Closing Ligation

We conducted six parallel reactions by mixing the strands B, b, A and a, forming duplex B : b with one of six different A : a duplexes for each reaction (Figure 1B, details see Methods in SI), at concentrations of either 5 nM or 250 nM. Observed ligation yields ranged from 5 % to 10 % after 20 hours (Figure S6). Each reaction is named after its closing‐base pair, for instance, for reaction C : G the closing‐base pair X : Y in duplex A : a is C : G. Following the sequencing workflow described above, we first analyzed the unnormalized sequencing reads for each reaction. As previously discussed, we assessed the contribution of splinted ligation to the total ligation products by calculating the ratio, $\frac{P2+P3}{P1+P2+P3+P4}$ (Figure 1B). At 250 nM concentration, P2 and P3 account for 44 % (ranging from 39 % to 49 %, Table S3) of total reads across the six reactions, indicating the lower limit of the contribution of splinted ligation to the total ligated products. Under these conditions, P2 sequences can be used to characterize the profile of splinted ligation, however, a significant fraction of the P1 and P4 sequences are expected to result from splinted ligation, making it impossible to evaluate the sequence preferences of loop‐closing ligation.

Given the concentration dependence of splint‐directed ligation, we reduced the reaction concentration to 5 nM. At this concentration, both A : a and B : b maintain full core duplex formation, and each unique overhang sequence is present at only 0.02 nM. Under these conditions the contribution of splinted ligation drops to 3 % (ranging from 2 % to 6 %, Table S3). We therefore proceeded at this concentration since any further decrease in concentration would require impracticably large reaction volumes. At the 5 nM concentration, P1 sequences (A‐a) serve as a proxy for loop‐closing ligation, while P2 sequences (A‐b) represent the sequence‐activity profile of splinted ligation, at both 250 nM and 5 nM concentrations. Additionally, six reactions using only duplex A : a at 5 nM were sequenced alongside the four‐strand reactions (Table S4).

To derive a more accurate profile for loop‐closing ligation, we normalized the tetraloop products P1 (A‐a) from the 5 nM reactions to the abundance of the corresponding input sequences. All 1536 possible NNNN sequences were present in the sequencing readouts, with the number of reads decreasing logarithmically based on their ranking orders for all reactions (Figure S7). Even the least abundant sequences had tens to hundreds of reads (Table S5), indicating that each possible overhang sequence could undergo loop‐closing ligation. Furthermore, enough sequences were obtained to enable quantitative interpretation. The frequency distribution of NNNN in P1 was much broader than the initial sequence distribution (Figure 3, Figure S8). For example, the frequency range among the starting NNNN sequences (12‐fold in average, Table S2) expanded to 70 to 280‐fold among the loop‐closing products P1 (Table S5), indicating significant variability in reactivity among different overhang sequences. Heat maps of base composition showed that the first position of P1 was heavily enriched for U and depleted for A, while the fourth position was enriched for G and depleted for U (Figure 3, bottom left panel, and Figure S7). Accordingly, the first and fourth positions showed more conservation than the second and third positions, as depicted by the sequence consensus logos (Figure 3).

When analyzing the P1 sequences ranked according to their decreasing frequency, we focused on the top 40 sequences for each of the six reactions (Table S5). These sequences, covering approximately 50 % of the total reads in each reaction, were expected to be particularly high‐yielding for loop‐closing ligation. Surprisingly, most of the top 40 sequences fell into three major consensus sequence groups: UNNG, CNNG, and GNNA (N representing A, C, G, or U), regardless of the closing base‐pair of the core duplex. This sequence conservation pattern is clearly reflected in the consensus logos (Figure 3, bottom left panel, Figure S7, and top 40 sequences in Table S5). Additional minor sequence groups appeared to be specific to the closing base‐pairs; for instance, seven UNRC sequences were among the top 40 in the C : G reaction, and two UNRC sequences appeared in the U : G reaction. Nine UNNA sequences were scattered among the top 40 in the G : U and A : U reactions (top 40 sequences in Table S5). Conversely, the bottom‐ranking 40 sequences, which we assumed to be the least efficient overhangs for loop‐closing ligation, could be grouped into consensus patterns of RNNY, ANNR, and CNNU (R for A or G, Y for U or C), with few exceptions (Bottom 40 sequences in Table S5).

Similar P1 sequence patterns persisted even in four‐strand reactions with 250 nM each of A : a and B : b (Table S6), except for some new CG‐rich sequences appearing among the most abundant sequences, when splinted ligation contributed at least 44 % of total products (Table S3). For instance, sequences such as GGCG, GCGG, GCCG, and GGGG were among the top 40 sequences of P1 from the 250 nM reaction with a C : G closing base‐pair, but were not observed in the 5 nM reaction (Table S6). The comprehensive sequence abundance profiles suggest that loop‐closing ligation favorably converts tetranucleotide overhangs with the sequence patterns of UNNG, CNNG, and GNNA into hairpin tetraloop structures, regardless of the closing base‐pairs while overhangs such as RNNY, ANNR, and CNNU are much less likely to be present in the tetraloop products. Additionally, similar sequence patterns of loop‐closing ligation persist even under conditions when almost half the total reacted overhangs partitioned to splinted ligation, demonstrating the robustness of sequence and structural selectivity through loop‐closing ligation.

### Sequence‐Activity Profiles of Competing Nicked‐Duplex Ligation

The emergence of CG‐rich sequences among the top 40 P1 sequences in reactions with 250 nM duplex suggests a distinctive pattern of splinted ligation. From a systems chemistry perspective, complementary overhangs inevitably anneal to form duplexes in a complex mixture, leading to competition between splint‐directed ligation and loop‐closing ligation (Figure 1A). To elucidate the sequence profile characteristic of splinted ligation, we analyzed the P2 (A‐b) products at a 250 nM concentration. Nearly all the top 40 NNNN sequences were CG‐rich, containing at least three Cs and/or Gs (Figure 3, bottom right panel, Figure S9, and Table S7), echoing the preceding findings (Table S6). Notably, the bottom 40 sequences were predominantly AU‐rich, leading to a sequence consensus logo characterized by the prevalence of CG over AU globally (bottom right panel in Figure 3 and Table S7).

We propose that the formation of the four‐nucleotide duplexes may be a determining factor in the efficiency of splinted ligation, as C : G base pairs are inherently stronger than A : U pairs. Although the representation of P2 was low in reactions at 5 nM, similar sequence patterns were still evident (see Supplementary excel file). In summary, by disentangling loop‐closing ligation and its competing splint‐directed ligation, we see that the sequence‐reactivity profile of splinted ligation is notably simpler and distinctly different from that of loop‐closing ligation.

### Quantitative Validation of the Sequence‐Reactivity Profile of Loop‐Closing Ligation

**Figure 4:**
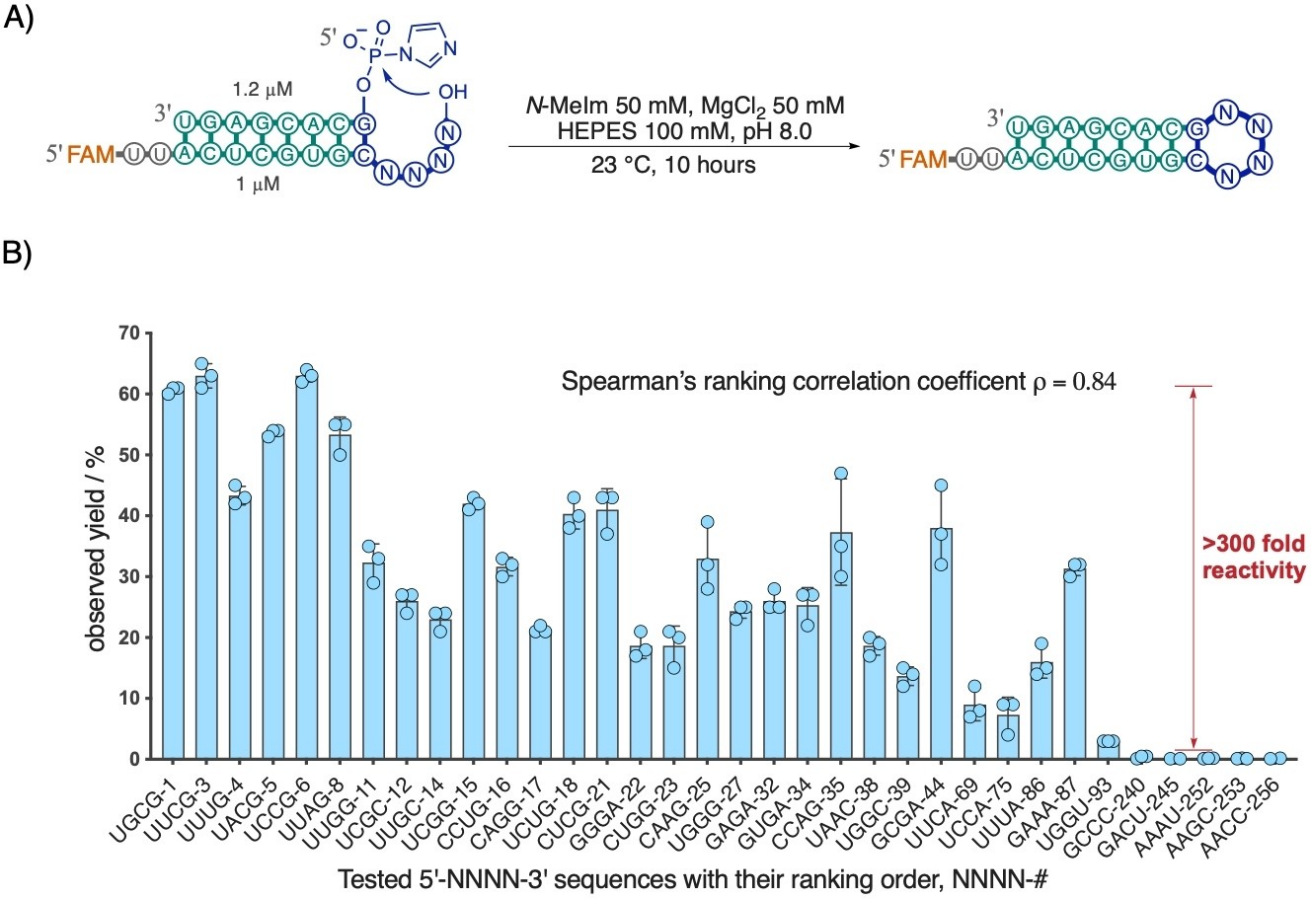
Correlation of ligation efficiency with ranking order in NGS readout. A) Experimental setup and conditions for testing individual overhangs from sequencing the result of reaction with a C : G closing base‐pair. B) Efficiency of individual overhangs in loop‐closing ligation against their decreasing rank order in the NGS readout. Yields (%) represent averages from triplicate experiments. Spearman's ranking correlation coefficient was calculated to be 0.84.

While the number of reads for each overhang sequence in our sequencing assay serves as a proxy for its loop‐closing ligation efficiency, it is crucial to experimentally validate whether the sequence ranking order indeed correlates with the actual efficiency of loop‐closing ligation. To this end, we selected overhang sequences from the P1, GC set (A : a) to test for loop‐closing ligation efficiency (Figure 4A). We individually tested 34 out of 256 sequences (Figure 4B), aiming to cover representative sequence groups from the top (UNNG, GNRA, CNNG, UNNC), bottom (RNNY), and middle (such as UUCA and UGGU) (Table S5) of the overall ranking distribution.

Among the tested sequences, the four UNCG (N for A, C, G, and U) overhang sequences, which ranked among the top 10, exhibited an average observed yield of 62 % (Figure 4B). The sequences CNNG, GNNA, and UNNC yielded averages of 30 %, 28 %, and 20 %, respectively. In strong contrast, bottom‐ranking sequences with an RNNY consensus yielded an average of only 0.2 % ligation. The 310‐fold difference in the observed yield of loop‐closing ligation between the UNCG and RNNY sequences closely matches the 140‐fold difference in their NGS read frequencies (Table S5). Consistent with this observation, we observed a strong Spearman's rank correlation (coefficient ρ=0.83, Figure 4B and Appendix S1) when analyzing the efficiency of loop‐closing ligation against the ranking of the 34 tested overhang sequences. These results show that the sequencing read rankings correlate well with the efficiency of loop‐closing ligation.

### Self‐Assembly of Functional RNAs by Iterated Loop‐Closing Ligation

**Figure 5:**
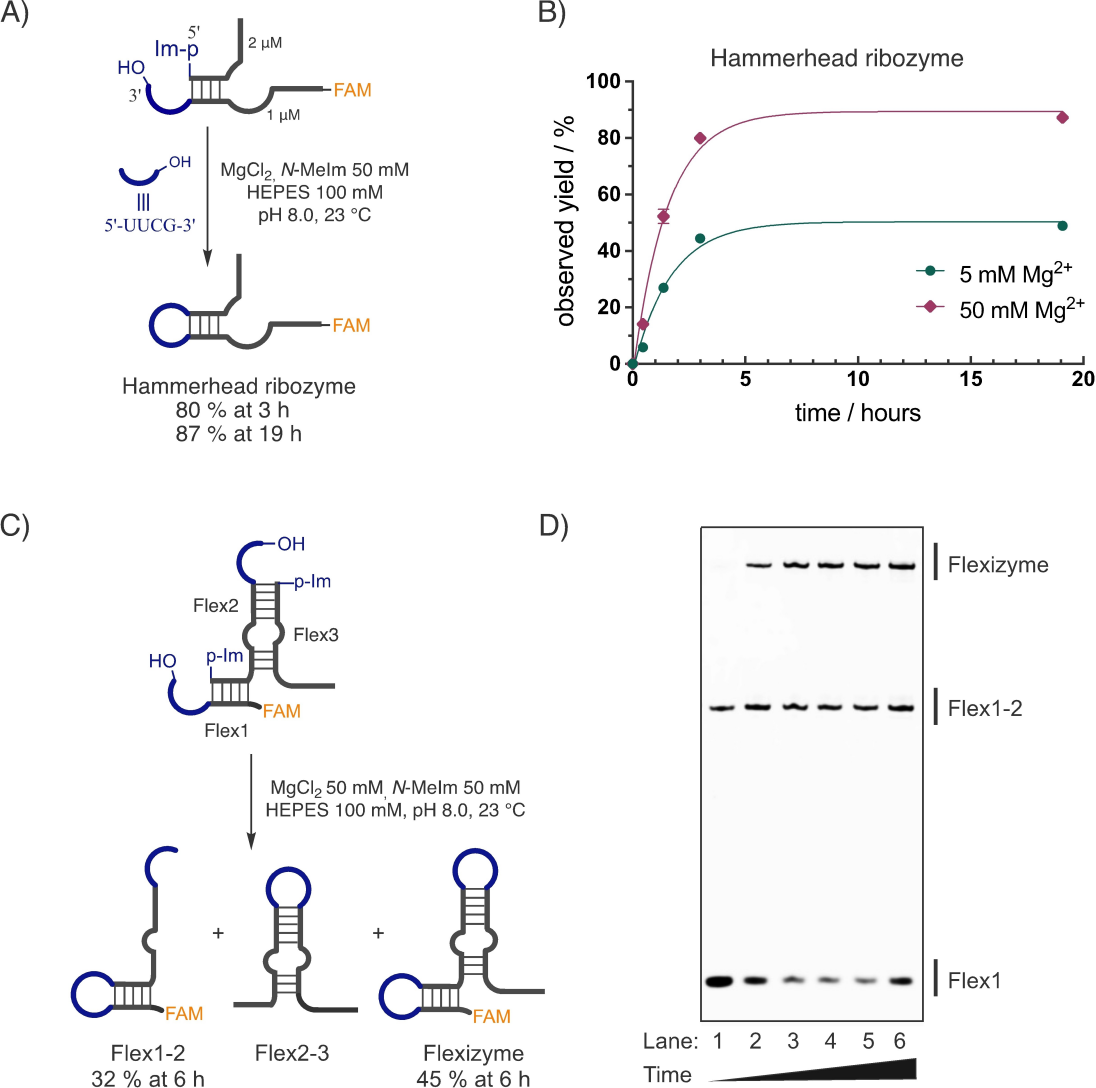
Template‐free assembly of functional RNAs using overhang sequences for high‐efficiency loop‐closing ligation. A) Self‐assembly of the hammerhead ribozyme facilitated by a single loop‐closing ligation, with a 5′‐UUCG‐3′ overhang engineered into the product hairpin loop sequence. B) Time courses and MgCl_2_ dependence of the hammerhead ribozyme self‐assembly. C) Self‐assembly of the Flexizyme facilitated by two concurrent loop‐closing ligations. D) Representative denaturing PAGE gel of Flexizyme assembly, with lanes indicating reaction quenched at time points, 0.5 h, 1.5 h, 3 h, 5 h, 6 h and 24 h. Uncropped gel image can be found in the Supporting Information.

An immediate benefit of knowing the sequence‐activity profile for loop‐closing ligation lies in facilitating high‐yielding, template‐free assembly of functional RNAs. Prior attempts to self‐assemble functional ribozymes achieved roughly 10 % yield through one loop‐closing ligation over a 10 hour period, limited by arbitrary choices of loop sequences.^[12]^ By strategically incorporating the UUCG sequence into the hairpin loop region of the hammerhead ribozyme (Figure 5A), we observed an 80 % yield of assembly of full‐length ribozyme from two fragments within 3 hours by a single loop‐closing ligation, with this yield increasing to 87 % after 19 hours (Figure 5B and Figure S10). Similarly, the full‐length Flexizyme aminoacyl‐RNA synthetase ribozyme was assembled in 45 % yield after 6 hours from three fragments by two simultaneous loop‐closing ligations, with the two individual loops being closed in 77 % and 58 % yields (Figure 5C, Figure 5D, and Figure S11). This enhanced efficiency underscores the critical role of optimal loop sequence selection in the template independent assembly of functional RNA structures.

### Overlap of High‐Efficiency Loop‐Closing Sequences and Common Biological Hairpin Tetraloops

The high‐efficiency sequence groups, UNNG, CNNG, and GNNA, in the products of loop‐closing ligation resemble the common tetraloops UUCG and GNRA that are conserved in ribosomal RNAs.[^4^, ^22^] Utilizing our quantitative sequence‐reactivity profiles for constructing RNA tetraloops, we explored how the top‐ranking sequences from both loop‐closing and splinted ligations overlap with common biological tetraloops. We note that splinted ligation products can rearrange to hairpin structures through additional strand separation and folding steps (Figure 1A). To begin, we categorized biological tetraloops from the automated RNA secondary structure database, bpRNA‐1 m,^[4]^ into six groups based on their differing closing base‐pairs. Sequences with more than 90 % similarity were removed, maintaining at least 70 % alignment coverage, to reduce duplicate counting. The sequences within each group were then ranked by frequency (Table S8). We then compared the top 40 sequences from loop‐closing ligation (or splinted ligation) with the top 40 biological tetraloops for each closing base‐pair group.

**Figure 6:**
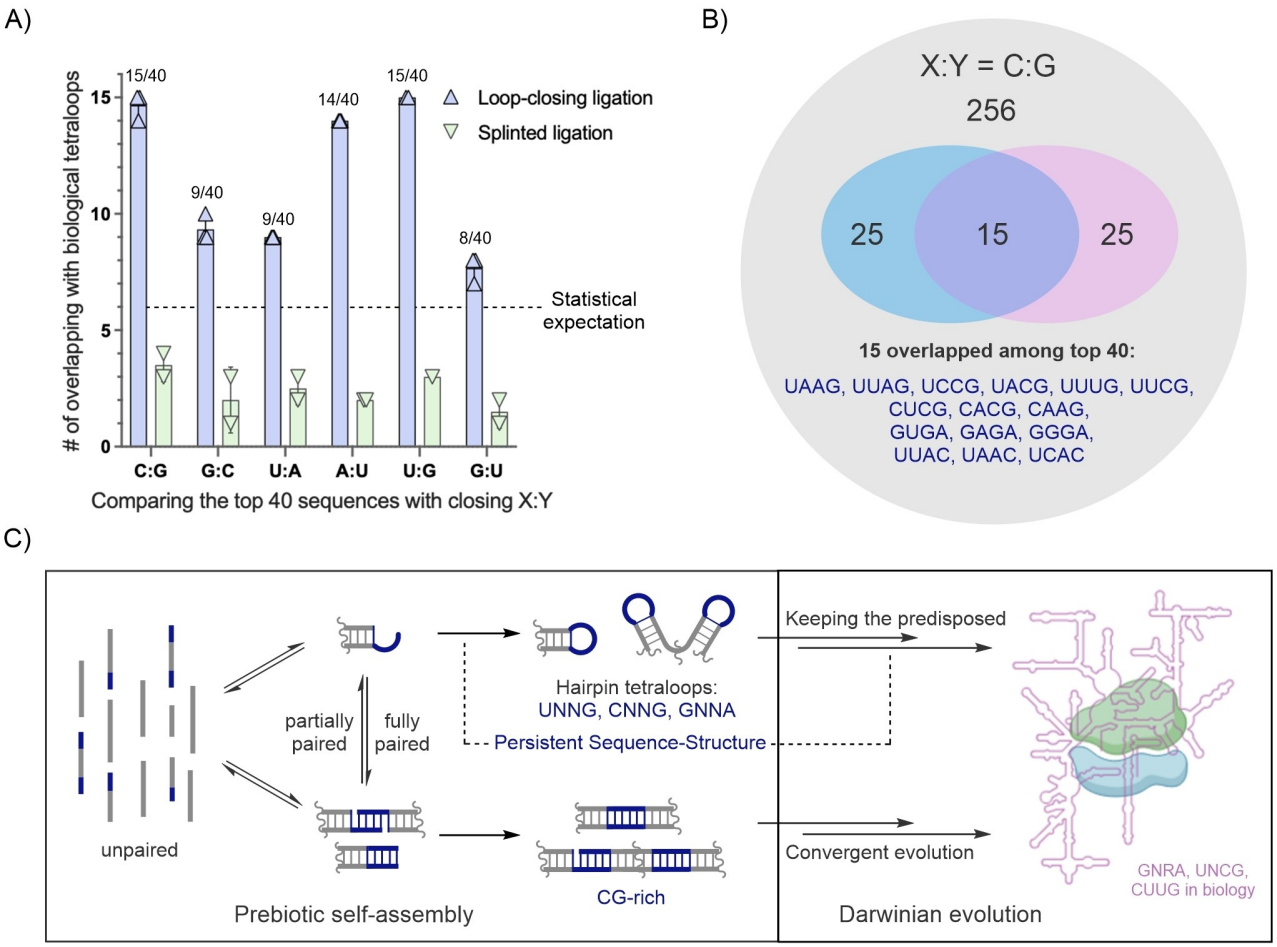
Shared sequence selectivity between high‐efficient loop‐closing ligation sequences and common biological tetraloops. A) Comparison of the top 40 sequences of ligated products from loop‐closing ligation and splint ligation with the corresponding top 40 biological tetraloop sequences. For unrelated datasets, the statistically expected overlap is 6, indicated by a dashed line. B) Representative Venn diagram illustrating 15 overlapped sequences from the reaction with a C : G closing base‐pair. The grey circle represents all 256 tetraloops, while the blue and pink circles represent the top 40 sequences from loop‐closing ligation and biological RNAs, respectively. C) Depiction of a plausible and economical pathway for the emergence of functional RNAs via loop‐closing ligation, in which a persistent chemical interaction might have underpinned the shared sequence selectivity.

For the loop‐closing ligation with a C : G closing base‐pair, an average of 15 overlapping sequences was observed across three independent sequencing experiments (Figure 6A), with detailed results illustrated in representative Venn diagrams (Figure 6B). The overlapping sequences included 6 UNNG, 3 CNNG, 3 GNRA, and 3 UNNC tetraloops. Other data sets such as for A : U and U : G closing base‐pairs exhibited average overlapping sequence counts of 14 and 15, respectively, while closing base‐pairs U : A and G : C showed 9 each, and G : U only had 8 (Figure S12). Statistically, the expected number of overlaps between the two sets of top 40 sequences would be around 6, assuming a random distribution of loop‐closing sequences with biological tetraloops as a fixed reference (analyzed using a hypergeometric distribution, see Supporting Information, Appendix S2). Across the six loop‐closing ligation reactions, the average number of overlaps was 12, twice the expected value, which is unlikely to occur purely by chance (*p*=0.009 by hypergeometric test). We also assessed overlaps between different cutoffs, i.e. the top 20, top 30, and top 50 sequences—from loop‐closing ligation products and the biological tetraloops. These comparisons consistently showed a roughly 2‐fold over‐representation compared to expectations (Figure S13).

Conversely, the top 40 sequences from the splinted ligation data sets, when compared to their corresponding biological tetraloops, averaged only 2 overlapping sequences (Figure 6A), suggesting a significant under‐representation (3.1‐fold less than expected, *p*=0.03, Figure S14), likely due to the preference of splinted ligation for CG‐rich sequences (Table S7). This analysis emphasizes that non‐enzymatic self‐assembly of short RNAs with tetranucleotide overhangs via loop‐closing ligation selectively produces stem‐loop hairpin RNAs that resemble biological stem‐loops in their loop sequences. Meanwhile, products of splinted ligation showed an inverse correlation with biological sequences.

### Discussion

We have explored the sequence‐reactivity profiles of all 256 tetranucleotide overhang sequences in the context of 6 different closing base‐pairs in forming RNA hairpin structures through loop‐closing ligation. We also examined the tendency of these sequences to undergo splinted (or nicked duplex) ligation. By distinguishing between these two pathways, in a competitive scenario, we obtained distinct profiles for the two pathways (Figure 1 and Figure 2). Under both loose and stringent competition, the loop‐closing ligation profile indicated that hairpin structures with loop sequences of UNNG, CNNG, and GNNA (N representing A, C, G, or U) are efficiently and selectively formed (Figure 3). In contrast, sequences such as RNNY, ANNR, and CNNU were disfavored in loop‐closing ligation, with reactivity differences exceeding 300‐fold for certain sequences (Figure 4), such as between UNCG and RNNY, when C : G is the closing base‐pair. Such dramatic differences are consistent with certain sequences having the potential to be spatially pre‐organized so as to favor loop‐closing ligation.^[12]^ In contrast, when the overhang sequences anneal and undergo splinted ligation, the products showed a preference for CG‐rich overhangs and disfavored AU‐rich ones. By using sequences that confer efficient loop‐closing, we achieved up to 87 % yield in self‐assembling the hammerhead ribozyme through single loop‐closing ligation, and a 45 % yield in assembling the full‐length Flexizyme via two loop‐closing ligations (Figure 5). We anticipate that the high‐yielding self‐assembly of complex ribozymes, driven by in situ activation chemistry and independent of any template,[^26^, ^27^] should be feasible in laboratory studies by leveraging the sequence rules revealed in this study. Similarly, we expect that the assembly of ribozymes from smaller replicating RNA fragments should have been possible in early protocells and would have been favored with the sequence overhangs identified here.^[28]^

We observed a significant overlap between high‐efficiency loop‐closing overhangs and the most common biological tetraloops, such as UUCG and GNRA (Figures 6A and Figure 6B). UUCG and GNRA tetraloops are thermodynamically stable due to hydrogen bonding and base‐stacking interactions within the loop regions,[^29^, ^30^] making them likely targets of biological selection over billions of years of evolution.^[31]^ Our results suggest, for the first time, that these structurally and functionally exceptional biological tetraloops may have been predisposed in an early RNA pool, facilitated by a sequence‐selective, self‐assembly pathway. We propose that the shared sequence selectivity observed in prebiotic loop‐closing ligation and common biological sequences might be governed by conserved non‐Watson–Crick interactions embedded within short unpaired RNA sequences. This sequence‐structural relationship manifests in biological RNAs as hairpin loops closing the ends of stems and in prebiotically plausible self‐assembly processes through RNA loop‐closing ligation (Figure 6C).

Several recent studies suggest that the preorganization of RNA stem‐overhang structures may be broadly significant in understanding the evolution of RNA structure.[^12^, ^32^, ^33^, ^34^, ^35^] For example, the Sutherland group has recently examined RNA stems with 5‐nt 3′‐overhangs for the transfer of amino acids from the 5′‐phoshate to the 2′,3′‐*cis* diol of the overhang.^[32]^ Their results suggest that the specificity of aminoacyl transfer is encoded within the base‐paired stem.[^33^, ^34^] In contrast, recent work from our laboratory indicates that an RNA stem with a particular 7‐nt overhang can specifically capture activated glycine, leading to loop‐closing ligation with a bridging glycine in the RNA backbone.^[35]^ Considering that hairpin pentaloops and heptaloops are the most prevalent hairpin structures after tetraloops in biology,[^4^, ^22^] it would be interesting to map the sequence‐reactivity profiles for pentaloop and heptaloop formation through loop‐closing ligation in the future. Moreover, short oligonucleotides with an alternative alphabet, rather than ribonucleosides A, C, G and U, might exhibit very different sequence‐reactivity relationships. Such systematic studies will not only shed light on questions about the origins of life but also enhance our understanding of the intricate relationship between RNA sequence and structure in general.

## Conclusion

Our work has elucidated the sequence‐reactivity rules governing the self‐assembly of RNA tetraloop structures via template‐free loop‐closing ligation, while also revealing a notable convergence with prevalent biological tetraloops. We suggest that interactions within unpaired short RNA sequences constitute a cryptic structural code underlying the shared sequence selectivity of loop‐closing ligation and biological tetraloops. Based on the results, we suggest that a library of structurally constrained RNAs could serve as a possible primordial repository for the emergence of RNA functions and subsequent Darwinian evolution. The robust yields achievable through high‐efficiency, template‐free loop‐closing ligation may also be of utility in nucleic acid engineering, such as the construction of cyclic RNAs and other difficult to access structures.^[36]^

## License Information

This article is subject to HHMI's Open Access to Publications policy. HHMI lab heads have previously granted a nonexclusive CC BY 4.0 license to the public and a sublicensable license to HHMI in their research articles.

## Author Contributions

LFW and JWS conceived the study. LFW designed (except the Flex1, Flex2, and Flex3 sequences), and conducted wet‐lab experiments. JZ developed the Python program for processing raw NGS data, while LFW and JZ jointly analyzed and interpreted the NGS results. RCA and DAH statistically compared biological tetraloops with tabulated final NGS results. AR designed and synthesized the sequences of Flex1, Flex2, and Flex3. LFW illustrated the results. JWS supervised the project and LFW and JWS drafted the manuscript with contributions from all other authors.

## Conflict of Interests

The authors declare no competing interests.

1

## Supporting information

As a service to our authors and readers, this journal provides supporting information supplied by the authors. Such materials are peer reviewed and may be re‐organized for online delivery, but are not copy‐edited or typeset. Technical support issues arising from supporting information (other than missing files) should be addressed to the authors.

Supporting Information

## Data Availability

The code for general deep sequencing analysis can be found at: https://github.com/szostaklab/loop‐closing‐ligation‐seq. All other data are presented in the main text or Supporting Information. Raw sequencing data are available upon request.
