# Supplementary material for: Selective Nonenzymatic Formation of Biologically Common RNA Hairpins
Source: Angew Chem Int Ed Engl. 2024 Nov 26;64(5):e202417370. doi: 10.1002/anie.202417370 (PMC11773311; doi:10.1002/anie.202417370)

Angewandte Chemie
Eine Zeitschrift der Gesellschaft Deutscher Chemiker

Supporting Information

# Selective Nonenzymatic Formation of Biologically Common RNA Hairpins

*L.-F. Wu, J. Zhang, R. Cornwell-Arquitt, D. A. Hendrix, A. Radakovic, J. W. Szostak**

## Supporting information for

# Selective Nonenzymatic Formation of Biologically Common RNA Hairpins

Long-Fei Wu[1, †], Junting Zhang[2], Robert Cornwell-Arquitt[3], David Hendrix[3,4], Aleksandar Radakovic[1] and Jack W. Szostak [1,*]

[1] Howard Hughes Medical Institute, The University of Chicago, Chicago, IL 60637, USA.

[2] The University of Chicago, Chicago, IL 60637, USA.

[3] Department of Biochemistry and Biophysics, Oregon State University, USA

[4] School of Electrical Engineering and Computer Science, Oregon State University, USA

[†]Current address: Frontiers Science Center for Transformative Molecules, School of Chemistry and Chemical Engineering, Shanghai Jiao Tong University, Shanghai, 200240, China

*Correspondence to: jwszostak@uchicago.edu

**The PDF file includes:**

Materials and General………………………………………………………… 2

Supplementary Methods……….…………………………………………..…. 3

Supplementary Figure S1 to S14 ...………………………… …………….. 10

Supplementary Table S1 to S8 …………………………..………………… 24

Appendix S1 to S2 …………………………………………………………… 73

Uncropped, full-size gel images…………………………………………... 74

## Materials and General

For solid-phase RNA synthesis, 1 μmol scale pre-packed synthesis columns were acquired from Glen Research. Phosphoramidites utilized for RNA synthesis were sourced from Glen Research and ChemGenes. RNA oligomers were synthesized using either a K&A H-8 DNA/RNA Synthesizer or an Expedite 8909 at 1 μmol scale, or alternatively purchased from Integrated DNA Technologies (IDT). To synthesize randomized nucleobase in an oligo, an input ration of premixed phosphoramidites of A:C:G:U = 3:3:2:2 was used. The MiSeq Reagent Kit v3 (150-cycle) was obtained from Illumina. T4 RNA Ligase 2 truncated K227Q, ProtoScript II Reverse Transcriptase, and Q5 Hot Start High-Fidelity DNA Polymerase were procured from New England Biolabs (NEB). Amicon® Ultra-4 Centrifugal Filters, 10 kDa MWCO, were acquired from MilliporeSigma. High-pressure liquid chromatography (HPLC) analysis was performed using a Nexera HPLC/UHPLC system (Shimadzu) equipped with an Atlantis™ T3, 3 μm, 4.6 x 150 mm column, and detection was achieved at 260 nm using an SPD-M30A UHPLC Photodiode Array Detector. Polyacrylamide gel electrophoresis was conducted on 8% to 20% polyacrylamide, 8 M urea gels (0.75 mm thick, 20 cm long) at 18 W in 1X TBE buffer (89 mM Tris-borate, 2 mM EDTA, pH 8.3) for 1 to 2 hours. FAM-labelled RNA oligomers were detected and imaged with an Amersham RGB Biomolecular Imager (GE Healthcare Life Science, Marlborough, MA) and quantified using the ImageQuantTM software package (GE Healthcare Life Science, Marlborough, MA). For imaging unlabelled RNA oligos, the gel was stained with SYBR Gold Nucleic Acid Gel Stain (Invitrogen). Oligonucleotide concentrations were determined by UV absorbance at 260 nm using a NanoDrop® ND-1000 spectrophotometer.

## Supplementary Methods

### General procedure for chemical synthesis of 5′-phosphorimidazolide RNA (Im-p-RNA) from 5′-phosphate RNA (5′-p-RNA).

A 300 μL aqueous reaction mixture containing 0.1 mM 5′-p-RNA and 100 mM imidazole (pH 7.0) was prepared. To this mixture, 5.8 mg of EDC was added to achieve a final concentration of 80 mM, and the mixture was incubated at room temperature. After 2 hours, 10 mL of cold 50 mM $NaClO_4$ in acetone was added, creating a cloudy mixture that was vigorously vortexed and then chilled on dry ice for 30 minutes. The mixture was then centrifuged at 4000 rpm for 10 minutes at 4°C, yielding a white pellet. The supernatant was discarded, and the pellet was resuspended in 2 mL of cold 50 mM $NaClO_4$ in acetone, vortexed extensively for 30 seconds, and centrifuged again under the same conditions. This washing step was repeated twice. The pellet was then dried in a SpeedVac under vacuum without heat for 30 minutes. The resulting white pellet was redissolved in 60 μL of 20 mM HEPES buffer (pH 8.0) and stored at -80°C for future use without further purification. The concentration of Im-p-RNA combined with 5′-p-RNA was quantified by UV absorbance at 260 nm using a NanoDrop. The conversion from 5′-p-RNA to Im-p-RNA was assessed by HPLC analysis, employing a UV detection system at 260 nm. The system used an Atlantis™ T3, 3 μm, 4.6 x 150 mm column, with an 8 μL injection of 10 μM RNA solution. The flow rate was set at 0.5 mL/min, utilizing LC solvents comprising 50 mM triethylammonium acetate in water (pH 7.5, solvent A) and acetonitrile (solvent B). The mobile phase gradient was programmed as follows: 6% B from 0 to 2 minutes, adjusting to 20% B (dependent on oligo length) by 20 minutes, escalating to 95% B from 22 to 25 minutes, and reverting to 6% B from 27 to 30 minutes before ending the analysis. The column compartment temperature was maintained at 25°C.

**Loop-closing ligation using randomized NNNN overhangs.**

10 μL reaction mixture, containing phosphate acceptor strand A (5 nM or 250 nM, X = A, C, G, or U, respectively, in strand A, see Table S1), pre-activated phosphate donor strand a ( 6 nM or 300 nM, Y = A, C, G, or U in strand a, see Table S1), Blocker-1 (6 nM or 300 nM), Blocker-2 (6 nM or 300 nM), $MgCl_2$ 50 mM, HEPES 100 mM (pH 8.0) and *N*-methylimidazole 50 mM (MeIm, pH 8.0, added at last), was incubated for 20 hours at 23 °C. 1 μL of each reaction mixture was quenched by adding it to 9 μL of stop solution, which sonsisted of 50 mM EDTA, pH 8.0, in 90 % formamide. Subsequently, 2 μL of the quenched solution was analysed using a 10 % analytic denatured PAGE gel. Yields were quantified based on the relative amounts of FAM-labelled oligomers, as determined by gel imaging.

For sequencing purposes, reactions initiated at a concentration of 5 nM were scaled up to 10 mL under similar conditions (see below for follow-up processes).

**Determining the initial distribution of NNNN overhang sequences.**

The initial distribution of overhang sequences in the as synthesized acceptor strand was determined by T4 RNA ligase catalyzed ligation followed by sequencing (Figure S3). A 22 μL reaction mixture was prepared containing 2 μM phosphate acceptor strand A (X = A, C, G, or U in strand A, as detailed in Table S1) and 5 μM pre-adenylated phosphate donor strand a', along with 1X T4 RNA ligase reaction buffer (NEB: B0216SVIAL) and 20% PEG 8000 (NEB: B1004SVIAL). The mixture was heated to 90°C for 3 minutes and then cooled to 25°C. Subsequently, 3 μL of T4 RNA Ligase 2 truncated K227Q at 200 U/μL (NEB: M0351L) was added, resulting in a final enzyme concentration of 24 U/μL. The reaction was incubated at 25°C for 18 hours. To quench the reaction, 250 μL of stop solution (50 mM EDTA, pH 8.0, in 90% formamide) was added. The quenched solution was then loaded onto a 10% preparative PAGE gel.

**Purification of ligation product of large-scale reactions**

10 mL solution from the large-scale loop-closing ligation was concentrated to approximately 250 µL using an Amicon® Ultra-4 Centrifugal Filter with a 10 kDa MWCO (MilliporeSigma: UFC801024). The concentrated solution was transferred to a 1.5 mL Eppendorf tube, to which 900 µL of cold absolute ethanol and 120 µL of 3 M sodium acetate, pH 5.5, were added. The mixture was then placed in a -80 °C freezer or on dry ice for 30 minutes, followed by centrifugation at 15,000 rpm for 15 minutes at 4°C, after which the supernatant was discarded. The pellet was washed twice with 100 µL of cold 75% ethanol, centrifuged each time at 15,000 rpm for 10 minutes at 4°C, and then dried for 20 minutes under vacuum using a SpeedVac at room temperature. The dried pellet was resuspended in 50 µL of stop solution (50 mM EDTA pH 8.0 in 90% formamide) and prepared for loading onto a preparative RNA PAGE gel. A 10% denatured PAGE gel with a 1.5 mm thickness was cast, featuring 6 wells (well dimensions: 25 mm width by 15 mm depth). The wells were thoroughly cleaned prior to pre-running and loading the samples. RNA samples were loaded, and the gel was run at 20 W for 2 hours after pre-running the gel for 25 minutes at 15 W in 1X TBE buffer. The target band was visualized using a blue light illuminator (Invitrogen) and excised into a 15 mL tube with a clean razor blade. The excised gel slice was physically crushed and mixed with 10 mL of 5 mM EDTA buffer (pH 8.0). The mixture was spinned at 25 rpm overnight at room temperature. Subsequently, the supernatant was filtered through a 0.5 µm filter into an Amicon® Ultra-4 Centrifugal Filter, 10 kDa MWCO, using a syringe. The solution was concentrated to 250 µL and subjected to ethanol precipitation as previously described. The final dried pellet was redissolved in 30 µL of dd$H_2O$. The RNA concentration was measured using a NanoDrop and found to be approximately 1-10 ng/µL.

**Deconvoluting loop-closing ligation and nicked duplex ligation**

We used four strand loop-closing ligation reactions to deconvolute loop closing ligation from nicked duplex ligation (Figure 2 and Figure S6). 10 µL reaction mixture, containing phosphate acceptor strand A (5 nM or 250 nM, X = A, C, G, or U in strand A, see Table S1), pre-activated phosphate donor strand a ( 6 nM or 300 nM, X = A, C, G, or U in strand a, see Table S1), phosphate acceptor strand B (5 nM or 250 nM), pre-activated phosphate donor strand b ( 6 nM or 300 nM), Blocker-1 (12 nM or 600 nM), Blocker-2 (12 nM or 600 nM), $MgCl_2$ 50 mM, HEPES 100 mM (pH 8.0) and *N*-methylimidazole (50 mM, pH 8.0, added at last) was incubated for 20 hours at 23 °C. 1 µL of each reaction mixture was quenched by addition to 9 µL of stop solution (50 mM EDTA pH 8.0 in 90 % formamide). 2 µL of the quenched solution was analysed by 10 % analytic PAGE gel. Observed yields were quantified according to the relative amounts of FAM-labelled oligomers by gel imaging.

For sequencing, the reactions at 5 nM were scaled up to 10 mL with similar condition.

**Preparing the multiplexed DNA library for deep sequencing**

For sequencing library preparation see Figure S1, steps 1 to 5.

**Step 1.** The pre-activated 5'-phosphorimidazolide RNA, Im-p-RNA, was synthesized as described as above.

**Step 2.** Large-scale loop-closing ligation reactions were conducted, and the ligated products were purified by RNA preparative PAGE gel, as detailed above.

**Step 3.** A 50 µL aqueous solution containing approximately 100 ng of gel-purified RNA (ligation product), 5 µM RT primer (Table S1), 1x ProtoScript II Buffer, 5 mM dNTPs, 10 mM $MgCl_2$, 10 mM DTT, and ProtoScript II Reverse Transcriptase (final enzyme concentration of 8 U/µL) (NEB: M0368L) was incubated at 42°C for 12 hours. Then, the solution was heated to 80°C for 5 minutes and then maintained at 4°C. Subsequently, 8 µL of 1M NaOH was added, and the mixture was heated at 90°C for 5 minutes, followed by the addition of 8 µL of 1M HCl. The resulting solution was processed

through a Zymo Oligo Clean & Concentrate spin column according to the manufacturer's instructions. The cDNA was eluted in 20 µL of TE buffer (pH 7) and stored at 4°C. The cDNA stock concentration, typically in the low ng/µL range, was measured by NanoDrop.

**Step 4.** A 25 µL solution, consisting of 100 ng cDNA, 0.2 mM dNTP, 0.5 µM each of Primer_P7 (NEBNext Index primer for Illumina, Table S1) and Primer_P5 (NEBNext SR primer for Illumina, Table S1), 1x Q5 reaction buffer, and Q5 Hot Start High-Fidelity DNA Polymerase (0.8 U total) was subjected to 6 PCR cycles with a 15-second extension step at 62°C. After amplification, 5 µL of Gel Loading Dye, Purple (6X) (NEB: B7024S) was added, and the entire volume was run on a preparative 1.4% w/v agarose gel in 1X TAE. Gel solution was prepared as 1.4% w/v agarose in 1X TAE (Tris Acetate-EDTA) and SYBR Safe DNA Gel Stain (5 µL in 50 mL gel solution, Invitrogen: S33102). The gel, cast as 10 × 5 cm with large wells capable of holding 50 µL of sample, was run at 100 V for 90 minutes at constant voltage. Target bands were visualized using a blue light transilluminator, excised, and purified using a Quantum Prep Freeze'N Squeeze spin column (Bio-Rad). The eluate was further purified using Agencourt AMPure XP magnetic beads with a 1.7:1 bead to sample volume ratio. The purified target material was eluted in 20 µL TE (pH 7) and prepared for sequencing.

**Step 5.** Samples were validated by TapeStation (Agilent) and qPCR before sequencing by Illumina MiSeq.

**Sequencing data analysis and verification of sequencing results**

**Step 6 (Figure S1).** The raw sequence data were processed using a custom Python program, tailored specifically for this study. The final high-quality sequencing reads were derived through stringent trimming criteria, ensuring robustness in downstream analyses. The criteria employed were as follows: 1) the quality score of both R1 and R2 reads must be at least 30; 2) fixed regions within R1 and R2 must exactly match the

expected sequences; 3) R1 and R2 sequences should be reverse complements of each other. The outcomes of the trimming process are detailed in Tables S2, S3, and S4.

**Step 7 (Figure S1).** Test oligonucleotides are designated as Test-NNNN in Table S1. A 10 µL reaction mixture, consisting of 1 µM preactivated phosphate donor strand Test-donor (Im-p-GCACGAGU) and one of the phosphate acceptor strands, Test-NNNN (1.5 µM, sequences 5'-FAM-UUACUCGUGCNNNN-3' or 5'-FAM-AUCGUGCNNNN-3' as listed in Table S1), along with 50 mM $MgCl_2$, 100 mM HEPES (pH 8.0), and 50 mM *N*-methylimidazole (added last), was incubated at 23°C for 10 hours. Each reaction mixture was subsequently quenched by adding 1 µL to 9 µL of stop solution (50 mM EDTA, pH 8.0, in 90% formamide). 2 µL of each quenched sample was analyzed on a 10% analytical denatured PAGE gel. Observed yields were quantified based on the relative amounts of FAM-labelled oligomers, as detected by gel imaging. The yields of the 34 tested overhang sequences were used to generate a ranking order, which was then compared with their ranking from the NGS data. Spearman's ranking correlation coefficient was calculated to be $\rho = 0.84$ (details see Appendix S1). Results are presented in Figure 4.

**Assembly of the full-length Hammerhead ribozyme via single loop-closing ligation**

A high-yielding sequence, UUCG, was engineered into the Hammerhead ribozyme to optimize the loop-closing ligation efficiency. The reaction was carried out in a 10 µL mixture containing 1 µM of 5'-FAM-CCUGUCUGAUGAGUCCUUCG-3' (HH-5'-half) and 2 µM of 5′-Im-p-GGACGAAACCGU-3' (Im-p-HH-3'-half, including both Im-p-RNA and p-RNA), $MgCl_2$ (either 5 mM or 50 mM), 100 mM HEPES buffer (pH 8.0), and 50 mM *N*-methylimidazole (added last). The reaction was incubated at 23°C. At specified time points, 1 µL aliquots were taken and quenched in 9 µL of stop solution (50 mM EDTA, pH 8.0, in 90% formamide). 2 µL of each quenched sample was analyzed on a 20% denatured PAGE gel. Observed yields were quantified by comparing the relative amounts of FAM-labelled oligomers through gel imaging.

**Assembly of the full-length Flexizyme via two simultaneous loop-closing ligations**

A high-yielding sequence, UUCG, was integrated into the two hairpin loop regions of the Flexizyme to optimize ligation efficiency. The reaction was carried out in a 10 μL mixture containing 1 μM 5'-FAM-GGACCUUCG-3' (Flex1), 2 μM 5′-Im-p-GGUCCCGCAUCCCAUCUUCG-3' (Im-p-Flex2, including Im-p-RNA and p-RNA), and 5 μM 5′-Im-p-GAUGGUACAUGGCGUUAGGU-3' (Im-p-Flex3, including Im-p-RNA and p-RNA), 50 mM $MgCl_2$, 100 mM HEPES buffer (pH 8.0), and 50 mM *N*-methylimidazole (added last). The reaction was incubated at 23°C. At specified time points, 1 μL aliquots were taken and quenched in 9 μL of stop solution (50 mM EDTA, pH 8.0, in 90% formamide). 2 μL of each quenched sample was analyzed on a 20% denatured PAGE gel. Observed yields were quantified by comparing the relative amounts of FAM-labelled oligomers through gel imaging.

## Supplementary Figures

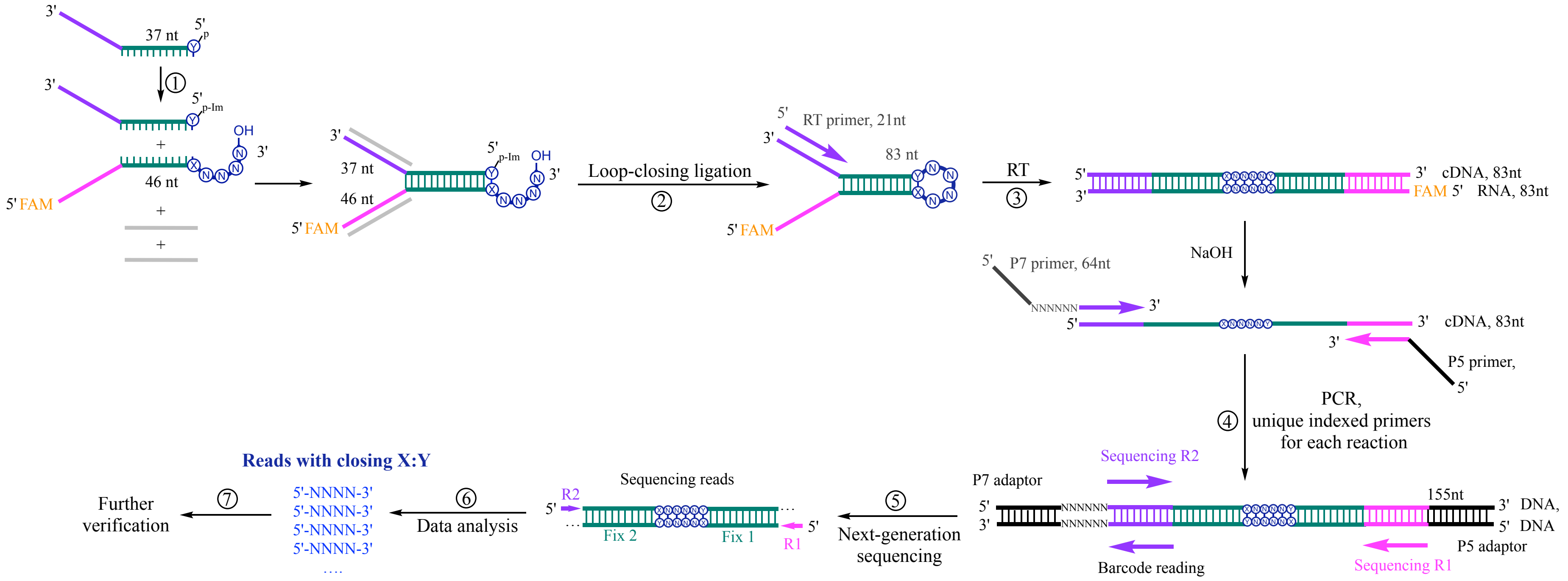


**Figure S1. Outline of the sequencing workflow used in this work.** Step 1, Chemical synthesis of Imp-RNA from p-RNA. Step 2, Ligated products from large-scale loop-closing ligation purified by prep-PAGE gel. Step 3, cDNA preparation through reverse transcription followed by NaOH treatment. Step 4, Indexed PCR performed using indexed primers. Step 5, Pooling of the indexed DNA library for next-generation-sequencing. Step 6, Data analysis using custom Python program. Step 7, Verification of sequencing results by additional testing.

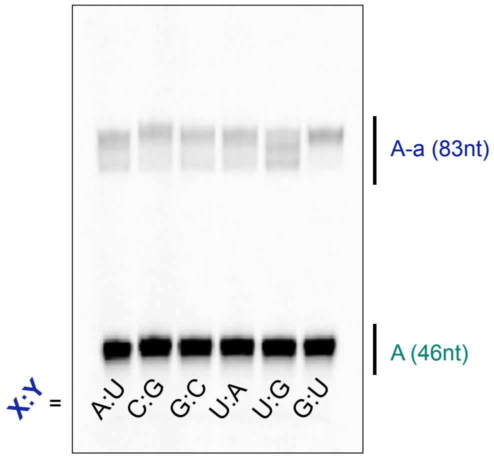


**Figure S2. Representative denatured RNA PAGE gel.** This figure displays the results for reactions with 250 nM duplex A:a, as shown in Figure 2. Uncropped image can be found in the end of this document.

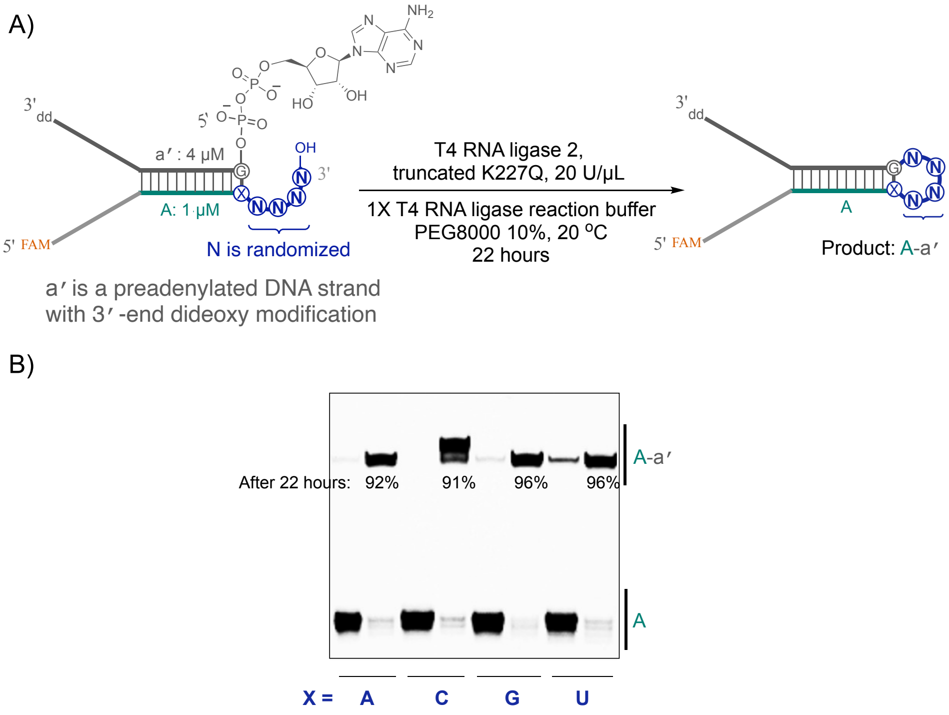


**Figure S3. Loop-closing ligation by T4 RNA ligase.** A) Reaction scheme and condition. The 3'-end nucleoside of strand a' features a dideoxy modification to prevent undesired ligation reaction. Possible splint ligation product is omitted in the scheme. B) Representative denatured RNA PAGE gel and ligation yields. Yields at 1 min and 22

hours are shown. The ligated products were purified and subjected to the sequencing workflow, from step 3 to step 7 as outlined in Figure S1. Uncropped image can be found in the end of this document.

A)

B)

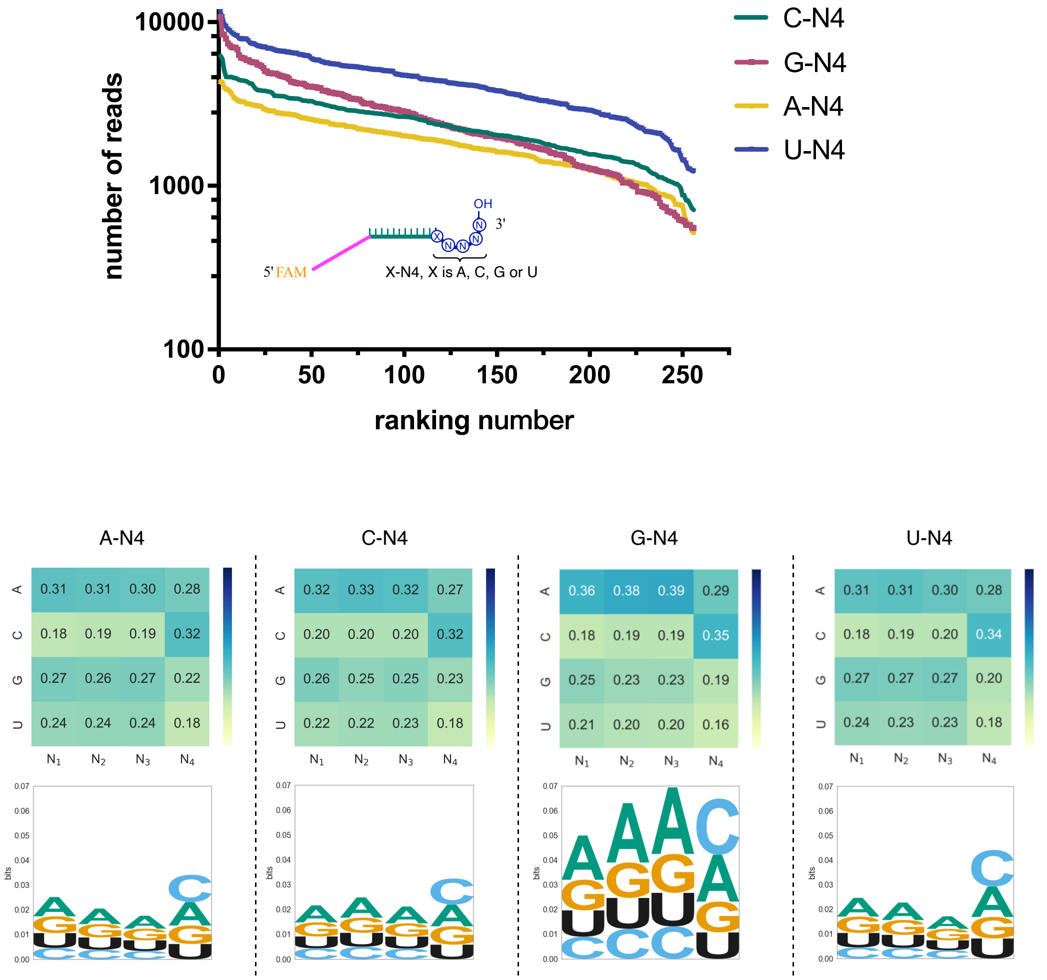


**Figure S4. Distribution of randomized NNNN overhang sequences in the synthesized oligonucleotide.** A) Number of sequencing reads relative to their ranking number, derived from the products of reactions shown in Figure S3. B) Heat maps and sequence consensus logos of the four NNNN overhangs display the distribution of the four nucleotides at each position within the starting materials (information content from 0 to 2 bits). This visual representation highlights the imbalance among nucleotides at each position.

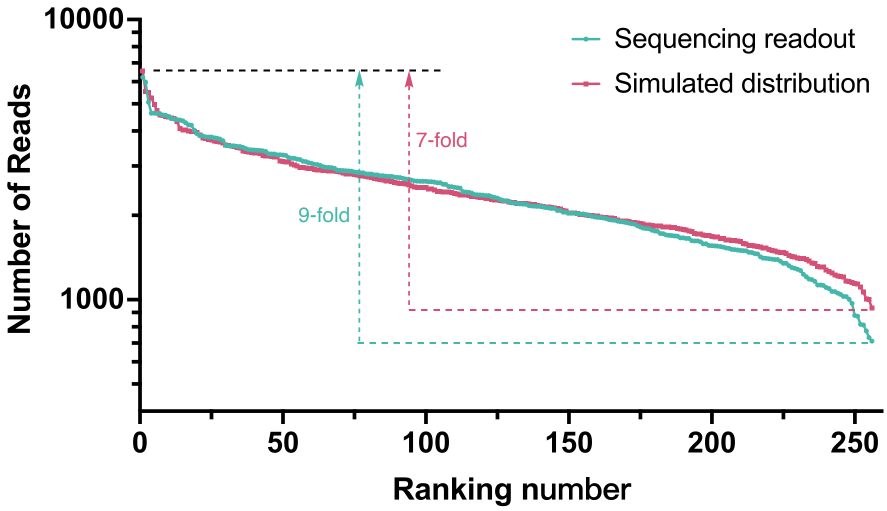


**Figure S5. The variation in abundance of the randomized NNNN results from the biased base-composition at each position.** The base compositions obtained for each position, as depicted in the heatmap of C-N4 in Figure S4, effectively simulate the uneven distribution observed in the NGS results for C-N4.

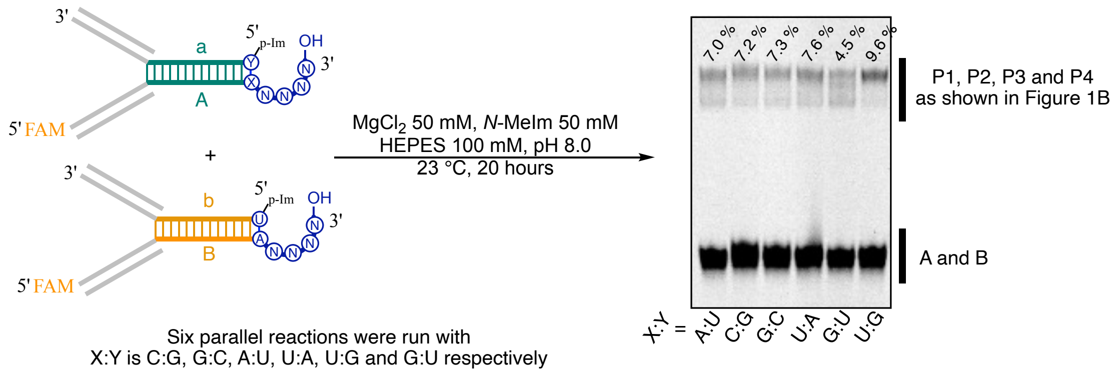


**Figure S6. Deconvolution of splint ligation and loop-closing ligation using four-strand reactions.** Reactions were performed using concentrations of 5 nM or 250 nM for each duplex A:a and B:b. Displayed is a representative gel result from reactions containing 250 nM of each duplex A:a and B:b. Uncropped image can be found in the end of this document.

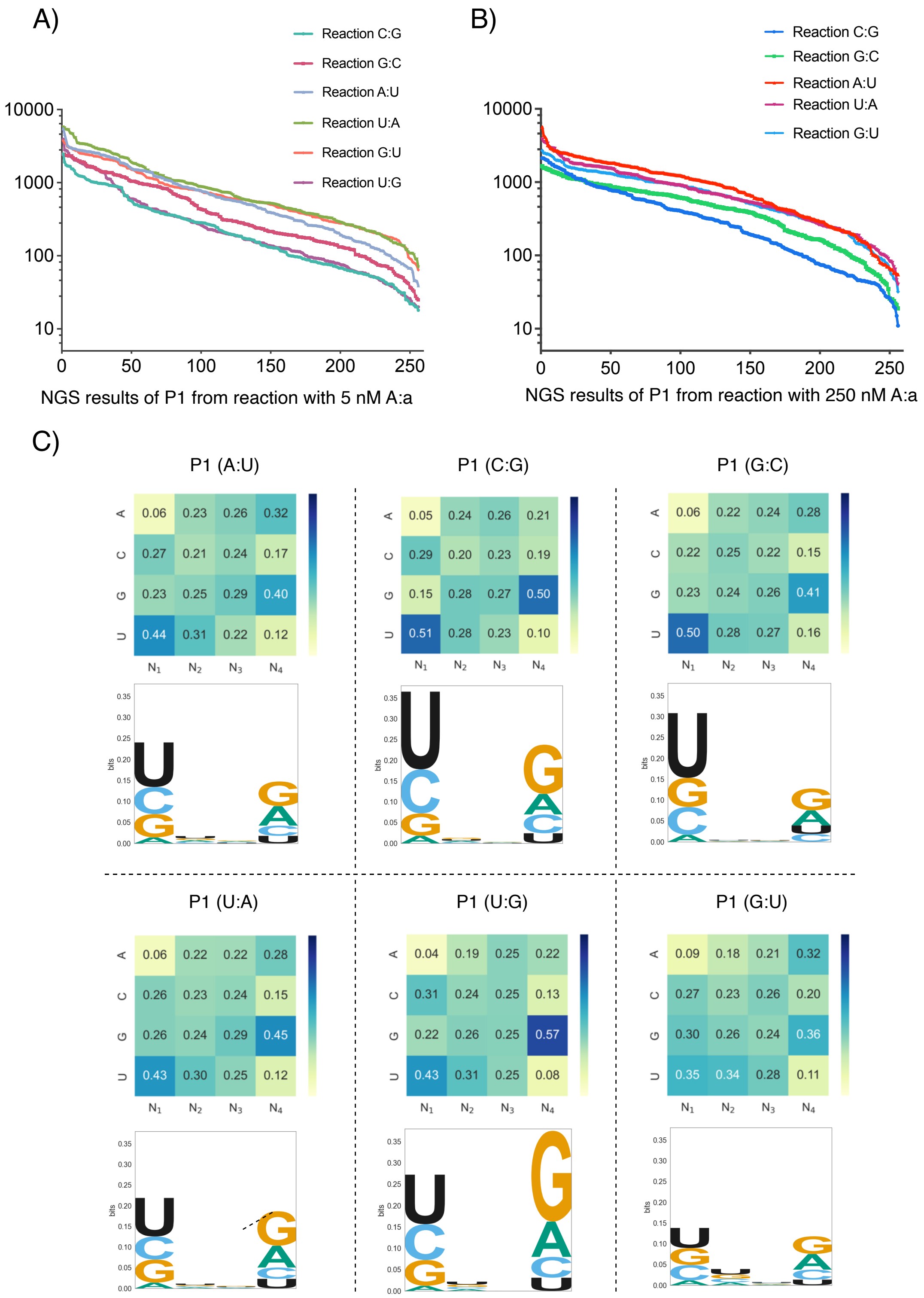


**Figure S7. Profiles of product P1(A-a) from loop-closing ligation.** A) and B) Product P1 displays logarithmic decrease of sequencing reads relative to their ranking number. C) Heat maps and sequence consensus logos for P1 (information content from 0 to 2 bits), derived from the six reactions at 5 nM featuring different closing base-pairs, show distinct patterns compared to the starting distribution presented in Figure S4.

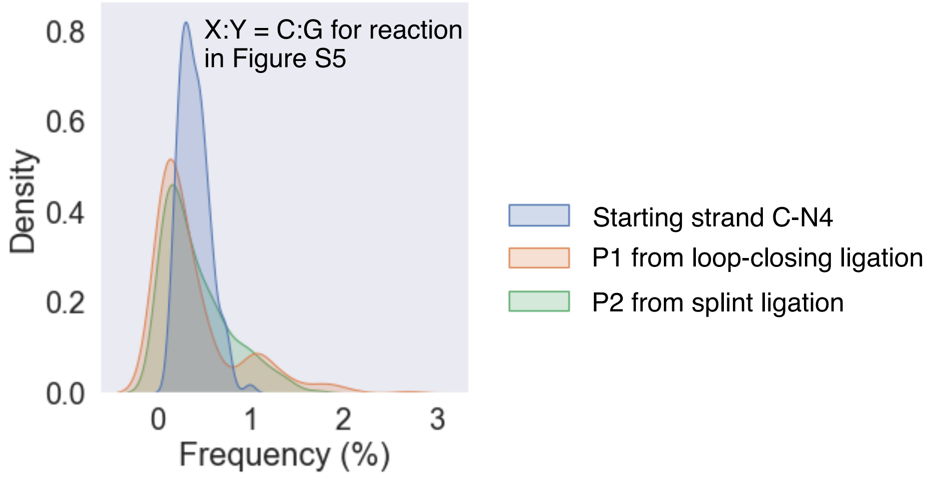


**Figure S8. Changes of probability distributions from starting strand C-N4 to products P1 (loop-closing ligation) and P2 (splint ligation).** Representative results of reaction C:G at 5 nM as shown in Figure S6. The probability distribution of P1, resulting from loop-closing ligation, is significantly more varied and rugged compared to that of P2 or the starting strand.

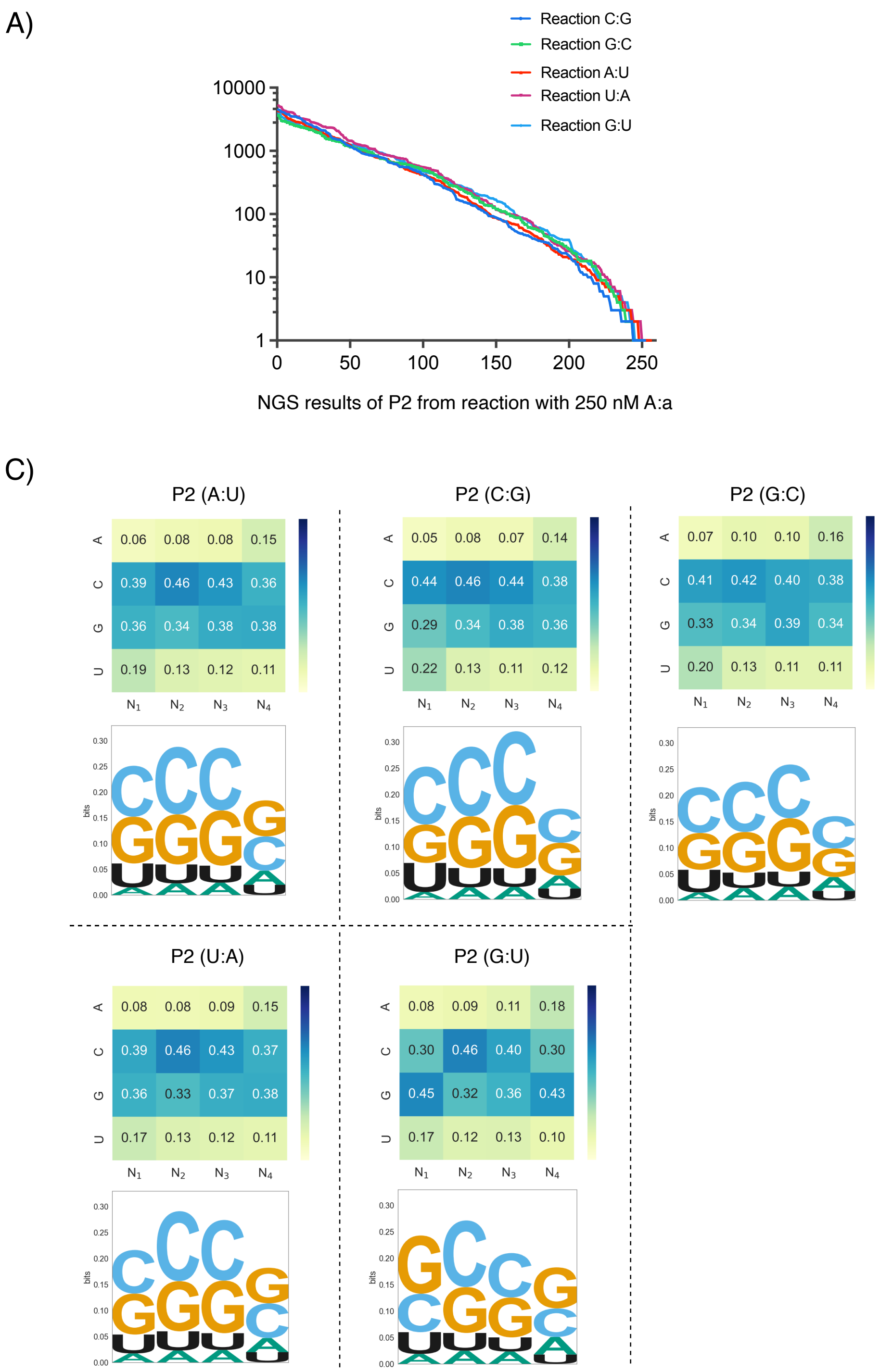


**Figure S9. Profiles of the product P2 from splint ligation (reactions in Figure S5).** A) Product P2 exhibits a logarithmic decrease in sequencing reads relative to their ranking number. B) Heat maps and sequence consensus logos for P2 (information content from 0 to 2 bits), derived from the six reactions at 250 nM featuring different

closing base-pairs, show patterns that are distinct from those of loop-closing ligation, as demonstrated in Figure S6.

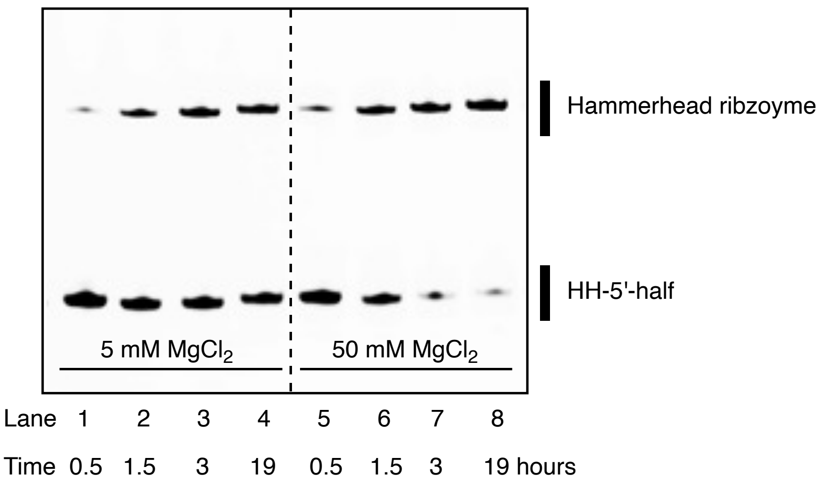


**Figure S10. Representative gel of self-assembling the Hammerhead ribozyme via one loop-closing ligation (See also Figure 5).** Uncropped image can be found in the end of this document.

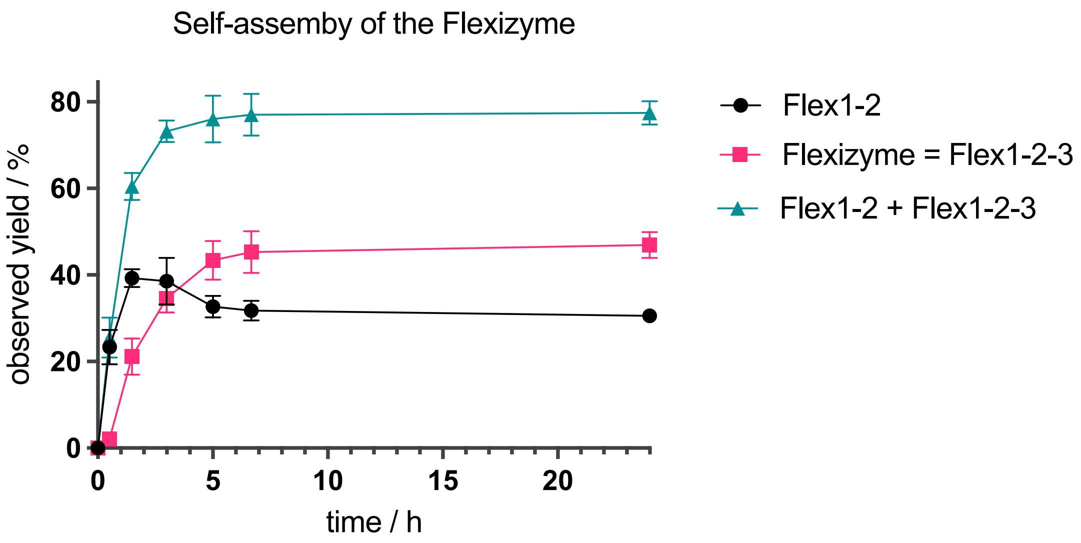


**Figure S11. Time course of the self-assembly of the Flexizyme by two simultaneous loop-closing ligations (See also Figure 5).** The assembly process resulted in a 77% observed yield for one loop (Flex1-2 + Flex1-2-3), while the closure of the other loop was inferred to occur with a 58% yield, calculated as the ratio (Flex1-2-3)/(Flex1-2 + Flex1-2-3).

X:Y = C:G

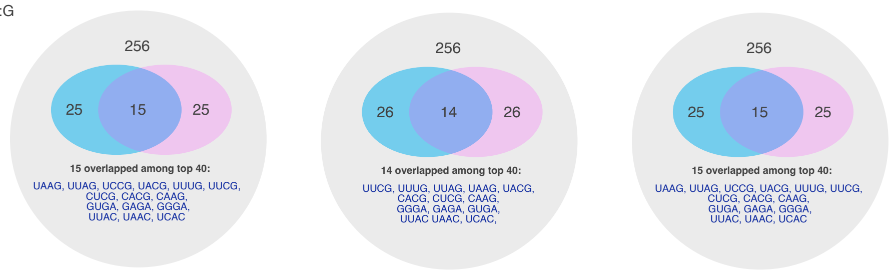


X:Y = G:C

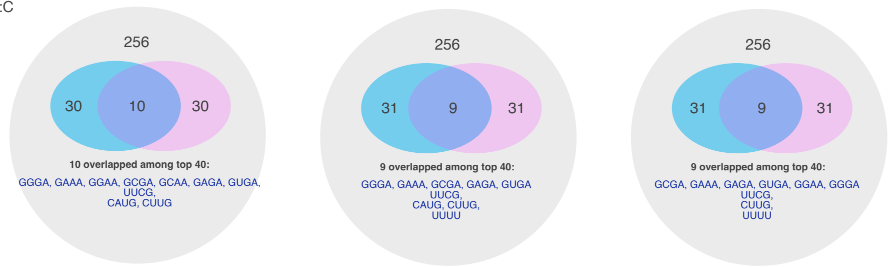


X:Y = A:U

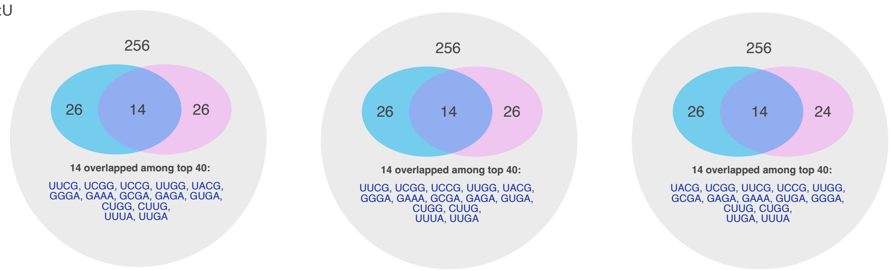


X:Y = U:A

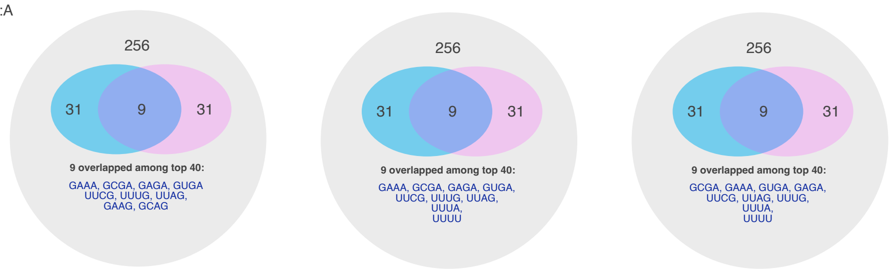


X:Y = U:G

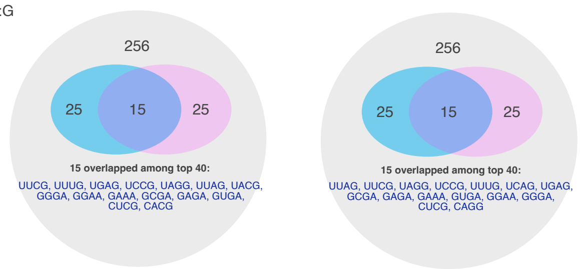


X:Y = G:U

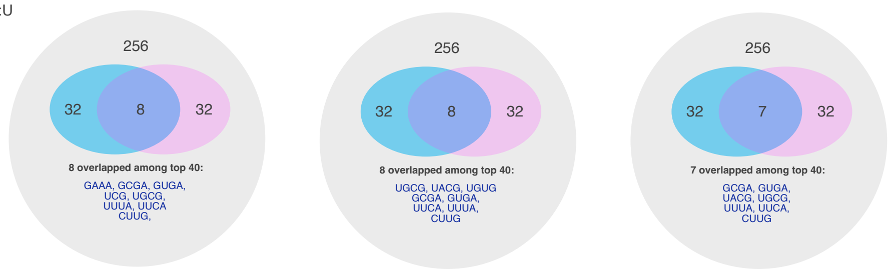

**Figure S12. Overlap of top 40 sequences from loop-closing ligation with top 40 sequences in biological tetraloops.** The data includes results from three independent experiments for each of the six reactions, with the exception of the U:G reaction.

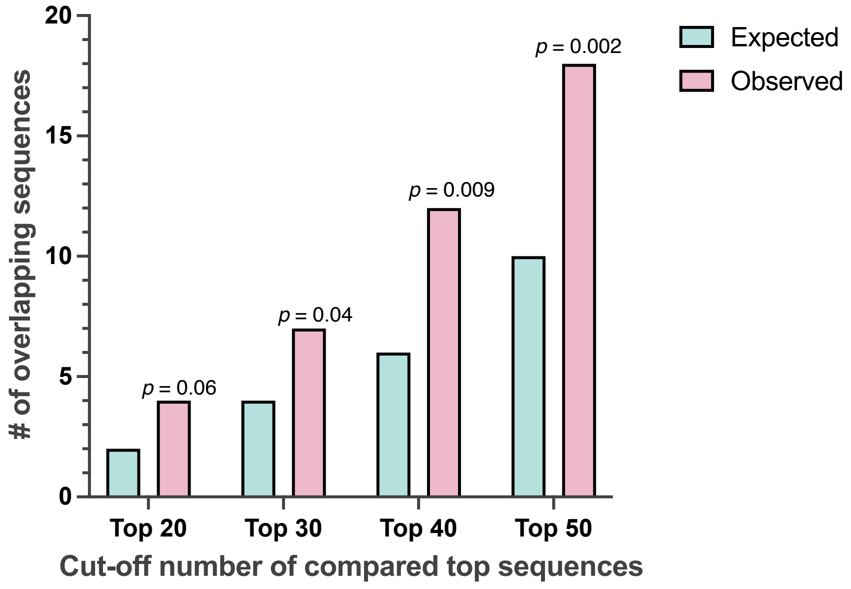


**Figure S13. Over-representation of overlapping sequences across different cut-off number.** Hypergeometric p-value indicating over-representation is shown for each cut-off number. Regardless of the cut-off numbers used (20, 30, 40, or 50 respectively) to define the top sequences, across all comparisons, the overlapping sequences showed approximately a 2-fold over-representation compared to what would be expected by chance.

X:Y = C:G

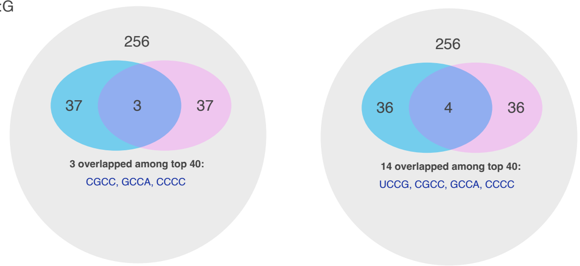


X:Y = G:C

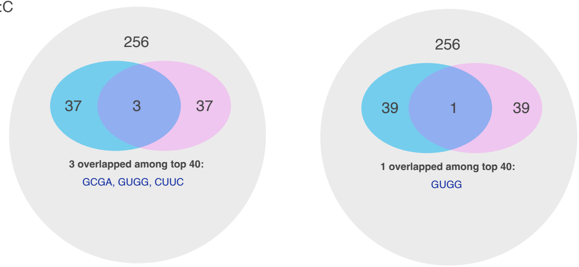


X:Y = A:U

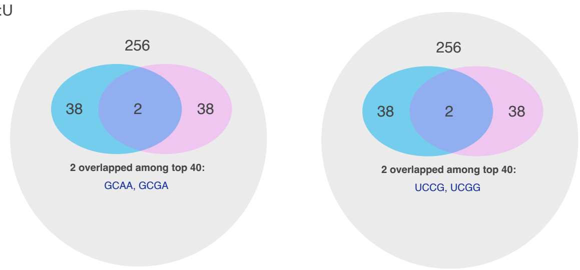


X:Y = U:A

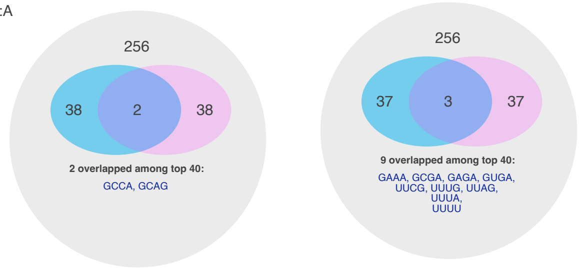


X:Y = U:G

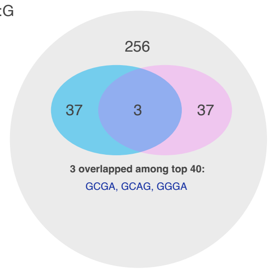


X:Y = G:U

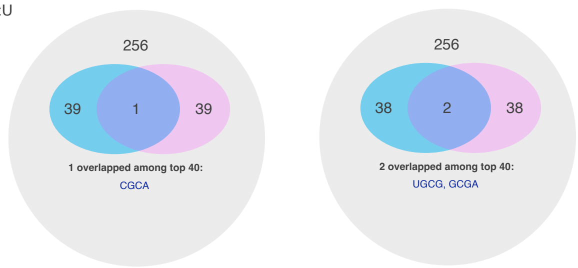

**Figure S14. Overlap of top 40 sequences from splint ligation with top 40 sequences in biological tetraloops.** Two independent experiments for each of the six reactions, except reaction U:G. The data includes results from two independent experiments for each of the six reactions, with the exception of the U:G reaction.

**Table S1. RNA/DNA oligonucleotides used in this work.**

| Oligo name | Sequence information | Resource | Notes |
|---|---|---|---|
| A (A-N4) | 5'-FAM-GUUCAGAGUUCUACAGUCCGACGAUCUACAGUCAAGUCGUGANNNN-3' | In-house | RNA, 46nt, N is randomized |
| A (C-N4) | 5'-FAM-GUUCAGAGUUCUACAGUCCGACGAUCUCGAGUCAAGUCGUGCNNNN-3' | In-house | RNA, 46nt, N is randomized |
| A (G-N4) | 5'-FAM-GUUCAGAGUUCUACAGUCCGACGAUCACAAGUCAAGUCGUGGNNNN-3' | In-house | RNA, 46nt, N is randomized |
| A (U-N4) | 5'-FAM-GUUCAGAGUUCUACAGUCCGACGAUCAGUAGUCAAGUCGUGUNNNN-3' | In-house | RNA, 46nt, N is randomized |
| a (A-N4) | 5'-p-ACACGACUUGACACAUAGAUCGGAAGACCACACGUCU-3' | In-house | RNA, 37nt |
| a (C-N4) | 5'-p-CCACGACUUGACAUCAAGAUCGGAAGACCACACGUCU-3' | In-house | RNA, 37nt |
| a (G-N4) | 5'-p-GCACGACUUGACAAUAAGAUCGGAAGACCACACGUCU-3' | In-house | RNA, 37nt |
| a (U-N4) | 5'-p-UCACGACUUGACCUACAGAUCGGAAGACCACACGUCU-3' | In-house | RNA, 37nt |
| B | 5'-FAM-GUUCAGAGUUCUACAGUCCGACGAUCUAUACGUAAGCAGCGANNNN-3' | In-house | RNA, 46nt, N is randomized |
| b | 5'-p-UCGCUGCUUACGAUAAAGAUCGGAAGACCACACGUCU-3' | In-house | RNA, 37nt |
| a' | 5'-p-GCACGACTTGACGCAAAAGATCGGAAGACCACACGTCT(dd)-3' | IDT | DNA, 38nt, dideoxy modification |
| Blocker-1 | 5'-GATCGTCGGACTGTAGAACTCTGAAC-3' | IDT | DNA, 26nt |
| Blocker-2 | 5'-AGACGTGTGGTCTTCCGATCT-3' | IDT | DNA, 21nt |

| | | | |
|---|---|---|---|
| RT primer | 5'-AGACGTGTGGTCTTCCGATCT-3' | IDT | DNA, 21nt, same as blocker-2 |
| Primer_P5 | 5'-AATGATACGGCGACCACCGAGATCTACACGTTCAGAGTTCTACAGTCCG-s-A-3' | IDT | DNA, 50nt, For indexed PCR, -s- indicates phosphorothioate bond |
| Primer_P7 | 5’-CAAGCAGAAGACGGCATACGAGATNNNNNNGTGACTGGAGTTCAGACGTGTG CTCTTCCGATC-s-T-3’ | NEB | DNA, 64nt, For indexed PCR, -s- indicates phosphorothioate bond, NEB #E7335S/L |
| Test-donor | 5'-p-GCACGAGU-3' | In-house | RNA, 8nt |
| Test-GCGA | 5'-FAM-UUACUCGUGCGCGA-3' | IDT | RNA, 14nt |
| Test-GAAA | 5'-FAM-UUACUCGUGCGAAA-3' | IDT | RNA, 14nt, 1st tset GAAA |
| Test-UCGC | 5'-FAM-UUACUCGUGCUCGC-3' | IDT | RNA, 14nt |
| Test-UGGC | 5'-FAM-UUACUCGUGCUGGC-3' | IDT | RNA, 14nt |
| Test-CCUG | 5'-FAM-UUACUCGUGCCCUG-3' | IDT | RNA, 14nt |
| Test-CAAG | 5'-FAM-UUACUCGUGCCAAG-3' | IDT | RNA, 14nt |
| Test-CCAG | 5'-FAM-UUACUCGUGCCCAG-3' | IDT | RNA, 14nt |
| Test-AAAU | 5'-FAM-UUACUCGUGCAAAU-3' | IDT | RNA, 14nt |

| | | | |
|---|---|---|---|
| Test-AAGC | 5'-FAM-UUACUCGUGCAAGC-3' | IDT | RNA, 14nt |
| Test-GCCC | 5'-FAM-UUACUCGUGCGCCC-3' | IDT | RNA, 14nt |
| Test-GACU | 5'-FAM-UUACUCGUGCGACU-3' | IDT | RNA, 14nt |
| Test-AACC | 5'-FAM-UUACUCGUGCAACC-3' | IDT | RNA, 14nt |
| Test-UCCA | 5'-FAM-UUACUCGUGCUCCA-3' | IDT | RNA, 14nt |
| Test-UUGG | 5'-FAM-UUACUCGUGCUUGG-3' | IDT | RNA, 14nt |
| Test-UGGU | 5'-FAM-UUACUCGUGCUGGU-3' | IDT | RNA, 14nt |
| Test-UUCG | 5'-FAM-UUACUCGUGCUUCG-3' | IDT | RNA, 14nt |
| Test-UACG | 5'-FAM-UUACUCGUGCUACG-3' | IDT | RNA, 14nt |
| Test-UUUA | 5'-FAM-UUACUCGUGCUUUA-3' | IDT | RNA, 14nt |
| Test-UAAC | 5'-FAM-UUACUCGUGCUAAC-3' | IDT | RNA, 14nt |
| Test-UUAG | 5'-FAM-UUACUCGUGCUUAG-3' | IDT | RNA, 14nt |
| Test-GAAA | 5'-FAM-UUACUCGUGCGAAA-3' | IDT | RNA, 14nt, 2nd test GAAA |
| Test-UGGG | 5'-FAM-UUACUCGUGCUGGG-3 | IDT | RNA, 14nt |
| Test-UCGG | 5'-FAM-UUACUCGUGCUCGG-3 | IDT | RNA, 14nt |
| Test-UGCG | 5'-FAM-UUACUCGUGCUGCG-3 | IDT | RNA, 14nt |

| | | | |
|---|---|---|---|
| Test-UCUG | 5'-FAM-UUACUCGUGCUCUG-3 | IDT | RNA, 14nt |
| Test-UCCG | 5'-FAM-UUACUCGUGCUCCG-3 | IDT | RNA, 14nt, 1st test UCCG |
| Test-GGGA | 5'-FAM-AUCGUGCGGGA-3' | IDT | RNA, 11nt |
| Test-GAGA | 5'-FAM-AUCGUGCGAGA-3' | IDT | RNA, 11nt |
| Test-GUGA | 5'-FAM-AUCGUGCGUGA-3' | IDT | RNA, 11nt |
| Test-CAGG | 5'-FAM-AUCGUGCCAGG-3' | IDT | RNA, 11nt |
| Test-CUGG | 5'-FAM-AUCGUGCCUGG-3' | IDT | RNA, 11nt |
| Test-CUCG | 5'-FAM-AUCGUGCCUCG-3' | IDT | RNA, 11nt |
| Test-UUGC | 5'-FAM-AUCGUGCUUGC-3' | IDT | RNA, 11nt |
| Test-UUUG | 5'-FAM-AUCGUGCUUUG-3' | IDT | RNA, 11nt |
| Test-UCCG | 5'-FAM-AUCGUGCUCCG-3' | IDT | RNA, 11nt, 2nd test UCCG |
| Test-UUCA | 5'-FAM-AUCGUGCUUCA-3' | IDT | RNA, 11nt |
| HH-5'-half | 5'-FAM-CCUGUCUGAUGAGUCCUUCG-3' | IDT | RNA, 20nt, for assembly of the Hammerhead ribozyme |
| HH-3'-half | 5'-p-GGACGAAACCGU-3' | IDT | RNA, 12nt, for assembly of the Hammerhead ribozyme |

| | | | |
|---|---|---|---|
| Flex1 | 5'-FAM-GGACCUUCG-3' | In-house | RNA, 9nt,<br>for assembly of the Flexizyme |
| Flex2 | 5'-p-GGUCCCGCAUCCCAUCUUCG-3' | In-house | RNA, 20nt,<br>for assembly of the Flexizyme |
| Flex3 | 5'-p-GAUGGUACAUGGCGUUAGGU-3' | In-house | RNA, 20nt,<br>for assembly of the Flexizyme |

'

**Table S2. Prior distribution of randomized NNNN overhang by RNA deep sequencing.** Frequency range is calculated based on the difference in reads between the most and least frequent sequences.

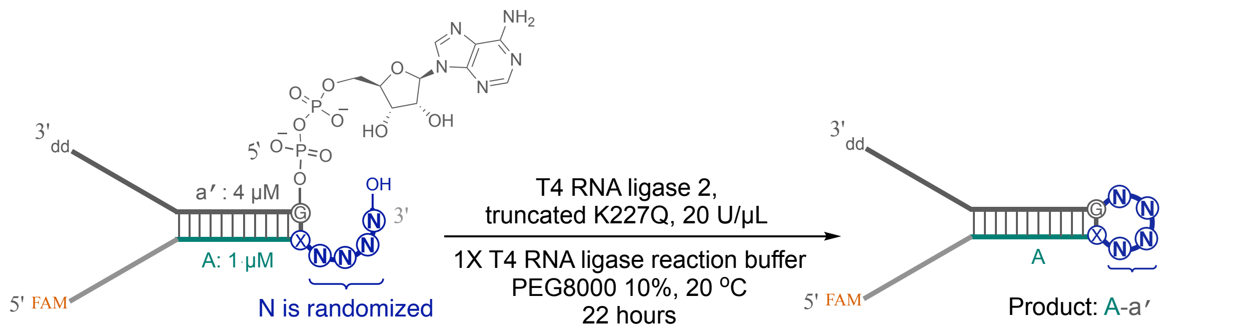


| Quality control by trimming the sequencing data | | | | | |
|---|---|---|---|---|---|
| X-N4 | Seq_reads total | Seq_reads after filter1 | Seq_reads after filter2 | Final Seq_reads after filter3 | Final seq_reads remained |
| C-N4 | 1163816 | 917529 | 618461 | 618370 | 53 % |
| G-N4 | 1663957 | 1281352 | 693581 | 693451 | 42 % |
| A-N4 | 961726 | 730498 | 485850 | 485789 | 51 % |
| U-N4 | 2325663 | 1804315 | 1131945 | 1131645 | 49 % |
| Average | | | | | 51 % |

Detailed sequencing results ranked by their decreasing reads

| rank order | X=C NNNN | Reads | $\alpha_i$ | X=G NNNN | Reads | $\alpha_i$ | X=A NNNN | Reads | $\alpha_i$ | X=U NNNN | Reads | $\alpha_i$ |
|---|---|---|---|---|---|---|---|---|---|---|---|---|
| 1 | AAGC | 6223 | 2.586 | AAAC | 10741 | 3.968 | AAGC | 4356 | 2.304 | AAGC | 11563 | 2.611 |
| 2 | AAAC | 5979 | 2.483 | UAAC | 8416 | 3.098 | AGGC | 4316 | 2.278 | AGGC | 10736 | 2.432 |
| 3 | AGGC | 5068 | 2.099 | AAGC | 8384 | 3.098 | GUAC | 4012 | 2.125 | AAAC | 9601 | 2.176 |
| 4 | AUAC | 4633 | 1.920 | AAAA | 7955 | 2.944 | AAAC | 3919 | 2.074 | UAGC | 9402 | 2.125 |
| 5 | GUAC | 4627 | 1.920 | GAAC | 7403 | 2.739 | UAGC | 3895 | 2.048 | GUAC | 9018 | 2.048 |
| 6 | AGAC | 4623 | 1.920 | AGAC | 7185 | 2.662 | AGUA | 3809 | 1.997 | UAAC | 8859 | 1.997 |
| 7 | UAAC | 4618 | 1.920 | AAAG | 7017 | 2.586 | UAAC | 3669 | 1.946 | AUAC | 8593 | 1.946 |
| 8 | AAAG | 4562 | 1.894 | AUAC | 6999 | 2.586 | AUAC | 3489 | 1.843 | AGUA | 8419 | 1.894 |
| 9 | CAAC | 4556 | 1.894 | CAAC | 6988 | 2.586 | AUGC | 3434 | 1.818 | GAGC | 8291 | 1.869 |
| 10 | AAUC | 4504 | 1.869 | ACAC | 6827 | 2.509 | AGAC | 3367 | 1.766 | AGAC | 8238 | 1.869 |
| 11 | CAGC | 4430 | 1.843 | UAAA | 6268 | 2.304 | GAGC | 3309 | 1.741 | GGGC | 7946 | 1.792 |
| 12 | GCAC | 4412 | 1.818 | GUAC | 6094 | 2.253 | GAAC | 3290 | 1.741 | AAUC | 7892 | 1.792 |
| 13 | GAAC | 4395 | 1.818 | AAUC | 6009 | 2.227 | GGUA | 3276 | 1.715 | CAGC | 7891 | 1.792 |
| 14 | ACAC | 4377 | 1.818 | GCAC | 6003 | 2.227 | AAUC | 3273 | 1.715 | AUGC | 7883 | 1.792 |
| 15 | GAGC | 4351 | 1.792 | AACA | 5875 | 2.176 | AAAG | 3198 | 1.690 | GAAC | 7853 | 1.766 |
| 16 | AAGG | 4280 | 1.766 | AAGA | 5852 | 2.150 | CAGC | 3176 | 1.664 | GGAC | 7682 | 1.741 |
| 17 | UAGC | 4213 | 1.741 | UAGC | 5742 | 2.125 | GUAA | 3168 | 1.664 | GGUA | 7439 | 1.690 |
| 18 | AAAA | 4195 | 1.741 | AGGC | 5732 | 2.125 | GGGC | 3140 | 1.664 | GCAC | 7377 | 1.664 |
| 19 | UAAG | 4030 | 1.664 | AACC | 5676 | 2.099 | AAGA | 3133 | 1.638 | ACAC | 7344 | 1.664 |

| | | | | | | | | | | | | |
|---|---|---|---|---|---|---|---|---|---|---|---|---|
| 20 | AGUA | 3911 | 1.613 | AAAU | 5668 | 2.099 | AGUC | 3105 | 1.638 | CAAC | 7243 | 1.638 |
| 21 | AACA | 3847 | 1.587 | AUAA | 5528 | 2.048 | AAAA | 3098 | 1.638 | AGUC | 7147 | 1.613 |
| 22 | AAUA | 3843 | 1.587 | GAAA | 5471 | 2.022 | AAGG | 3080 | 1.613 | AAAA | 7125 | 1.613 |
| 23 | AUAG | 3813 | 1.587 | AAUA | 5376 | 1.997 | AAUA | 3071 | 1.613 | GUAA | 7046 | 1.587 |
| 24 | AUGC | 3808 | 1.587 | AGAA | 5231 | 1.920 | UGGC | 2968 | 1.562 | AGCC | 7044 | 1.587 |
| 25 | AAGA | 3807 | 1.587 | UAAG | 5028 | 1.869 | GGAC | 2959 | 1.562 | UGGC | 7022 | 1.587 |
| 26 | AACC | 3783 | 1.562 | GAGC | 4984 | 1.843 | AUAA | 2919 | 1.536 | AAGA | 6901 | 1.562 |
| 27 | AUAA | 3767 | 1.562 | CAAA | 4893 | 1.818 | UAUC | 2894 | 1.536 | AAGG | 6898 | 1.562 |
| 28 | GUAA | 3744 | 1.562 | GGAC | 4891 | 1.818 | UAAA | 2887 | 1.510 | UAAA | 6886 | 1.562 |
| 29 | GGAC | 3693 | 1.536 | GAAG | 4847 | 1.792 | UGUA | 2885 | 1.510 | UAUC | 6820 | 1.536 |
| 30 | AGUC | 3549 | 1.459 | AAGG | 4843 | 1.792 | UUAC | 2871 | 1.510 | UAUA | 6761 | 1.536 |
| 31 | GGGC | 3544 | 1.459 | CAGC | 4834 | 1.792 | ACAC | 2822 | 1.485 | UAGA | 6673 | 1.510 |
| 32 | GCAA | 3542 | 1.459 | ACAA | 4787 | 1.766 | GCAC | 2809 | 1.485 | AGCA | 6667 | 1.510 |
| 33 | GAAA | 3539 | 1.459 | AGUA | 4718 | 1.741 | UAGA | 2786 | 1.459 | AACA | 6636 | 1.510 |
| 34 | GUAG | 3525 | 1.459 | AUAG | 4681 | 1.741 | UAUA | 2785 | 1.459 | AUAA | 6607 | 1.485 |
| 35 | GAAG | 3521 | 1.459 | GUAA | 4637 | 1.715 | AGCC | 2774 | 1.459 | AAUA | 6607 | 1.485 |
| 36 | AGCC | 3499 | 1.459 | GCAA | 4627 | 1.715 | AUAG | 2766 | 1.459 | ACGC | 6557 | 1.485 |
| 37 | AAUG | 3462 | 1.434 | AGCC | 4580 | 1.690 | AGCA | 2766 | 1.459 | UUAC | 6554 | 1.485 |
| 38 | ACGC | 3431 | 1.408 | UAGA | 4490 | 1.664 | GUGC | 2765 | 1.459 | GGUC | 6527 | 1.485 |
| 39 | GAUC | 3427 | 1.408 | UAUC | 4329 | 1.587 | AGAA | 2737 | 1.434 | AAAG | 6482 | 1.459 |
| 40 | UAUA | 3421 | 1.408 | AGCA | 4304 | 1.587 | AGGA | 2732 | 1.434 | AGGA | 6468 | 1.459 |
| 41 | AGCA | 3413 | 1.408 | CCAC | 4282 | 1.587 | CAAC | 2728 | 1.434 | GCGC | 6453 | 1.459 |
| 42 | UAAA | 3412 | 1.408 | UUAC | 4251 | 1.562 | GUAG | 2704 | 1.434 | UGUA | 6438 | 1.459 |
| 43 | AAAU | 3400 | 1.408 | ACAG | 4220 | 1.562 | AACA | 2682 | 1.408 | AAGU | 6336 | 1.434 |

| | | | | | | | | | | | | |
|---|---|---|---|---|---|---|---|---|---|---|---|---|
| 44 | AGAA | 3378 | 1.408 | AGAG | 4216 | 1.562 | GCGC | 2647 | 1.382 | GUGC | 6319 | 1.434 |
| 45 | GCGC | 3318 | 1.382 | UAAU | 4209 | 1.562 | AAUG | 2613 | 1.382 | AACC | 6302 | 1.434 |
| 46 | CAUC | 3313 | 1.382 | AUGC | 4185 | 1.536 | ACGC | 2612 | 1.382 | GAUC | 6246 | 1.408 |
| 47 | AGAG | 3311 | 1.382 | AGUC | 4108 | 1.510 | GGUC | 2605 | 1.382 | AGAA | 6231 | 1.408 |
| 48 | AAGU | 3289 | 1.357 | AAGU | 4053 | 1.485 | UAAG | 2581 | 1.357 | GGCC | 6223 | 1.408 |
| 49 | CUAC | 3288 | 1.357 | CAAG | 4053 | 1.485 | GAAG | 2571 | 1.357 | UACA | 6124 | 1.382 |
| 50 | UUAC | 3286 | 1.357 | AGGA | 4034 | 1.485 | GGCA | 2550 | 1.331 | UAAG | 5954 | 1.357 |
| 51 | GGUA | 3273 | 1.357 | UACA | 4029 | 1.485 | GGCC | 2550 | 1.331 | GCAA | 5895 | 1.331 |
| 52 | AGGA | 3218 | 1.331 | UAUA | 4011 | 1.485 | AAGU | 2526 | 1.331 | GGCA | 5862 | 1.331 |
| 53 | CCAC | 3198 | 1.331 | UGAC | 3989 | 1.485 | UAGG | 2514 | 1.331 | GAAA | 5852 | 1.331 |
| 54 | ACAG | 3197 | 1.331 | UCAC | 3957 | 1.459 | UGAC | 2509 | 1.331 | UGAC | 5836 | 1.331 |
| 55 | UAGA | 3176 | 1.306 | UACC | 3944 | 1.459 | AUUC | 2485 | 1.306 | CAUC | 5742 | 1.306 |
| 56 | CAAG | 3161 | 1.306 | GAUC | 3821 | 1.408 | GAAA | 2475 | 1.306 | GUAG | 5698 | 1.280 |
| 57 | UAUC | 3120 | 1.280 | AAUG | 3820 | 1.408 | AACC | 2473 | 1.306 | UUAA | 5639 | 1.280 |
| 58 | UACA | 3109 | 1.280 | ACGC | 3800 | 1.408 | AUUA | 2472 | 1.306 | UAGG | 5636 | 1.280 |
| 59 | UAGG | 3086 | 1.280 | GACC | 3755 | 1.382 | GAUA | 2460 | 1.306 | CGGC | 5612 | 1.280 |
| 60 | ACAA | 3067 | 1.280 | GACA | 3722 | 1.382 | UUAA | 2405 | 1.280 | GAUA | 5564 | 1.254 |
| 61 | GGCC | 3040 | 1.254 | GAAU | 3719 | 1.382 | AGAG | 2401 | 1.254 | AUUC | 5564 | 1.254 |
| 62 | GCCA | 3035 | 1.254 | AGAU | 3690 | 1.357 | AUGG | 2388 | 1.254 | AAAU | 5531 | 1.254 |
| 63 | GCAG | 3017 | 1.254 | GUAG | 3662 | 1.357 | GGAA | 2374 | 1.254 | AUUA | 5529 | 1.254 |
| 64 | GGCA | 2986 | 1.229 | AUAU | 3662 | 1.357 | AGGG | 2369 | 1.254 | GGAA | 5522 | 1.254 |
| 65 | UGUA | 2968 | 1.229 | GGAA | 3643 | 1.357 | UUGC | 2365 | 1.254 | AUAG | 5459 | 1.229 |
| 66 | GCUC | 2966 | 1.229 | GAGA | 3633 | 1.331 | GCAA | 2362 | 1.254 | AGGG | 5416 | 1.229 |
| 67 | CAAA | 2958 | 1.229 | CAUC | 3585 | 1.331 | GUUA | 2356 | 1.229 | AGGU | 5402 | 1.229 |

| | | | | | | | | | | | | |
|---|---|---|---|---|---|---|---|---|---|---|---|---|
| 68 | GACC | 2945 | 1.229 | GAUA | 3535 | 1.306 | GAUC | 2356 | 1.229 | AAUG | 5382 | 1.229 |
| 69 | GAUA | 2901 | 1.203 | CAAU | 3497 | 1.280 | GAGG | 2312 | 1.229 | GCUC | 5376 | 1.229 |
| 70 | GACA | 2896 | 1.203 | ACCA | 3480 | 1.280 | AUGA | 2309 | 1.229 | UACC | 5345 | 1.203 |
| 71 | CGGC | 2892 | 1.203 | CGAC | 3426 | 1.254 | UACA | 2308 | 1.229 | UUGC | 5344 | 1.203 |
| 72 | GAGG | 2887 | 1.203 | UUAA | 3416 | 1.254 | GUAU | 2305 | 1.203 | GUAU | 5341 | 1.203 |
| 73 | AUAU | 2879 | 1.203 | GCAG | 3393 | 1.254 | GAGA | 2296 | 1.203 | GAGA | 5327 | 1.203 |
| 74 | CACC | 2877 | 1.203 | CACC | 3389 | 1.254 | AAAU | 2279 | 1.203 | UAGU | 5316 | 1.203 |
| 75 | AUUA | 2858 | 1.178 | ACAU | 3368 | 1.254 | AGUG | 2240 | 1.178 | GCCA | 5289 | 1.203 |
| 76 | AGGG | 2849 | 1.178 | CUAC | 3366 | 1.254 | GCCA | 2235 | 1.178 | ACAA | 5266 | 1.203 |
| 77 | AUGG | 2839 | 1.178 | CACA | 3278 | 1.203 | GUUC | 2229 | 1.178 | CUAC | 5260 | 1.178 |
| 78 | UGGC | 2839 | 1.178 | CAGA | 3207 | 1.178 | CAUC | 2223 | 1.178 | AUGA | 5226 | 1.178 |
| 79 | CGAC | 2812 | 1.152 | GCCA | 3188 | 1.178 | AGGU | 2209 | 1.152 | GUUA | 5215 | 1.178 |
| 80 | GGAA | 2810 | 1.152 | GGUA | 3185 | 1.178 | GGGA | 2203 | 1.152 | UCAC | 5210 | 1.178 |
| 81 | UUAG | 2808 | 1.152 | ACUC | 3170 | 1.178 | GCUC | 2198 | 1.152 | GACA | 5170 | 1.178 |
| 82 | ACUC | 2790 | 1.152 | UGAA | 3169 | 1.178 | AUAU | 2191 | 1.152 | AGAG | 5151 | 1.178 |
| 83 | UAAU | 2787 | 1.152 | AUUA | 3119 | 1.152 | ACUC | 2171 | 1.152 | UGGA | 5131 | 1.152 |
| 84 | GAGA | 2772 | 1.152 | GGCC | 3075 | 1.126 | UAUG | 2167 | 1.152 | GAAG | 5121 | 1.152 |
| 85 | UAUG | 2768 | 1.152 | AUUC | 3064 | 1.126 | CGGC | 2163 | 1.152 | GACC | 5121 | 1.152 |
| 86 | UUAA | 2761 | 1.152 | AAUU | 3062 | 1.126 | UACC | 2144 | 1.126 | GGGA | 5113 | 1.152 |
| 87 | CACA | 2756 | 1.152 | AUGA | 3043 | 1.126 | UGUC | 2140 | 1.126 | GAGG | 5093 | 1.152 |
| 88 | GGUC | 2754 | 1.152 | GAGG | 3021 | 1.126 | CUAC | 2136 | 1.126 | ACUC | 5069 | 1.152 |
| 89 | GUAU | 2736 | 1.126 | ACCC | 3020 | 1.126 | ACAG | 2133 | 1.126 | CCAC | 5034 | 1.126 |
| 90 | AACG | 2731 | 1.126 | CAUA | 2996 | 1.101 | UAGU | 2117 | 1.126 | UAAU | 5031 | 1.126 |
| 91 | GUGC | 2728 | 1.126 | GGCA | 2970 | 1.101 | UUAG | 2110 | 1.101 | AUAU | 5017 | 1.126 |

| | | | | | | | | | | | | |
|---|---|---|---|---|---|---|---|---|---|---|---|---|
| 92 | UAGU | 2708 | 1.126 | GCGC | 2964 | 1.101 | UGGA | 2106 | 1.101 | GUUC | 5017 | 1.126 |
| 93 | UGAC | 2696 | 1.126 | AACG | 2951 | 1.101 | UAAU | 2092 | 1.101 | AUGG | 4995 | 1.126 |
| 94 | UACC | 2691 | 1.126 | UGGC | 2934 | 1.075 | GACA | 2090 | 1.101 | CAAA | 4983 | 1.126 |
| 95 | UCAC | 2674 | 1.101 | UAGG | 2932 | 1.075 | ACAA | 2077 | 1.101 | AUCA | 4815 | 1.101 |
| 96 | GCCC | 2654 | 1.101 | GGGC | 2926 | 1.075 | UUUA | 2072 | 1.101 | GCUA | 4783 | 1.075 |
| 97 | CAGG | 2650 | 1.101 | GUAU | 2900 | 1.075 | GGAG | 2058 | 1.075 | CACA | 4777 | 1.075 |
| 98 | ACCA | 2648 | 1.101 | CUAA | 2864 | 1.050 | GCUA | 2058 | 1.075 | ACCA | 4751 | 1.075 |
| 99 | CAUA | 2645 | 1.101 | AUCA | 2856 | 1.050 | GACC | 2031 | 1.075 | UUUA | 4728 | 1.075 |
| 100 | AGUG | 2644 | 1.101 | AUCC | 2839 | 1.050 | UGAA | 2025 | 1.075 | CAGA | 4718 | 1.075 |
| 101 | GGAG | 2639 | 1.101 | CCAA | 2820 | 1.050 | UCAC | 2024 | 1.075 | AGAU | 4717 | 1.075 |
| 102 | CAGA | 2635 | 1.101 | UAGU | 2815 | 1.050 | CAAG | 2017 | 1.075 | UAUG | 4683 | 1.050 |
| 103 | GAAU | 2629 | 1.101 | AGGG | 2760 | 1.024 | AUUG | 2009 | 1.050 | UGUC | 4679 | 1.050 |
| 104 | AAUU | 2614 | 1.075 | GCAU | 2759 | 1.024 | GCAG | 2003 | 1.050 | CAUA | 4653 | 1.050 |
| 105 | AGAU | 2613 | 1.075 | GGAG | 2749 | 1.024 | AGAU | 2000 | 1.050 | CGAC | 4650 | 1.050 |
| 106 | AUUC | 2585 | 1.075 | GCCC | 2712 | 0.998 | CAUA | 1998 | 1.050 | UGAA | 4650 | 1.050 |
| 107 | CAAU | 2579 | 1.075 | UUAG | 2638 | 0.973 | CAAA | 1976 | 1.050 | UGCA | 4644 | 1.050 |
| 108 | AGGU | 2537 | 1.050 | GGUC | 2631 | 0.973 | AUCA | 1966 | 1.024 | ACAG | 4546 | 1.024 |
| 109 | AUGA | 2530 | 1.050 | UCAA | 2619 | 0.973 | CCAC | 1962 | 1.024 | CACC | 4487 | 1.024 |
| 110 | GAUG | 2518 | 1.050 | UGUA | 2581 | 0.947 | ACCA | 1939 | 1.024 | GAAU | 4485 | 1.024 |
| 111 | ACCC | 2511 | 1.050 | ACUA | 2574 | 0.947 | AUGU | 1938 | 1.024 | UGCC | 4480 | 1.024 |
| 112 | AUCA | 2503 | 1.024 | UAUG | 2519 | 0.922 | GAUG | 1936 | 1.024 | AAUU | 4461 | 0.998 |
| 113 | AUUG | 2432 | 0.998 | CAGG | 2515 | 0.922 | UGCA | 1931 | 1.024 | GGAG | 4444 | 0.998 |
| 114 | GCUA | 2422 | 0.998 | AGGU | 2505 | 0.922 | GUGA | 1931 | 1.024 | UUAG | 4434 | 0.998 |
| 115 | ACAU | 2419 | 0.998 | GGAU | 2468 | 0.922 | UUUC | 1928 | 1.024 | GCAG | 4431 | 0.998 |

| | | | | | | | | | | | | |
|---|---|---|---|---|---|---|---|---|---|---|---|---|
| 116 | AGCG | 2401 | 0.998 | ACGA | 2454 | 0.896 | AAUU | 1924 | 1.024 | CAAG | 4392 | 0.998 |
| 117 | GUUA | 2371 | 0.973 | GCUC | 2428 | 0.896 | CAGA | 1916 | 0.998 | AGUG | 4389 | 0.998 |
| 118 | CGCC | 2367 | 0.973 | GAUG | 2426 | 0.896 | CAGG | 1899 | 0.998 | AUGU | 4380 | 0.998 |
| 119 | CUAG | 2365 | 0.973 | AUGG | 2405 | 0.896 | UGCC | 1892 | 0.998 | GUGA | 4369 | 0.998 |
| 120 | CAGU | 2359 | 0.973 | AACU | 2396 | 0.896 | CGUA | 1884 | 0.998 | UCGC | 4358 | 0.998 |
| 121 | GCAU | 2353 | 0.973 | CGAA | 2349 | 0.870 | CUGC | 1882 | 0.998 | GAGU | 4354 | 0.973 |
| 122 | GUUC | 2351 | 0.973 | AGUG | 2347 | 0.870 | GAAU | 1867 | 0.973 | GCCC | 4344 | 0.973 |
| 123 | CAUG | 2334 | 0.973 | GAGU | 2337 | 0.870 | AGUU | 1854 | 0.973 | GCGA | 4272 | 0.973 |
| 124 | UGAA | 2317 | 0.947 | CGGC | 2320 | 0.845 | UCGC | 1851 | 0.973 | CGUA | 4242 | 0.947 |
| 125 | CUAA | 2305 | 0.947 | GUGC | 2293 | 0.845 | AGCG | 1850 | 0.973 | GUCA | 4240 | 0.947 |
| 126 | UUGC | 2278 | 0.947 | UGGA | 2265 | 0.845 | CGAC | 1822 | 0.973 | AUCC | 4225 | 0.947 |
| 127 | GCGA | 2260 | 0.947 | GCUA | 2260 | 0.845 | GAGU | 1821 | 0.947 | AGUU | 4212 | 0.947 |
| 128 | UGGA | 2256 | 0.922 | GCGA | 2247 | 0.819 | UUGA | 1804 | 0.947 | GGAU | 4193 | 0.947 |
| 129 | GGGA | 2246 | 0.922 | AGCG | 2226 | 0.819 | GGUG | 1802 | 0.947 | UUUC | 4168 | 0.947 |
| 130 | AUGU | 2217 | 0.922 | CAGU | 2220 | 0.819 | UUAU | 1776 | 0.947 | CAGG | 4164 | 0.947 |
| 131 | UUUA | 2209 | 0.922 | UGCC | 2205 | 0.819 | GCCC | 1767 | 0.922 | ACCC | 4160 | 0.947 |
| 132 | UGCA | 2208 | 0.922 | GGGA | 2200 | 0.819 | GUCA | 1767 | 0.922 | GAUG | 4138 | 0.947 |
| 133 | GAGU | 2198 | 0.922 | UUAU | 2182 | 0.794 | GCGA | 1757 | 0.922 | CAAU | 4126 | 0.922 |
| 134 | ACUA | 2194 | 0.896 | CCAG | 2147 | 0.794 | CACA | 1753 | 0.922 | UAUU | 4119 | 0.922 |
| 135 | AUCC | 2190 | 0.896 | UUGC | 2137 | 0.794 | GUGG | 1742 | 0.922 | ACUA | 4106 | 0.922 |
| 136 | CGUA | 2169 | 0.896 | GUUA | 2117 | 0.794 | AUCC | 1735 | 0.922 | UUGA | 4102 | 0.922 |
| 137 | GUCA | 2162 | 0.896 | UCAG | 2102 | 0.768 | ACUA | 1712 | 0.896 | ACAU | 4095 | 0.922 |
| 138 | UACG | 2159 | 0.896 | UGAG | 2084 | 0.768 | ACCC | 1701 | 0.896 | CAGU | 4072 | 0.922 |
| 139 | CUGC | 2155 | 0.896 | CGCC | 2084 | 0.768 | UGAG | 1700 | 0.896 | UUAU | 4066 | 0.922 |

| | | | | | | | | | | | | |
|---|---|---|---|---|---|---|---|---|---|---|---|---|
| 140 | CCGC | 2152 | 0.896 | UGCA | 2079 | 0.768 | CACC | 1699 | 0.896 | CUGC | 4031 | 0.922 |
| 141 | CGCA | 2141 | 0.896 | AGCU | 2069 | 0.768 | CUAA | 1687 | 0.896 | UCAA | 3997 | 0.896 |
| 142 | UGAG | 2132 | 0.870 | UAUU | 2059 | 0.768 | UAUU | 1682 | 0.896 | AGCG | 3993 | 0.896 |
| 143 | UCAG | 2123 | 0.870 | CUAG | 2051 | 0.768 | CAGU | 1676 | 0.896 | CUAA | 3974 | 0.896 |
| 144 | UAUU | 2119 | 0.870 | AUUG | 2016 | 0.742 | GGAU | 1668 | 0.870 | CGCC | 3929 | 0.896 |
| 145 | CCAG | 2116 | 0.870 | GUUC | 2012 | 0.742 | CAAU | 1667 | 0.870 | GGGU | 3876 | 0.870 |
| 146 | UUAU | 2083 | 0.870 | ACGG | 2008 | 0.742 | CAUG | 1660 | 0.870 | AUUG | 3859 | 0.870 |
| 147 | CCAA | 2080 | 0.870 | CAUG | 2005 | 0.742 | AACG | 1657 | 0.870 | UGGU | 3859 | 0.870 |
| 148 | UCAA | 2055 | 0.845 | AUGU | 2003 | 0.742 | GUUG | 1652 | 0.870 | CGUC | 3829 | 0.870 |
| 149 | ACGG | 2052 | 0.845 | GUCA | 1998 | 0.742 | ACAU | 1646 | 0.870 | GCAU | 3801 | 0.870 |
| 150 | UGCC | 2037 | 0.845 | UCGC | 1972 | 0.717 | CCGC | 1618 | 0.845 | GUCC | 3796 | 0.870 |
| 151 | GGAU | 2035 | 0.845 | CGCA | 1971 | 0.717 | ACGG | 1616 | 0.845 | GUGG | 3790 | 0.845 |
| 152 | GUGG | 2035 | 0.845 | AGUU | 1964 | 0.717 | CGCC | 1615 | 0.845 | AACG | 3778 | 0.845 |
| 153 | GCGG | 2030 | 0.845 | CCAU | 1952 | 0.717 | GGGU | 1615 | 0.845 | CCGC | 3755 | 0.845 |
| 154 | CGUC | 2021 | 0.845 | UGUC | 1948 | 0.717 | UUGG | 1610 | 0.845 | GGUU | 3723 | 0.845 |
| 155 | AGUU | 2021 | 0.845 | GAUU | 1941 | 0.717 | CUAG | 1610 | 0.845 | AGCU | 3679 | 0.845 |
| 156 | CACG | 2012 | 0.845 | UGAU | 1919 | 0.717 | AUUU | 1601 | 0.845 | GGUG | 3651 | 0.819 |
| 157 | CGAA | 2005 | 0.819 | GACG | 1916 | 0.717 | UCAA | 1598 | 0.845 | GGGG | 3649 | 0.819 |
| 158 | GAUU | 1984 | 0.819 | CGUA | 1915 | 0.717 | GGUU | 1594 | 0.845 | ACGA | 3630 | 0.819 |
| 159 | GGUG | 1974 | 0.819 | UACG | 1901 | 0.691 | GCGG | 1578 | 0.819 | CGCA | 3616 | 0.819 |
| 160 | AUUU | 1969 | 0.819 | GUGA | 1891 | 0.691 | UGGU | 1572 | 0.819 | GAUU | 3601 | 0.819 |
| 161 | UCGC | 1964 | 0.819 | UUUA | 1833 | 0.666 | ACGA | 1571 | 0.819 | UGAG | 3585 | 0.819 |
| 162 | GACG | 1959 | 0.819 | CGAG | 1822 | 0.666 | CGUC | 1558 | 0.819 | UUCA | 3583 | 0.819 |
| 163 | GUGA | 1948 | 0.819 | CUAU | 1817 | 0.666 | GCAU | 1555 | 0.819 | GGCG | 3515 | 0.794 |

| | | | | | | | | | | | | |
|---|---|---|---|---|---|---|---|---|---|---|---|---|
| 164 | UUGA | 1932 | 0.794 | GUCC | 1816 | 0.666 | GGGG | 1555 | 0.819 | AUUU | 3513 | 0.794 |
| 165 | GGGG | 1925 | 0.794 | CCCA | 1813 | 0.666 | GUCC | 1546 | 0.819 | CGGA | 3463 | 0.794 |
| 166 | CAUU | 1921 | 0.794 | CCGC | 1761 | 0.640 | GGCG | 1540 | 0.819 | UGAU | 3446 | 0.768 |
| 167 | AACU | 1907 | 0.794 | CAUU | 1759 | 0.640 | UGGG | 1538 | 0.819 | UGGG | 3437 | 0.768 |
| 168 | AGCU | 1899 | 0.794 | CGAU | 1735 | 0.640 | AGCU | 1526 | 0.794 | CAUG | 3417 | 0.768 |
| 169 | GGCG | 1895 | 0.794 | UCAU | 1733 | 0.640 | UGUG | 1516 | 0.794 | UCUC | 3405 | 0.768 |
| 170 | CUAU | 1885 | 0.768 | UCCA | 1716 | 0.640 | GAUU | 1504 | 0.794 | GUUG | 3375 | 0.768 |
| 171 | ACGA | 1876 | 0.768 | UUGA | 1716 | 0.640 | CGCA | 1474 | 0.768 | ACGG | 3373 | 0.768 |
| 172 | UGUC | 1868 | 0.768 | UACU | 1694 | 0.614 | UUCA | 1457 | 0.768 | GCGG | 3337 | 0.742 |
| 173 | CGAG | 1860 | 0.768 | CGUC | 1690 | 0.614 | GUGU | 1423 | 0.742 | UUGU | 3307 | 0.742 |
| 174 | GUCC | 1836 | 0.768 | CCCC | 1678 | 0.614 | GUUU | 1422 | 0.742 | UUGG | 3306 | 0.742 |
| 175 | UUGG | 1818 | 0.742 | AUUU | 1667 | 0.614 | UCAG | 1412 | 0.742 | GUGU | 3305 | 0.742 |
| 176 | GUUG | 1800 | 0.742 | GACU | 1665 | 0.614 | UACG | 1411 | 0.742 | CGAA | 3299 | 0.742 |
| 177 | CCUC | 1796 | 0.742 | CACG | 1654 | 0.614 | UCUC | 1411 | 0.742 | GGCU | 3287 | 0.742 |
| 178 | UUUC | 1793 | 0.742 | CUGC | 1634 | 0.614 | UGAU | 1403 | 0.742 | AACU | 3250 | 0.742 |
| 179 | CCCA | 1771 | 0.742 | UUUC | 1597 | 0.589 | CGAA | 1403 | 0.742 | CCAA | 3247 | 0.742 |
| 180 | CCCC | 1759 | 0.717 | ACUG | 1583 | 0.589 | GACG | 1396 | 0.742 | CAUU | 3232 | 0.742 |
| 181 | UGAU | 1756 | 0.717 | UUCA | 1580 | 0.589 | UCUA | 1383 | 0.717 | UACG | 3205 | 0.717 |
| 182 | AUCG | 1723 | 0.717 | CGGA | 1557 | 0.563 | UUUG | 1379 | 0.717 | CUAG | 3190 | 0.717 |
| 183 | UUCA | 1719 | 0.717 | ACGU | 1554 | 0.563 | GGCU | 1377 | 0.717 | UCCA | 3186 | 0.717 |
| 184 | GCUG | 1703 | 0.717 | UCUC | 1547 | 0.563 | CUUA | 1374 | 0.717 | UCAG | 3152 | 0.717 |
| 185 | CUUC | 1697 | 0.691 | GCGG | 1543 | 0.563 | CUUC | 1367 | 0.717 | ACGU | 3149 | 0.717 |
| 186 | UGGU | 1693 | 0.691 | AUCG | 1533 | 0.563 | CUAU | 1364 | 0.717 | GUUU | 3148 | 0.717 |
| 187 | CCAU | 1688 | 0.691 | CCUC | 1477 | 0.538 | CCAA | 1349 | 0.717 | CUUA | 3141 | 0.717 |

| | | | | | | | | | | | | |
|---|---|---|---|---|---|---|---|---|---|---|---|---|
| 188 | UGGG | 1685 | 0.691 | CUCA | 1463 | 0.538 | UUCC | 1345 | 0.717 | CCUC | 3092 | 0.691 |
| 189 | CUCA | 1663 | 0.691 | UCCC | 1452 | 0.538 | CCAG | 1343 | 0.717 | GCGU | 2986 | 0.666 |
| 190 | UCCA | 1662 | 0.691 | CACU | 1448 | 0.538 | CGGA | 1340 | 0.717 | UGUG | 2981 | 0.666 |
| 191 | CUUA | 1654 | 0.691 | GGCG | 1412 | 0.512 | CCUC | 1339 | 0.717 | CUUC | 2963 | 0.666 |
| 192 | GGGU | 1654 | 0.691 | UUCC | 1345 | 0.486 | ACGU | 1336 | 0.717 | GACG | 2955 | 0.666 |
| 193 | CGGA | 1623 | 0.666 | ACCG | 1340 | 0.486 | UUGU | 1327 | 0.691 | UGUU | 2952 | 0.666 |
| 194 | UACU | 1622 | 0.666 | GGUG | 1319 | 0.486 | ACUG | 1318 | 0.691 | UCUA | 2940 | 0.666 |
| 195 | ACUG | 1620 | 0.666 | CUUA | 1318 | 0.486 | GCUG | 1307 | 0.691 | UACU | 2932 | 0.666 |
| 196 | GGUU | 1610 | 0.666 | UCGA | 1306 | 0.486 | UCCA | 1302 | 0.691 | CUCA | 2930 | 0.666 |
| 197 | GGCU | 1584 | 0.666 | UCUA | 1292 | 0.486 | CAUU | 1298 | 0.691 | UUCC | 2917 | 0.666 |
| 198 | GACU | 1583 | 0.666 | GGGU | 1290 | 0.486 | AACU | 1298 | 0.691 | UCGA | 2901 | 0.666 |
| 199 | GCGU | 1562 | 0.640 | GUGG | 1284 | 0.486 | AUCG | 1271 | 0.666 | CCAG | 2896 | 0.666 |
| 200 | UCAU | 1558 | 0.640 | ACUU | 1274 | 0.461 | UGUU | 1250 | 0.666 | UCAU | 2881 | 0.640 |
| 201 | GUGU | 1556 | 0.640 | UGGU | 1269 | 0.461 | CUGG | 1247 | 0.666 | CUAU | 2869 | 0.640 |
| 202 | UCUC | 1554 | 0.640 | GGCU | 1263 | 0.461 | CCCA | 1247 | 0.666 | UUUG | 2849 | 0.640 |
| 203 | CGAU | 1549 | 0.640 | CUCC | 1260 | 0.461 | CUCA | 1242 | 0.666 | AUCG | 2822 | 0.640 |
| 204 | CUGG | 1540 | 0.640 | GGGG | 1236 | 0.461 | UUUU | 1239 | 0.666 | UCCC | 2799 | 0.640 |
| 205 | ACGU | 1529 | 0.640 | AUCU | 1226 | 0.461 | GCGU | 1237 | 0.640 | CCCA | 2791 | 0.640 |
| 206 | CUCC | 1524 | 0.640 | GCGU | 1223 | 0.461 | CGAG | 1236 | 0.640 | CGGU | 2715 | 0.614 |
| 207 | UCUA | 1513 | 0.614 | CUGA | 1214 | 0.461 | CGGG | 1207 | 0.640 | GACU | 2714 | 0.614 |
| 208 | CGCG | 1510 | 0.614 | GGUU | 1214 | 0.461 | CACG | 1203 | 0.640 | CUGA | 2694 | 0.614 |
| 209 | UUUG | 1503 | 0.614 | CCUA | 1211 | 0.435 | CUGA | 1178 | 0.614 | ACUG | 2694 | 0.614 |
| 210 | CGGG | 1499 | 0.614 | CUUC | 1196 | 0.435 | UGCG | 1169 | 0.614 | UUUU | 2693 | 0.614 |
| 211 | UGUG | 1497 | 0.614 | ACCU | 1185 | 0.435 | UACU | 1163 | 0.614 | CGAG | 2633 | 0.589 |

| | | | | | | | | | | | | |
|---|---|---|---|---|---|---|---|---|---|---|---|---|
| 212 | GCCG | 1495 | 0.614 | GCUG | 1180 | 0.435 | UCAU | 1152 | 0.614 | GCUG | 2623 | 0.589 |
| 213 | UGCG | 1477 | 0.614 | UUGG | 1169 | 0.435 | GUCG | 1129 | 0.589 | GCUU | 2619 | 0.589 |
| 214 | ACCG | 1474 | 0.614 | GUUG | 1159 | 0.435 | ACUU | 1125 | 0.589 | CGAU | 2604 | 0.589 |
| 215 | UUGU | 1461 | 0.614 | UGGG | 1158 | 0.435 | GACU | 1117 | 0.589 | CACG | 2590 | 0.589 |
| 216 | CACU | 1457 | 0.614 | GUUU | 1128 | 0.410 | CCAU | 1111 | 0.589 | UGCG | 2582 | 0.589 |
| 217 | GUUU | 1421 | 0.589 | GUGU | 1090 | 0.410 | UCGA | 1109 | 0.589 | CCAU | 2561 | 0.589 |
| 218 | GUCG | 1408 | 0.589 | UUGU | 1049 | 0.384 | CGGU | 1099 | 0.589 | CGGG | 2551 | 0.589 |
| 219 | CGUG | 1405 | 0.589 | GCUU | 1048 | 0.384 | UCCC | 1093 | 0.563 | CUCC | 2550 | 0.589 |
| 220 | UCCC | 1401 | 0.589 | CCGA | 1047 | 0.384 | UGCU | 1088 | 0.563 | CCCC | 2518 | 0.563 |
| 221 | UUCC | 1395 | 0.589 | GCCG | 1043 | 0.384 | CGUG | 1081 | 0.563 | ACUU | 2469 | 0.563 |
| 222 | UCGA | 1392 | 0.589 | UGUG | 1029 | 0.384 | ACCG | 1069 | 0.563 | CUGG | 2435 | 0.563 |
| 223 | ACUU | 1392 | 0.589 | GUCG | 1001 | 0.358 | CGAU | 1067 | 0.563 | UGCU | 2397 | 0.538 |
| 224 | CGGU | 1372 | 0.563 | UGCG | 993 | 0.358 | CUUG | 1058 | 0.563 | CACU | 2366 | 0.538 |
| 225 | CUGA | 1350 | 0.563 | GCCU | 992 | 0.358 | CCCC | 1049 | 0.563 | AUCU | 2323 | 0.538 |
| 226 | GCUU | 1349 | 0.563 | CGGG | 925 | 0.333 | CUCC | 1045 | 0.563 | CCUA | 2282 | 0.512 |
| 227 | CUUG | 1321 | 0.538 | UUUG | 925 | 0.333 | CCUA | 1032 | 0.538 | GUCG | 2255 | 0.512 |
| 228 | UCGG | 1302 | 0.538 | UGUU | 923 | 0.333 | GCUU | 1031 | 0.538 | CGUG | 2248 | 0.512 |
| 229 | AUCU | 1294 | 0.538 | CGCG | 918 | 0.333 | GCCG | 1025 | 0.538 | UCGG | 2203 | 0.486 |
| 230 | CCUA | 1285 | 0.538 | CGCU | 910 | 0.333 | UCGG | 1019 | 0.538 | UCGU | 2146 | 0.486 |
| 231 | UGUU | 1272 | 0.538 | CGGU | 900 | 0.333 | AUCU | 1015 | 0.538 | GCCG | 2145 | 0.486 |
| 232 | UUUU | 1232 | 0.512 | UGCU | 895 | 0.333 | CGCG | 978 | 0.512 | CGCU | 2131 | 0.486 |
| 233 | CCGA | 1208 | 0.512 | CUGG | 894 | 0.333 | CACU | 974 | 0.512 | GUCU | 2126 | 0.486 |
| 234 | UGCU | 1186 | 0.486 | UCGG | 879 | 0.333 | CUGU | 958 | 0.512 | CGCG | 2110 | 0.486 |
| 235 | CGCU | 1180 | 0.486 | UUUU | 869 | 0.333 | CUUU | 942 | 0.486 | GCCU | 2108 | 0.486 |

| | | | | | | | | | | | | |
|---|---|---|---|---|---|---|---|---|---|---|---|---|
| 236 | CCGG | 1163 | 0.486 | CGUG | 846 | 0.307 | UCGU | 906 | 0.486 | CGUU | 2088 | 0.461 |
| 237 | CUUU | 1129 | 0.461 | GUCU | 840 | 0.307 | CGUU | 899 | 0.486 | CUGU | 2085 | 0.461 |
| 238 | CGUU | 1128 | 0.461 | CGUU | 785 | 0.282 | UUCG | 894 | 0.461 | ACCU | 2079 | 0.461 |
| 239 | UUCG | 1125 | 0.461 | CUGU | 752 | 0.282 | GUCU | 889 | 0.461 | ACCG | 2036 | 0.461 |
| 240 | CUGU | 1105 | 0.461 | UCGU | 738 | 0.282 | CCGA | 888 | 0.461 | CCGA | 1978 | 0.435 |
| 241 | ACCU | 1097 | 0.461 | CUUG | 737 | 0.282 | CGCU | 878 | 0.461 | CUUG | 1942 | 0.435 |
| 242 | CCUG | 1071 | 0.435 | CUUU | 701 | 0.256 | ACCU | 864 | 0.461 | CUUU | 1907 | 0.435 |
| 243 | GCCU | 1069 | 0.435 | CCGU | 694 | 0.256 | UCUG | 864 | 0.461 | UUCG | 1860 | 0.410 |
| 244 | UCGU | 1055 | 0.435 | CCGG | 684 | 0.256 | CCGG | 853 | 0.461 | UCUG | 1764 | 0.410 |
| 245 | CUCG | 1045 | 0.435 | UUCG | 684 | 0.256 | GCCU | 838 | 0.435 | CCGU | 1674 | 0.384 |
| 246 | GUCU | 1028 | 0.435 | CUCG | 662 | 0.256 | CUCG | 776 | 0.410 | UCUU | 1651 | 0.384 |
| 247 | UCUG | 1026 | 0.435 | UCUG | 647 | 0.230 | CCGU | 774 | 0.410 | CCGG | 1650 | 0.384 |
| 248 | CCCG | 1003 | 0.410 | CCCU | 620 | 0.230 | UUCU | 771 | 0.410 | UUCU | 1639 | 0.358 |
| 249 | CCGU | 969 | 0.410 | UCUU | 618 | 0.230 | UCUU | 765 | 0.410 | CUCG | 1521 | 0.333 |
| 250 | CCUU | 878 | 0.358 | UUCU | 615 | 0.230 | CCUG | 760 | 0.410 | CCUU | 1434 | 0.333 |
| 251 | UCCG | 873 | 0.358 | CCUG | 602 | 0.230 | CCUU | 672 | 0.358 | CUCU | 1433 | 0.333 |
| 252 | UCUU | 816 | 0.333 | UCCG | 580 | 0.205 | CUCU | 614 | 0.333 | CCUG | 1370 | 0.307 |
| 253 | CUCU | 812 | 0.333 | CCUU | 576 | 0.205 | UCCG | 598 | 0.307 | UCCG | 1272 | 0.282 |
| 254 | UUCU | 774 | 0.333 | CCCG | 573 | 0.205 | CCCG | 583 | 0.307 | CCCU | 1260 | 0.282 |
| 255 | CCCU | 732 | 0.307 | CUCU | 554 | 0.205 | CCCU | 535 | 0.282 | UCCU | 1236 | 0.282 |
| 256 | UCCU | 712 | 0.307 | UCCU | 550 | 0.205 | UCCU | 521 | 0.282 | CCCG | 1228 | 0.282 |
| Frequency range | 9 | | | 20 | | | 8 | | | 9 | | |

**Table S3. Global overview of sequencing outcome from four strands experiments.**

| Reactions with 250 nM each A:a and B:b duplex | | | | | | | | | | |
|---|---|---|---|---|---|---|---|---|---|---|
| X:Y | Seq_reads total | Seq_reads after filter1 | Seq_reads after filter2 | Final Seq_reads after filter3 | Final seq_reads remained (P1+P2+P3+P4) | P1 | P2 | P3 | P4 | (P2+P3) / (P1+P2+P3+P4) |
| C:G | 986419 | 742264 | 494097 | 494043 | 50 % | 102665 | 147191 | 62553 | 181634 | 42 % |
| G:C | 830715 | 619338 | 424258 | 424220 | 51 % | 106620 | 119612 | 86252 | 111736 | 49 % |
| A:U | 1117599 | 825415 | 593867 | 593808 | 53 % | 258097 | 142977 | 91043 | 101691 | 39 % |
| U:A | 1164835 | 883594 | 589907 | 589844 | 51 % | 200243 | 180783 | 77364 | 131454 | 44 % |
| G:U | 1112853 | 846177 | 575240 | 575178 | 52 % | 162424 | 129437 | 132519 | 150798 | 46 % |
| Average | | | | | 51 % | | | | | 44 % |
| Reactions with 5 nM each A:a and B:b duplex | | | | | | | | | | |
| X:Y | Seq_reads total | Seq_reads after filter1 | Seq_reads after filter2 | Final Seq_reads after filter3 | Final seq_reads remained (P1+P2+P3+P4) | P1 | P2 | P3 | P4 | (P2+P3) / (P1+P2+P3+P4) |
| C:G | 626598 | 459541 | 322580 | 322545 | 50 % | 82803 | 5242 | 1614 | 284138 | 2 % |
| G:C | 812403 | 605961 | 444034 | 443997 | 51 % | 122927 | 8005 | 2225 | 189388 | 3 % |
| A:U | 925752 | 705117 | 492870 | 492821 | 55 % | 220504 | 7200 | 2401 | 213892 | 2 % |
| U:A | 797618 | 598905 | 427343 | 427299 | 53 % | 256511 | 9914 | 3641 | 222755 | 3 % |
| G:U | 721144 | 531213 | 370581 | 370556 | 54 % | 190328 | 12147 | 12158 | 212666 | 6 % |
| U:G | 626598 | 459541 | 322580 | 322545 | 51 % | 95968 | 7713 | 1491 | 265384 | 2 % |
| Average | | | | | 52 % | | | | | 3 % |

**Table S4. Global overview of sequencing outcome from two-strands reactions.**

| Reactions with 5 nM A:a duplex concentration (replicate 1) | | | | | |
|---|---|---|---|---|---|
| X:Y | Seq_reads total | Seq_reads after filter1 | Seq_reads after filter2 | Final Seq_reads after filter3 | Final seq_reads remained, P1 |
| C:G | 1557292 | 1047742 | 665331 | 665093 | 43 % |
| G:C | 1320317 | 873066 | 506164 | 505983 | 38 % |
| A:U | 1905215 | 1258230 | 923163 | 922777 | 48 % |
| U:A | 1817511 | 1252735 | 811183 | 810877 | 45 % |
| G:U | 1722321 | 1177677 | 765060 | 764788 | 44 % |
| Average | | | | | 44 % |
| Reactions with 5 nM A:a duplex concentration (replicate 2) | | | | | |
| X:Y | Seq_reads total | Seq_reads after filter1 | Seq_reads after filter2 | Final Seq_reads after filter3 | Final seq_reads remained, P1 |
| C:G | 474423 | 348181 | 217834 | 217818 | 46 % |
| G:C | 1136061 | 793258 | 502059 | 501912 | 44 % |
| A:U | 1312952 | 956260 | 695205 | 695008 | 53 % |
| U:A | 1225927 | 929016 | 586413 | 586279 | 48 % |
| G:U | 1023161 | 758386 | 507323 | 507238 | 50 % |
| U:G | 142720 | 104000 | 53839 | 53836 | 38 % |
| Average | | | | | 47 % |

**Table S5. Representative sequencing results of loop-closing product P1.** Reads are normalized by $\alpha_I$ (Table S2), direct readout is available in Supplementary excel file. Sequence patterns are colour-coded for the top 40 and bottom 40 sequences in each column.

| Rank order | C:G closing | Reads | G:C closing | Reads | A:U closing | Reads | U:A closing | Reads | G:U closing | Reads | U:G closing | Reads |
|---|---|---|---|---|---|---|---|---|---|---|---|---|
| 1 | UGCG | 5866 | UCCG | 10167 | GAGA | 16747 | GGGA | 13314 | CUUG | 16966 | UUCG | 1946 |
| 2 | UGUG | 4779 | UACG | 10057 | GGGA | 13633 | GAGA | 12983 | CUCG | 16167 | UGCG | 1599 |
| 3 | UUCG | 4683 | UCUG | 10006 | GUGA | 11903 | UCCG | 12188 | CUAG | 15888 | UUUG | 1561 |
| 4 | UUUG | 4516 | UCAG | 9589 | GCGA | 11857 | UUCG | 12147 | CGCG | 15235 | UUAG | 1451 |
| 5 | UACG | 4323 | UGAG | 9375 | CUCG | 10271 | GUGA | 11938 | CUGG | 15071 | UCCG | 1373 |
| 6 | UCCG | 4261 | UGCG | 9288 | UGCG | 9892 | UGCG | 11661 | UUCG | 13896 | CUGG | 1207 |
| 7 | UCAG | 4246 | GUGA | 9145 | UUAG | 9627 | GCGA | 11156 | CGAG | 11602 | CCCG | 1141 |
| 8 | UUAG | 4206 | GAGA | 8940 | CCCG | 9410 | UUUG | 10411 | GUGA | 11580 | GAGA | 1117 |
| 9 | UGAG | 4027 | UUUG | 8495 | CGCG | 9368 | UACG | 9295 | CACG | 11097 | CGUG | 1103 |
| 10 | UAUG | 3594 | UUCG | 8376 | CGAG | 9158 | UCUG | 9099 | CGUG | 10659 | UGUG | 1094 |
| 11 | UUGG | 3541 | GGGA | 8365 | CUGG | 9143 | UUAG | 8994 | UGCG | 10475 | UUGG | 1076 |
| 12 | UCGC | 3516 | UUGG | 8189 | UACG | 9016 | CCUG | 8670 | GCGA | 10330 | CCUG | 1068 |
| 13 | UAAG | 3442 | UAUG | 8051 | UGAG | 8950 | CACG | 8131 | CGGG | 10288 | CGAG | 1038 |
| 14 | UUGC | 3405 | GCGA | 8036 | UUUG | 8916 | UCAG | 8082 | CCCG | 10069 | GGUC | 1035 |
| 15 | UCGG | 3282 | UAAG | 7778 | CACG | 8810 | CCCG | 7865 | GAGA | 9934 | GUGA | 1014 |
| 16 | CCUG | 3187 | UGUG | 7595 | CAAG | 8778 | UUGG | 7730 | CCUG | 9895 | GGGA | 1003 |
| 17 | CAGG | 3067 | UUAG | 7588 | CUAG | 8715 | CGCG | 7710 | GGGA | 9824 | GCGA | 976 |
| 18 | UCUG | 3065 | CCGG | 7532 | CUUG | 8604 | UGUG | 7677 | CAUG | 9749 | CCAG | 971 |
| 19 | CGUG | 2899 | CUCG | 7455 | UCAG | 8567 | UGAG | 7617 | CCGG | 9409 | CUAG | 960 |

| | | | | | | | | | | | | |
|---|---|---|---|---|---|---|---|---|---|---|---|---|
| 20 | UAGC | 2888 | CCUG | 7375 | CAGG | 8468 | CAUG | 7546 | UACG | 9226 | CACG | 937 |
| 21 | CUCG | 2862 | UCGG | 7325 | UUGG | 8416 | UUUA | 7468 | CCAG | 9219 | CUCG | 907 |
| 22 | GGGA | 2839 | CUUG | 6983 | UUCG | 8346 | CUGG | 7396 | UCCG | 8659 | CGCG | 888 |
| 23 | CUGG | 2801 | CCCG | 6907 | CGGG | 8167 | CAGG | 7389 | GUCA | 8441 | CUUG | 876 |
| 24 | CGGG | 2751 | CGGG | 6885 | UUUA | 8088 | CGUG | 7197 | CAGG | 8343 | CCGG | 809 |
| 25 | CAAG | 2730 | CGCG | 6852 | UAAG | 8047 | CCGG | 7180 | CAAG | 8003 | UACG | 808 |
| 26 | CGCG | 2727 | UAGG | 6802 | UUGA | 7965 | CUCG | 7065 | UUAG | 7935 | CAUG | 805 |
| 27 | UGGG | 2721 | CACG | 6466 | CGUG | 7892 | UCGG | 6818 | UUCA | 7701 | UGAG | 803 |
| 28 | CGAG | 2709 | CUGG | 6457 | UGUG | 7846 | CUUG | 6779 | GUUA | 7586 | CGGG | 802 |
| 29 | CACG | 2556 | CGUG | 6378 | CCAG | 7747 | CGGG | 6777 | UGCA | 7554 | UCUG | 787 |
| 30 | UCAC | 2544 | UCUA | 6296 | CCUG | 7738 | UAUG | 6723 | GGUA | 7525 | CAAG | 785 |
| 31 | CAUG | 2536 | UGGG | 6229 | GAAA | 7514 | GAAA | 6717 | GUUC | 7254 | UGGG | 665 |
| 32 | GAGA | 2502 | CUAG | 6100 | UCCG | 7416 | CGAG | 6469 | UUUG | 6929 | GAAA | 605 |
| 33 | UAGG | 2489 | CAGG | 6019 | UCUG | 7327 | CAAG | 6298 | GGCA | 6796 | UAUG | 599 |
| 34 | GUGA | 2485 | CCAG | 5970 | CAUG | 7186 | CCAG | 6132 | UUGA | 6796 | UCAG | 579 |
| 35 | CCAG | 2434 | GAAA | 5934 | UUCA | 7150 | UAAG | 5775 | UCUA | 6738 | CAGG | 576 |
| 36 | CCCG | 2430 | UUCU | 5911 | CCGG | 7126 | CUAG | 5655 | GAAA | 6584 | UCGG | 572 |
| 37 | CCGG | 2426 | CGAG | 5820 | UAGG | 6953 | GUAG | 5482 | UUUA | 6527 | GGAA | 552 |
| 38 | UAAC | 2402 | UUUU | 5754 | UCGG | 6887 | UUGC | 5464 | UGGA | 6515 | UAGC | 514 |
| 39 | UGGC | 2348 | CAUG | 5742 | UGGG | 6780 | UGGG | 5323 | GUGG | 6453 | UAGG | 500 |
| 40 | UUAC | 2326 | GCUA | 5637 | UAUG | 6500 | UUUU | 5287 | GCUA | 6243 | UUGC | 466 |
| 41 | CUUG | 2300 | UUGA | 5590 | GUCA | 6060 | UAGG | 5217 | UCAG | 6231 | GGUA | 457 |
| 42 | CUAG | 2299 | GGAA | 5438 | UUUU | 5959 | UCGC | 5215 | CUUC | 6174 | GUAG | 448 |
| 43 | UGAC | 2211 | GCAA | 5257 | UCGA | 5623 | GCAG | 5176 | UGUA | 6083 | UAAG | 443 |

| | | | | | | | | | | | | |
|---|---|---|---|---|---|---|---|---|---|---|---|---|
| 44 | GCGA | 1952 | CAAG | 5018 | UGGA | 5454 | GAAG | 5037 | UCCA | 6038 | GUCA | 399 |
| 45 | UGCA | 1874 | UCUU | 4883 | UCCU | 5361 | GUCA | 4916 | GCAA | 5848 | UUUA | 391 |
| 46 | GUAG | 1866 | GAUA | 4605 | UAUA | 5355 | UAGC | 4862 | UGAG | 5785 | UGGC | 387 |
| 47 | UGUA | 1765 | GGUA | 4310 | UUCU | 5257 | UUGA | 4759 | UCGA | 5778 | GUAA | 365 |
| 48 | UCGA | 1577 | UCGC | 4249 | UUAA | 5133 | UAAC | 4667 | UUGG | 5777 | GCAA | 364 |
| 49 | UACA | 1458 | UUGC | 4234 | UUGC | 4941 | UUCU | 4485 | CUAC | 5759 | UUUU | 356 |
| 50 | UUGA | 1373 | UUAU | 4197 | UAAC | 4940 | UUCA | 4346 | UGUG | 5678 | UAAC | 347 |
| 51 | CUGA | 1329 | UGUU | 4179 | GUUA | 4817 | UCUA | 4269 | UCUG | 5514 | UCGC | 342 |
| 52 | UGUC | 1327 | UUGU | 4137 | UCGC | 4776 | GGUA | 4234 | CGGA | 5492 | GGAG | 332 |
| 53 | UUCU | 1326 | GUUA | 4105 | GGCA | 4693 | UGAC | 4202 | UAGG | 5489 | GCAG | 324 |
| 54 | GGUA | 1293 | UAUA | 4052 | UGAC | 4652 | GUUA | 4181 | GGAA | 5424 | UGUA | 321 |
| 55 | UCUA | 1292 | UUCA | 4019 | UUAC | 4651 | GGCA | 4016 | GUAC | 5289 | GAAG | 317 |
| 56 | UUUU | 1292 | UGUA | 3971 | UGCA | 4558 | GGAG | 3960 | CGCA | 5229 | GUUA | 309 |
| 57 | CGGA | 1244 | UGCU | 3941 | UGUA | 4549 | UUAC | 3819 | GUCC | 5222 | UUAC | 304 |
| 58 | UAUA | 1238 | UCAC | 3914 | UAGA | 4543 | UGUA | 3765 | UACA | 5116 | UUCA | 301 |
| 59 | CAGA | 1203 | UAGC | 3867 | GGAA | 4536 | UGGC | 3705 | CUCA | 5012 | UGAC | 294 |
| 60 | UUUC | 1187 | UAUU | 3861 | UUAU | 4429 | UGGA | 3548 | GUAA | 4946 | GGCA | 291 |
| 61 | UGCU | 1179 | UGAU | 3841 | UCUA | 4425 | UGCA | 3443 | UCGG | 4931 | UUAU | 278 |
| 62 | UGCC | 1175 | UAAC | 3823 | UCAC | 4327 | UCCA | 3428 | UUAC | 4868 | GUUC | 277 |
| 63 | UUCC | 1157 | GUCA | 3784 | UAGC | 4312 | UCGA | 3413 | CUGC | 4862 | GGUG | 268 |
| 64 | GGAA | 1154 | UCGA | 3756 | UUGU | 4290 | GACA | 3329 | UAUA | 4857 | UCAC | 266 |
| 65 | UUAU | 1148 | UUUA | 3727 | GUAC | 4284 | UAUA | 3234 | GCCA | 4750 | UAUA | 260 |
| 66 | GUUG | 1138 | UGAC | 3631 | GUAG | 3849 | UAGA | 3042 | CACA | 4659 | GUGG | 254 |
| 67 | GCAG | 1126 | UCAU | 3581 | UCCA | 3840 | GUUC | 3023 | GGUC | 4484 | UGUC | 248 |

| | | | | | | | | | | | | |
|---|---|---|---|---|---|---|---|---|---|---|---|---|
| 68 | CAAA | 1101 | UCGU | 3494 | GACA | 3751 | GGAA | 2959 | CGUA | 4348 | UGCA | 247 |
| 69 | UUCA | 1075 | UGGA | 3478 | UGAU | 3751 | GAUA | 2913 | CGUC | 4264 | UCUA | 241 |
| 70 | UAGA | 1061 | UGCA | 3462 | UACA | 3718 | UCAU | 2903 | CUCC | 4235 | UUGU | 233 |
| 71 | CCGA | 1040 | UUAC | 3429 | GUUC | 3700 | GUGG | 2853 | CUUA | 4213 | GCUA | 233 |
| 72 | UGUU | 1037 | GUAA | 3354 | GUAA | 3603 | UUGU | 2824 | UUGC | 4134 | UUCU | 232 |
| 73 | UGGA | 1000 | GGCA | 3348 | UCAU | 3596 | UUAU | 2751 | CGAC | 4125 | GAUA | 231 |
| 74 | UUGU | 997 | UCCA | 3347 | GGUA | 3465 | UGUU | 2628 | GUGC | 4061 | UUGA | 226 |
| 75 | UCCA | 993 | UAGA | 3311 | UGAA | 3462 | UGAU | 2610 | CUGA | 4052 | GUAC | 223 |
| 76 | CUGC | 981 | UCCU | 3251 | UAAA | 3450 | CUCA | 2603 | UGGG | 4030 | UGGA | 221 |
| 77 | CGAC | 980 | UCAA | 3242 | UGGC | 3446 | GCUA | 2582 | UAAG | 3890 | GUCG | 218 |
| 78 | GGUG | 965 | UGGU | 3202 | GUCC | 3371 | UACA | 2556 | CUAA | 3831 | UUUC | 216 |
| 79 | UAUC | 964 | UACU | 3152 | UCGU | 3274 | UUCC | 2549 | CCCA | 3828 | UAAU | 211 |
| 80 | CGAA | 951 | UACA | 2796 | UGCU | 3089 | GAGG | 2519 | GACA | 3747 | UUCC | 209 |
| 81 | GAAG | 942 | UUUC | 2724 | UGGU | 3077 | UUUC | 2392 | UUCC | 3742 | UGUU | 208 |
| 82 | GUCG | 933 | GUUC | 2620 | GCAG | 2984 | UCGU | 2375 | UAUG | 3737 | UGGU | 208 |
| 83 | CUAC | 907 | UGGC | 2589 | UUUC | 2976 | GCCA | 2369 | UCAA | 3711 | GUUG | 204 |
| 84 | CUAA | 888 | UGAA | 2557 | UUCC | 2976 | UCUU | 2367 | GUUG | 3690 | UGAU | 199 |
| 85 | GUGG | 882 | UAGU | 2458 | GCUA | 2971 | UAAU | 2350 | UGAC | 3680 | GGGG | 191 |
| 86 | UUUA | 876 | UAUC | 2428 | GCCA | 2913 | CGCA | 2323 | GAUA | 3663 | GCCA | 183 |
| 87 | GAAA | 856 | GUAG | 2360 | GCAA | 2861 | GCGG | 2322 | UCAC | 3596 | UCCA | 176 |
| 88 | GGAG | 854 | GUAC | 2359 | GAAG | 2811 | GGGG | 2276 | UUUC | 3596 | UAGU | 173 |
| 89 | CGCA | 852 | UAAU | 2322 | GGAC | 2811 | GUCG | 2248 | GUCG | 3561 | GGUU | 171 |
| 90 | UCGU | 843 | UGUC | 2230 | CUCA | 2794 | UCCU | 2235 | GCAG | 3529 | UAGA | 168 |
| 91 | CUCA | 842 | UUCC | 2201 | CGGA | 2746 | UGGU | 2217 | UGAA | 3507 | UCGA | 168 |

| | | | | | | | | | | | | |
|---|---|---|---|---|---|---|---|---|---|---|---|---|
| 92 | CGUA | 833 | GGUC | 2076 | UCUC | 2730 | GGUG | 2178 | GCUC | 3489 | GCGG | 162 |
| 93 | UGGU | 833 | GCCA | 2074 | UACC | 2711 | GUAA | 2176 | GAUG | 3487 | UCGU | 154 |
| 94 | GUCA | 822 | GUUG | 2024 | UAAU | 2694 | GUCC | 2171 | UCGC | 3463 | GACA | 153 |
| 95 | UACC | 821 | CUUC | 1980 | CUUA | 2692 | CCCA | 2118 | GUAG | 3401 | UCAU | 153 |
| 96 | GGCA | 814 | UCUC | 1975 | UAUC | 2676 | GUAC | 2117 | CCGA | 3394 | GAGG | 151 |
| 97 | CCUA | 810 | UACC | 1914 | UGCC | 2656 | UAGU | 2055 | UCCU | 3384 | UGCC | 150 |
| 98 | AUAG | 801 | CUUA | 1868 | UACU | 2638 | CUUA | 2053 | UAGA | 3365 | CUGA | 149 |
| 99 | UGAU | 792 | CUCA | 1864 | UGUC | 2611 | CACA | 2027 | CGCC | 3304 | GGAC | 149 |
| 100 | CAAC | 763 | UGCC | 1861 | GGAG | 2445 | UCAC | 2003 | CAUC | 3297 | CUUA | 148 |
| 101 | UCAU | 761 | AUUU | 1836 | CUUC | 2436 | UGCU | 1988 | UGUC | 3294 | CUAA | 148 |
| 102 | UCUU | 755 | CUAC | 1820 | CUAA | 2427 | UCCC | 1965 | UAAC | 3256 | AGUA | 147 |
| 103 | UCUC | 754 | UUAA | 1775 | CUAC | 2424 | AUUA | 1959 | AUGA | 3210 | GGCC | 147 |
| 104 | CCAC | 739 | GACA | 1759 | UAGU | 2384 | AGUA | 1956 | UGCC | 3185 | CCGA | 141 |
| 105 | UGAA | 713 | GUCC | 1727 | GGUC | 2362 | GUUG | 1932 | GCUG | 3168 | GAUC | 140 |
| 106 | CACA | 703 | UAAA | 1706 | UGUU | 2301 | GGUC | 1887 | GGGG | 3154 | GGGC | 137 |
| 107 | UAUU | 699 | GGUG | 1565 | CCCA | 2257 | CUCC | 1870 | GGCG | 3064 | GCCG | 136 |
| 108 | CAGC | 692 | CUAA | 1557 | CUCC | 2148 | UCUC | 1829 | AGUA | 3050 | UCUU | 134 |
| 109 | CCCA | 672 | GGAG | 1545 | GGGG | 2145 | CGUA | 1828 | GGUG | 3041 | CGUA | 132 |
| 110 | CCAA | 661 | UCCC | 1526 | CGAC | 2142 | CGGA | 1806 | GAUC | 2991 | CUCA | 131 |
| 111 | UCAA | 658 | CCUA | 1487 | UAUU | 2092 | UGUC | 1791 | UUGU | 2985 | GUCC | 130 |
| 112 | UAAU | 631 | GAUG | 1441 | CUGA | 2089 | CUGA | 1746 | UAGC | 2977 | GCUG | 128 |
| 113 | CGGC | 624 | GGAC | 1431 | AUUA | 2038 | UAUC | 1739 | CGGC | 2973 | UACA | 127 |
| 114 | CCGC | 608 | GCAG | 1419 | UCAA | 2035 | AUCA | 1738 | GCCG | 2950 | AUAG | 125 |
| 115 | AUCG | 607 | CUCC | 1367 | GUGG | 2008 | UGCC | 1733 | GGAG | 2934 | UAUC | 123 |

| | | | | | | | | | | | | | |
|---|---|---|---|---|---|---|---|---|---|---|---|---|---|
| 116 | UACU | 606 | AUCU | 1346 | CGCA | 1986 | UAUU | 1703 | CCUA | 2886 | GUAU | 120 |
| 117 | CUUC | 605 | GUGC | 1342 | CACA | 1960 | CCUA | 1625 | GGAC | 2851 | GGCG | 119 |
| 118 | CUUA | 605 | GUCG | 1294 | UCCC | 1922 | GUCU | 1562 | UGGC | 2840 | AUUA | 118 |
| 119 | GGCG | 580 | CGUC | 1250 | CAUC | 1881 | UAAA | 1562 | CGAA | 2839 | GUGC | 115 |
| 120 | CAUA | 579 | CGAC | 1238 | AUAA | 1866 | CUUC | 1544 | GGCC | 2825 | GUGU | 114 |
| 121 | UCCC | 567 | GAAG | 1226 | CGUC | 1843 | UACC | 1509 | GCGG | 2823 | CUUC | 113 |
| 122 | GUAA | 563 | CGCA | 1208 | CGUA | 1812 | CAUA | 1495 | CAAC | 2798 | UUAA | 111 |
| 123 | GCAA | 550 | AUUA | 1205 | CAAC | 1804 | AUGA | 1484 | UCUC | 2786 | CGCA | 110 |
| 124 | GACA | 542 | GUGG | 1203 | GUGC | 1769 | CUAC | 1480 | GUGU | 2763 | CAGA | 109 |
| 125 | CUCC | 533 | CAUC | 1174 | AUCA | 1738 | CAGA | 1476 | UCCC | 2714 | UAUU | 105 |
| 126 | CGUC | 522 | CCCA | 1173 | AGGA | 1737 | UGAA | 1476 | UGCU | 2706 | UCUC | 104 |
| 127 | UCCU | 522 | CCAC | 1173 | CAAA | 1707 | CCCC | 1427 | UCGU | 2665 | CGGA | 102 |
| 128 | UAAA | 506 | CCGA | 1164 | CUGC | 1672 | AUAG | 1398 | CCUC | 2617 | CGUC | 102 |
| 129 | UAGU | 505 | CUGA | 1160 | AGUA | 1669 | AUUU | 1384 | UACC | 2563 | GAUG | 101 |
| 130 | GUUA | 500 | CCAA | 1154 | GUGU | 1668 | CUAA | 1357 | CACC | 2555 | AGUC | 100 |
| 131 | GUAC | 494 | AUAU | 1149 | GAGG | 1656 | CCGA | 1345 | UUAA | 2525 | AUCA | 97 |
| 132 | GGGG | 478 | CUGC | 1121 | CCAC | 1644 | CAAA | 1341 | CCGC | 2521 | GCAU | 97 |
| 133 | CUGU | 470 | AUAA | 1108 | CACC | 1609 | GCAU | 1326 | CUCU | 2489 | CAUA | 97 |
| 134 | GAUA | 460 | AUCA | 1108 | CCGA | 1596 | GCUG | 1296 | CCCC | 2462 | CCCA | 95 |
| 135 | CGCC | 450 | CCUC | 1093 | CCUA | 1589 | GGCG | 1283 | CCAC | 2458 | AUGA | 95 |
| 136 | GUUC | 436 | CUGU | 1070 | GGCC | 1587 | UACU | 1280 | GAAG | 2455 | GGAU | 94 |
| 137 | GCUG | 435 | AGUU | 1069 | CUGU | 1553 | GGAC | 1271 | UAUC | 2453 | AUAA | 90 |
| 138 | UUAA | 430 | GUAU | 1061 | CCCC | 1521 | GUGC | 1261 | UAAA | 2451 | CCAA | 88 |
| 139 | GAUG | 424 | CGGA | 1046 | GAAC | 1507 | AUAU | 1257 | GCCU | 2441 | AGGA | 88 |

| | | | | | | | | | | | | |
|---|---|---|---|---|---|---|---|---|---|---|---|---|
| 140 | GCGG | 422 | GGUU | 1038 | AUAU | 1479 | CGAC | 1250 | GCGC | 2435 | UCCC | 87 |
| 141 | CAUC | 416 | CGUA | 1023 | GAUC | 1476 | GCAA | 1246 | GCAC | 2434 | CGAA | 86 |
| 142 | GAGG | 405 | CAUA | 1017 | GAUA | 1476 | GUAU | 1208 | UUCU | 2414 | GCAC | 85 |
| 143 | GUCC | 403 | CCGC | 1014 | CAGA | 1447 | CAUC | 1207 | GGGC | 2404 | UGCU | 83 |
| 144 | CCUC | 370 | CACA | 1012 | GUCG | 1444 | UUAA | 1205 | CCCU | 2394 | GGGU | 81 |
| 145 | GGAC | 368 | GUGU | 992 | AUGA | 1439 | AAUA | 1199 | GCCC | 2332 | UGAA | 81 |
| 146 | GACG | 367 | GCUC | 991 | AUUU | 1424 | AGGA | 1197 | AGGA | 2327 | CUCC | 80 |
| 147 | GCUA | 349 | GAUC | 983 | CGCC | 1405 | GAUG | 1192 | UGGU | 2322 | UCAA | 77 |
| 148 | CUAU | 347 | GUCU | 967 | GCAC | 1384 | AGCA | 1191 | CGCU | 2310 | CUAC | 75 |
| 149 | GGUC | 345 | AUAG | 965 | GCGG | 1340 | CACC | 1159 | CAAA | 2294 | UCCU | 72 |
| 150 | GUGC | 344 | AGUA | 960 | CCUC | 1312 | GACG | 1150 | UUUU | 2276 | GCUC | 71 |
| 151 | CACC | 343 | AUAC | 949 | GCGC | 1285 | AUCU | 1132 | CUGU | 2241 | GACG | 70 |
| 152 | AGUA | 337 | CGCC | 938 | ACUA | 1253 | CGUC | 1124 | CAGC | 2229 | CACA | 69 |
| 153 | CCCC | 331 | CACC | 922 | GUUG | 1251 | CUGU | 1096 | GCGU | 2222 | GUUU | 69 |
| 154 | AUGA | 325 | GGCC | 908 | UCUU | 1243 | GGCC | 1086 | CAGA | 2149 | CCUA | 68 |
| 155 | AUUG | 323 | GCAC | 896 | CAUA | 1238 | CCUC | 1082 | CGGU | 2149 | GUCU | 67 |
| 156 | AUAC | 317 | GGAU | 890 | CGGC | 1225 | GUGU | 1051 | AUCA | 2102 | CAAA | 67 |
| 157 | CGGU | 306 | AUGA | 887 | AGCA | 1194 | ACUA | 1030 | GGCU | 2080 | AGCA | 67 |
| 158 | AUCA | 300 | GCUG | 884 | CGAA | 1174 | CGCC | 1029 | GAGG | 2079 | UACC | 66 |
| 159 | GUAU | 298 | GGCG | 850 | AUAG | 1145 | GUUU | 1021 | AGCA | 2074 | AAUA | 66 |
| 160 | AGGA | 287 | CGGC | 849 | GACC | 1135 | ACGA | 1020 | GUCU | 2051 | AUCG | 66 |
| 161 | AUCU | 284 | CAAC | 840 | CAGC | 1128 | CAAC | 1012 | UCAU | 2013 | CUGC | 64 |
| 162 | AGAG | 268 | GUUU | 840 | AUCU | 1127 | GAAU | 1006 | CCGU | 2006 | CUUU | 63 |
| 163 | AUAU | 261 | CCCC | 820 | GGCG | 1099 | GAUC | 991 | AUUA | 1945 | CUGU | 59 |

| | | | | | | | | | | | | |
|---|---|---|---|---|---|---|---|---|---|---|---|---|
| 164 | GUGU | 253 | ACUU | 812 | CCGC | 1084 | CUGC | 977 | AUUU | 1945 | AUAU | 59 |
| 165 | ACAG | 246 | CGAA | 808 | GAUG | 1080 | GCUC | 962 | ACUA | 1917 | AGAA | 58 |
| 166 | CUCU | 244 | GGGG | 802 | GGGC | 1067 | CAGC | 937 | UGAU | 1866 | GCGU | 58 |
| 167 | CGAU | 242 | GAGG | 793 | ACGA | 1054 | AUGU | 912 | GACC | 1842 | AUUG | 55 |
| 168 | CGUU | 242 | CAGA | 788 | AUAC | 1043 | CUCU | 901 | GACG | 1824 | AUGG | 54 |
| 169 | GCCA | 241 | CGGU | 777 | CGGU | 1019 | AUCG | 899 | AUUC | 1820 | CCGU | 53 |
| 170 | AGCA | 239 | CUUU | 777 | GUCU | 965 | AGUU | 891 | ACGA | 1779 | UAAA | 53 |
| 171 | AUUA | 239 | GCAU | 767 | GGAU | 953 | UCAA | 879 | AUAU | 1768 | GCGC | 52 |
| 172 | GGUU | 239 | CAGC | 767 | AAAA | 944 | CGGC | 859 | UAGU | 1746 | CUCU | 52 |
| 173 | GCCG | 236 | CUCU | 763 | GUAU | 933 | CGAA | 847 | GGGU | 1732 | CCUC | 51 |
| 174 | AUUU | 232 | AGAA | 762 | GCCC | 914 | CUUU | 839 | UACU | 1713 | CGAC | 50 |
| 175 | CCCU | 231 | CCGU | 749 | GACG | 912 | GGGC | 835 | AUCU | 1703 | AGUG | 50 |
| 176 | AUGU | 214 | GCUU | 747 | CCCU | 908 | ACCA | 831 | AUAG | 1702 | GAAU | 50 |
| 177 | AUGG | 213 | CCUU | 738 | ACAA | 894 | GCCG | 818 | ACAA | 1673 | CGGC | 50 |
| 178 | ACGA | 210 | GGGU | 729 | CUCU | 890 | GCCC | 803 | AUGG | 1659 | AUUU | 50 |
| 179 | CUUU | 208 | GCGU | 726 | CUUU | 886 | AUAC | 800 | AUCG | 1659 | CCCC | 49 |
| 180 | CCAU | 207 | GGGC | 719 | AGAA | 884 | GAAC | 782 | UGUU | 1652 | AUCU | 48 |
| 181 | GGGC | 206 | AUGU | 715 | AAUA | 877 | GCGU | 774 | AUGC | 1644 | GAAC | 48 |
| 182 | CCUU | 206 | GCGG | 715 | ACCA | 870 | CCGC | 764 | GCAU | 1620 | CCGC | 46 |
| 183 | AUUC | 204 | AGGA | 700 | GCAU | 857 | GCGC | 751 | CAUA | 1607 | AGUU | 46 |
| 184 | ACUA | 204 | CUAU | 696 | GCGU | 853 | GGAU | 751 | ACCA | 1604 | AUGU | 46 |
| 185 | GUCU | 204 | GAAC | 695 | CCGU | 829 | GCCU | 747 | AUAC | 1587 | AAGA | 45 |
| 186 | CCGU | 199 | GAAU | 690 | GGUG | 817 | CGGU | 739 | AUUG | 1573 | CUAU | 45 |
| 187 | GUUU | 194 | ACGA | 682 | GCUG | 817 | CCCU | 737 | GAGC | 1477 | CGCC | 45 |

| | | | | | | | | | | | | |
|---|---|---|---|---|---|---|---|---|---|---|---|---|
| 188 | CGCU | 194 | AGCA | 680 | GCUC | 816 | GGGU | 732 | GAAC | 1475 | CGGU | 44 |
| 189 | CAGU | 190 | CAAA | 676 | AUGU | 806 | AUUG | 730 | AGUC | 1468 | GAGC | 44 |
| 190 | CACU | 189 | GAGU | 663 | GGGU | 800 | CCGU | 718 | GGUU | 1455 | GCCU | 44 |
| 191 | GGGU | 187 | ACUA | 660 | ACCU | 769 | GACC | 714 | ACUG | 1424 | GAGU | 43 |
| 192 | AGAA | 184 | GAUU | 655 | GAAU | 764 | CUAU | 687 | CACU | 1418 | GAUU | 43 |
| 193 | GGCC | 184 | AUUG | 648 | AUCG | 755 | CCUU | 681 | CUAU | 1400 | GACC | 42 |
| 194 | AGAC | 183 | AAUA | 635 | CUAU | 755 | ACUU | 680 | GUAU | 1392 | ACCA | 42 |
| 195 | AGCG | 182 | GCCC | 600 | GUUU | 725 | GGUU | 674 | AUCC | 1372 | ACUA | 42 |
| 196 | AGUU | 182 | ACAA | 593 | GAGC | 715 | CGUU | 673 | UAAU | 1370 | CCAC | 41 |
| 197 | AUGC | 179 | GGCU | 588 | AACA | 702 | AGAA | 665 | CUUU | 1356 | CCUU | 40 |
| 198 | AUCC | 176 | CAUU | 581 | GCCU | 688 | AUAA | 662 | GGAU | 1347 | GGCU | 39 |
| 199 | GGAU | 174 | AUCG | 574 | GAGU | 688 | GAGU | 649 | ACCU | 1328 | CACC | 39 |
| 200 | AGUG | 173 | CAGU | 573 | GGCU | 634 | AAGA | 634 | ACCG | 1320 | AUGC | 38 |
| 201 | CAAU | 172 | GACC | 550 | ACAU | 626 | CAGU | 624 | ACGG | 1302 | GCCC | 38 |
| 202 | ACUU | 167 | GCGC | 547 | AUUG | 590 | CCAU | 616 | CCAU | 1250 | AUAC | 37 |
| 203 | ACUG | 166 | AAUU | 526 | AUUC | 589 | CGAU | 611 | AGUG | 1241 | CAUC | 37 |
| 204 | CAUU | 163 | CCCU | 494 | AAGA | 589 | CGCU | 610 | AGCG | 1238 | AGAG | 36 |
| 205 | GCGC | 160 | ACAU | 484 | CCAA | 584 | AUUC | 600 | CGUU | 1218 | ACAA | 35 |
| 206 | AAUA | 157 | CGUU | 476 | AGAU | 578 | CACU | 587 | ACUC | 1210 | AUUC | 34 |
| 207 | AUAA | 156 | AGGU | 476 | GCCG | 567 | AACA | 580 | GUUU | 1206 | AAUU | 33 |
| 208 | GCUC | 154 | CCAU | 473 | CAGU | 553 | AAAA | 574 | GCUU | 1168 | ACAG | 33 |
| 209 | ACAC | 153 | GCCU | 472 | ACAC | 527 | AGUG | 570 | ACAG | 1165 | CAUU | 33 |
| 210 | AGAU | 153 | ACCU | 471 | AGAG | 526 | AUCC | 569 | CCAA | 1160 | AGGG | 33 |
| 211 | GCAC | 151 | CACU | 470 | AGGU | 520 | AUGC | 559 | UUAU | 1152 | UACU | 33 |

| | | | | | | | | | | | | |
|---|---|---|---|---|---|---|---|---|---|---|---|---|
| 212 | AACA | 147 | AGAU | 467 | AUGC | 512 | CCAA | 542 | ACGC | 1150 | ACGA | 33 |
| 213 | ACCA | 147 | AUGC | 465 | AUCC | 505 | AUGG | 537 | AUGU | 1120 | AAAA | 32 |
| 214 | AGUC | 140 | GAGC | 441 | CACU | 503 | GAGC | 525 | ACCC | 1108 | CGUU | 32 |
| 215 | GAAC | 139 | AUUC | 432 | AGCU | 501 | GGCU | 523 | UCUU | 1091 | CAGC | 31 |
| 216 | GCAU | 139 | GCCG | 429 | CGAU | 487 | CAUU | 507 | GAGU | 1086 | GACU | 31 |
| 217 | GCGU | 139 | ACCA | 408 | ACGU | 482 | ACAG | 497 | CGAU | 1082 | ACCG | 30 |
| 218 | AGGU | 138 | AGCU | 403 | CCAU | 473 | ACAU | 486 | CCUU | 1021 | GCUU | 30 |
| 219 | GAUC | 136 | AGUC | 388 | CGCU | 471 | CCAC | 485 | AGGG | 996 | AAUG | 30 |
| 220 | AACG | 133 | AGAG | 384 | ACAG | 464 | ACGU | 481 | AAUA | 985 | CAAC | 29 |
| 221 | ACAU | 127 | CGCU | 375 | AGUU | 446 | AAUU | 469 | AGAG | 982 | CCCU | 28 |
| 222 | AAGA | 125 | ACAC | 374 | AGAC | 444 | AGGU | 463 | AGAA | 977 | AGAU | 27 |
| 223 | GAGU | 123 | GACG | 366 | CAAU | 438 | AGAG | 463 | AAAA | 974 | AGGU | 27 |
| 224 | AGCU | 122 | ACAG | 351 | GGUU | 432 | ACCC | 455 | UAUU | 974 | ACUG | 26 |
| 225 | ACCG | 121 | AGAC | 340 | AGUC | 428 | AGAU | 438 | ACGU | 973 | AAGG | 26 |
| 226 | GGCU | 120 | ACGU | 335 | GACU | 411 | AGCG | 434 | AGGC | 969 | CGAU | 25 |
| 227 | AAUG | 119 | AUCC | 331 | AUGG | 410 | ACCU | 432 | AGCC | 962 | AGCU | 24 |
| 228 | GCUU | 118 | AAAA | 326 | ACUU | 386 | ACCG | 421 | AGGU | 949 | CAGU | 24 |
| 229 | GAAU | 111 | GACU | 324 | AGCG | 375 | CAAU | 420 | AGCU | 941 | AUCC | 24 |
| 230 | AAAG | 104 | CGAU | 306 | ACGC | 363 | AGCU | 416 | GACU | 934 | ACUU | 23 |
| 231 | GACC | 104 | AACA | 300 | CGUU | 350 | GCUU | 407 | ACAU | 918 | ACAU | 23 |
| 232 | ACGC | 103 | AAGA | 300 | ACUG | 340 | GAUU | 398 | AACA | 909 | ACAC | 22 |
| 233 | AGGG | 103 | AUGG | 292 | CAUU | 336 | ACUG | 387 | ACAC | 886 | AGCG | 22 |
| 234 | ACAA | 103 | AGUG | 275 | ACGG | 309 | GACU | 386 | CAGU | 886 | CCAU | 22 |
| 235 | ACGU | 100 | ACGC | 249 | AGGC | 301 | ACGC | 377 | ACUU | 808 | CACU | 21 |

| | | | | | | | | | | | | |
|---|---|---|---|---|---|---|---|---|---|---|---|---|
| 236 | GAGC | 98 | CAAU | 248 | AGGG | 291 | AGUC | 351 | AGUU | 781 | AACA | 21 |
| 237 | GAUU | 97 | AAUG | 247 | AGUG | 285 | ACAC | 333 | AGAC | 752 | CAAU | 21 |
| 238 | AGGC | 92 | AGCG | 245 | AACU | 284 | GCAC | 333 | AUAA | 748 | ACCC | 18 |
| 239 | ACUC | 92 | ACUG | 233 | AAAU | 280 | ACAA | 322 | AAUG | 745 | ACGG | 18 |
| 240 | GCCC | 85 | AGGC | 231 | GAUU | 276 | AGAC | 322 | AGAU | 744 | ACGU | 17 |
| 241 | AAUU | 83 | AACU | 223 | ACCG | 268 | AGGG | 317 | AAGA | 709 | AAAG | 17 |
| 242 | AACU | 81 | ACCG | 218 | GCUU | 258 | AAUG | 316 | GAAU | 704 | AGCC | 17 |
| 243 | AAAA | 80 | AGGG | 206 | AGCC | 257 | ACUC | 302 | AAUC | 623 | AGAC | 17 |
| 244 | ACGG | 77 | ACUC | 201 | ACUC | 256 | AACU | 301 | GAUU | 603 | AAUC | 16 |
| 245 | GACU | 76 | AGCC | 199 | AAUC | 252 | ACGG | 299 | CAUU | 577 | AGGC | 14 |
| 246 | AGCC | 74 | AAGU | 194 | ACCC | 250 | AGCC | 290 | AACG | 551 | AAGC | 13 |
| 247 | GCCU | 72 | AAUC | 175 | AACG | 246 | AGGC | 286 | AAAG | 475 | ACCU | 13 |
| 248 | ACCU | 70 | AAAU | 170 | AAUU | 245 | AACG | 264 | CAAU | 474 | AAAU | 12 |
| 249 | AAAC | 65 | ACGG | 167 | AAGU | 230 | AACC | 238 | AACC | 438 | CGCU | 12 |
| 250 | ACCC | 65 | ACCC | 162 | AAAC | 222 | AAAU | 234 | AAGG | 407 | ACGC | 11 |
| 251 | AAUC | 63 | AAAC | 142 | AAUG | 219 | AAAG | 232 | AAAU | 386 | AAGU | 11 |
| 252 | AAAU | 55 | AAGC | 129 | AAAG | 195 | AAUC | 215 | AACU | 366 | ACUC | 11 |
| 253 | AAGC | 49 | AAAG | 128 | CCUU | 181 | AAAC | 210 | AAGC | 363 | AACG | 11 |
| 254 | AAGU | 49 | AACG | 124 | AAGC | 164 | AAGU | 188 | AAUU | 339 | AACU | 11 |
| 255 | AAGG | 46 | AACC | 113 | AACC | 163 | AAGC | 169 | AAGU | 329 | AACC | 8 |
| 256 | AACC | 45 | AAGG | 92 | AAGG | 139 | AAGG | 156 | AAAC | 303 | AAAC | 7 |
| Frequency Range | 130 | | 111 | | 120 | | 85 | | 56 | | 278 | |

**Table S6. Comparison of the top 40 sequences of loop-closing product P1 from reactions at 250 nM and 5 nM duplex A:a concentration.** Newly appeared sequences in the 250 nM reactions comparing to the 5 nM ones are highlighted in red.

| | X:Y = C:G Reaction concentration at | | X:Y = G:C Reaction concentration at | | X:Y = A:U Reaction concentration at | | X:Y = U:A Reaction concentration at | |
|---|---|---|---|---|---|---|---|---|
| Rank | 250 nM | 5 nM | 250 nM | 5 nM | 250 nM | 5 nM | 250 nM | 5 nM |
| 1 | UCAG | UGCG | UUGG | UCUG | GAGA | GAGA | GAGA | GGGA |
| 2 | UUAG | UACG | UCAG | UCCG | GUGA | GGGA | UACG | GAGA |
| 3 | UUGG | UGUG | GUGA | GAGA | GGGA | GUGA | GGGA | GUGA |
| 4 | UGAG | UUCG | UACG | UCAG | GCGA | GCGA | UUCG | UCCG |
| 5 | UGCG | UUUG | UGAG | UACG | UUGG | CUCG | GUGA | UUCG |
| 6 | UACG | UGAG | GUGG | GUGA | CUCG | UUAG | GCGA | UGCG |
| 7 | UUUG | UAUG | GAGA | UGAG | UUAG | UGCG | UUUG | GCGA |
| 8 | UCUG | UUAG | UUUG | UGCG | CGCG | UGAG | UCCG | UUUG |
| 9 | UUCG | UAAG | UUCG | GGGA | UGAG | CUGG | UUAG | UCUG |
| 10 | UUGC | UCAG | UCUG | UAUG | CACG | CGCG | UGCG | UUAG |
| 11 | UAUG | UCCG | UAAG | UAAG | UGCG | CUAG | UCAG | CCUG |
| 12 | UAAG | UUGG | UUAG | UGUG | CUAG | UACG | UCUG | UUGG |
| 13 | UCGG | CAGG | GCGA | UUAG | CCCC | CGAG | UGAG | UGAG |
| 14 | UGUG | UAGC | CUCC | UUCG | CCCG | UUUA | UUGG | UGUG |
| 15 | UCGC | UUGC | CUGC | UUGG | UCGG | CCCG | CACG | CAUG |
| 16 | UGGG | CGUG | UAGG | GCGA | UAGG | UUUG | CUCG | UCAG |
| 17 | UAGG | CUGG | UAUG | UAGG | CUGG | CUUG | UCGG | UACG |
| 18 | UCAC | CCUG | UCCG | UCGG | CGAG | UUCG | UAUG | UUUA |
| 19 | UCCG | GGGA | CCCU | CCGG | CUCC | CAGG | CGCG | CACG |

| | | | | | | | | |
|---|---|---|---|---|---|---|---|---|
| 20 | UAGC | UCGC | CUCG | UCUA | UCAG | CAAG | UGUG | CAGG |
| 21 | CUGG | UAAC | UGCG | CCUG | UAAG | UUGG | CCUG | CUGG |
| 22 | UGAC | CGAG | CUGG | UUUG | UGGG | CACG | UAAG | CGUG |
| 23 | CGGG | CAAG | UGUG | CGGG | UUCG | UAAG | UGGG | CCCG |
| 24 | CCGG | UGGC | CCUC | CUCG | CUUG | UGUG | CCGG | CCGG |
| 25 | UGGC | CAUG | CCUG | GAAA | UACG | UCAG | UCGC | CGGG |
| 26 | UAAC | UAGG | UCGG | UUCU | CAGG | CGGG | CUGG | UCGG |
| 27 | GGGA | CGGG | GCUA | CUGG | UUUG | UUGA | CAGG | CUUG |
| 28 | CCCG | CACG | CACG | CCCG | CGGG | GAAA | UUGC | GAAA |
| 29 | CAGG | CGCG | GGGA | UUGA | CAAG | CCAG | CAUG | UAUG |
| 30 | GGCG | UCGG | UGGG | CACG | CCCA | CGUG | GAAA | CGCG |
| 31 | GCGG | UCUG | CGCG | CCAG | UUGA | UUCA | GCGG | CAAG |
| 32 | GCCG | CUCG | CUAG | GGAA | UCCG | UCCG | GAAG | CCAG |
| 33 | GGGG | CUAG | CUUG | UCUU | CCGG | CAUG | UAGG | CGAG |
| 34 | UUAC | CCCG | GCUG | UGGG | CCAG | CCUG | GUAG | CUCG |
| 35 | CGCG | GUGA | CGGG | CUUG | GCCC | UAGG | CUUG | UAAG |
| 36 | CGUG | CCAG | GUCG | UUUU | GUCA | UGGG | CGAG | GUAG |
| 37 | CGAG | UGGG | UCCC | CAGG | GAAA | CCGG | CCCG | UUUU |
| 38 | CCUG | UGAC | CGAG | CUAG | UUUA | UCUG | CCCU | UGGG |
| 39 | UGCC | UUAC | CCGG | CGUG | UUCA | UCGG | CUAG | UUGC |
| 40 | CCGA | CCGG | UUGC | GCUA | GCGG | UAUG | CAAG | UCGC |

**Table S7. Top 40 and bottom 40 sequences of splint ligation product P2 from reactions at 250 nM duplex A:a concentration.** Sequences having less than 3 Cs or Gs among the top 40, and having less than 3 As or Us among the bottom 40s, are highlighted.

| Rank order | X:Y = C:G | Reads | X:Y = G:C | Reads | X:Y = A:U | Reads | X:Y = U:A | Reads | X:Y = G:U | Reads |
|---|---|---|---|---|---|---|---|---|---|---|
| 1 | CCCC | 4501 | CCCC | 3643 | CCCC | 4246 | CCCC | 5182 | GCCG | 4007 |
| 2 | GCGG | 4371 | GCCC | 3305 | GCCC | 4226 | GCGG | 4993 | GCGG | 3841 |
| 3 | CUCC | 4153 | GCGG | 3011 | GCGG | 4214 | GCCC | 4900 | GCCC | 3517 |
| 4 | GCCC | 4075 | CCGC | 2960 | GGGG | 3892 | GGGG | 4453 | GCCA | 3385 |
| 5 | UGCC | 4039 | CCCA | 2945 | GGCG | 3761 | GCCG | 4313 | GGGG | 3285 |
| 6 | GCCG | 3834 | CGCC | 2904 | GCCG | 3569 | GGCG | 4253 | CCGG | 3220 |
| 7 | CCGC | 3796 | GGGG | 2803 | CUCC | 3343 | CUCC | 4152 | GGCG | 3115 |
| 8 | GGCG | 3786 | CCGG | 2784 | UGGG | 3228 | CCGC | 4081 | CCCC | 2969 |
| 9 | CCCA | 3588 | CUCC | 2687 | CCGC | 3216 | GGCC | 4077 | GCGC | 2949 |
| 10 | CCCU | 3518 | GCGC | 2638 | CCCA | 3110 | CCGG | 4045 | GCAG | 2924 |
| 11 | UCCC | 3502 | GCCG | 2610 | CCGG | 3030 | CCCA | 3998 | CCCA | 2801 |
| 12 | UGGG | 3489 | GGCC | 2515 | CGCC | 2965 | CGCC | 3810 | CCCG | 2718 |
| 13 | GGGG | 3476 | CCCG | 2477 | GGCC | 2876 | CCUC | 3523 | GGCC | 2648 |
| 14 | CGCC | 3384 | GGCG | 2475 | CCCG | 2874 | UGGG | 3482 | GUGG | 2624 |
| 15 | CCGG | 3334 | UGCC | 2419 | GUGG | 2842 | GCGC | 3479 | UCGG | 2616 |
| 16 | CCUC | 3296 | CCGA | 2414 | UGCC | 2815 | CCCG | 3251 | UGCG | 2488 |
| 17 | CUGC | 3144 | UGGG | 2379 | GCGC | 2792 | GCCA | 3236 | GGCA | 2438 |
| 18 | UGGC | 2943 | CGGC | 2377 | CCUC | 2693 | UCGG | 3221 | GCUG | 2387 |

| | | | | | | | | | | |
|---|---|---|---|---|---|---|---|---|---|---|
| 19 | CCGU | 2927 | CUGC | 2319 | CCCU | 2596 | CUGC | 3180 | UCCG | 2377 |
| 20 | UCGG | 2742 | CCUC | 2256 | CCGA | 2436 | UGCC | 3139 | GGGC | 2342 |
| 21 | CCGA | 2697 | UCCC | 2227 | GCCA | 2429 | CCGA | 3091 | UGGG | 2281 |
| 22 | GGCC | 2626 | UGGC | 2162 | UCCC | 2425 | CCCU | 3084 | CGCC | 2245 |
| 23 | UCCG | 2606 | UCGG | 2157 | UCGG | 2404 | GGGC | 2884 | GGGA | 2230 |
| 24 | CCCG | 2519 | GGGC | 2143 | UCCG | 2313 | GUGG | 2840 | CCGC | 2195 |
| 25 | GUGG | 2404 | CACC | 2102 | CCGU | 2258 | CCGU | 2812 | GCGA | 2169 |
| 26 | GCGC | 2397 | GCCA | 2016 | CUGC | 2245 | GCAG | 2756 | GAGG | 2123 |
| 27 | UGCG | 2384 | GUGG | 1966 | CGGC | 2228 | UCCC | 2713 | CCGA | 2121 |
| 28 | CGGC | 2303 | CCGU | 1963 | GGGC | 2191 | UGCG | 2671 | GGAG | 2066 |
| 29 | CGCU | 2237 | CGGU | 1926 | UGGC | 2134 | UCCG | 2606 | ACCG | 1910 |
| 30 | CGGU | 2139 | GCAG | 1886 | UGCG | 2014 | GGCA | 2538 | CGGG | 1874 |
| 31 | CACC | 2115 | CCCU | 1756 | GCUG | 2007 | GCUG | 2444 | GGUG | 1811 |
| 32 | CGGG | 2109 | CCAC | 1701 | GCAG | 1876 | ACGG | 2414 | GCAC | 1797 |
| 33 | GCCA | 2092 | UCCG | 1593 | CACC | 1861 | CGGC | 2414 | ACGG | 1771 |
| 34 | CCAC | 1957 | UGCG | 1560 | CCAC | 1820 | GUCG | 2341 | CUCC | 1707 |
| 35 | CGUC | 1917 | CGUC | 1547 | CGGU | 1721 | UGGC | 2334 | GUCG | 1699 |
| 36 | CCUA | 1846 | CGGG | 1532 | GGCA | 1714 | ACCG | 2330 | CCCU | 1695 |
| 37 | GGGC | 1662 | GGCA | 1527 | CGCU | 1714 | CACC | 2328 | UGCC | 1671 |
| 38 | GCAG | 1580 | CGCU | 1476 | GUCG | 1649 | CCAC | 2314 | GACG | 1620 |
| 39 | GCUG | 1566 | GAGG | 1458 | CCUA | 1638 | CCUA | 2288 | UCCC | 1528 |
| 40 | GGCA | 1558 | CGGA | 1435 | CGGG | 1586 | GGUG | 2176 | CCUG | 1498 |
| 217 | GUAA | 8 | CAAA | 15 | UAAC | 11 | CAAU | 17 | UAAC | 13 |
| 218 | GAAC | 8 | CUAG | 15 | CAAA | 10 | UUCU | 16 | ACAA | 12 |

| | | | | | | | | | | |
|---|---|---|---|---|---|---|---|---|---|---|
| 219 | UUUC | 8 | CAAG | 13 | AAGC | 9 | CAAA | 15 | AAUG | 11 |
| 220 | GUUA | 8 | ACAU | 12 | UUUC | 9 | AUCU | 15 | CAAU | 10 |
| 221 | ACAA | 6 | AUGU | 11 | AAUG | 9 | UUUC | 14 | AACA | 9 |
| 222 | AAUG | 6 | UAUA | 9 | ACAA | 9 | UAAC | 13 | GAAA | 9 |
| 223 | UAUA | 6 | ACAA | 9 | ACUU | 8 | UGAA | 11 | AUGU | 8 |
| 224 | CAAA | 5 | AUUC | 9 | UAUA | 8 | AACA | 11 | CUUU | 8 |
| 225 | AACA | 5 | AACA | 9 | ACAU | 7 | AAGA | 10 | GAAU | 8 |
| 226 | AAAG | 5 | CUUU | 8 | GUUU | 7 | AUUC | 10 | AUUC | 8 |
| 227 | UUAU | 5 | GAUU | 7 | AACU | 7 | AUUA | 9 | CAUU | 8 |
| 228 | AUGU | 4 | AGAU | 7 | AAAG | 6 | AUGU | 9 | AUCU | 7 |
| 229 | AAAC | 3 | AGAA | 6 | AUGU | 6 | GAUU | 7 | UUUC | 7 |
| 230 | ACAU | 3 | ACUU | 6 | UUAU | 6 | UUUU | 7 | ACUU | 6 |
| 231 | ACUU | 3 | UUAU | 5 | UAAU | 5 | ACUU | 7 | AAGA | 6 |
| 232 | AUUC | 3 | AAGA | 5 | GAAA | 5 | AGUU | 6 | AGUU | 6 |
| 233 | AGAU | 3 | GAAA | 4 | AGUU | 5 | AGAA | 6 | CAAA | 6 |
| 234 | UAAU | 3 | AGUU | 4 | AUUC | 5 | UAUA | 6 | UAUA | 5 |
| 235 | UAUU | 3 | AAAG | 4 | AAGA | 4 | AAUC | 6 | AAGU | 5 |
| 236 | AAAU | 2 | AAGU | 3 | AUCU | 4 | AAAG | 4 | AGAA | 5 |
| 237 | AAUC | 2 | AUUA | 3 | AGAA | 3 | AGAU | 4 | AGAU | 4 |
| 238 | GUUU | 2 | AAUC | 3 | AAUC | 3 | UAAU | 4 | AAUA | 4 |
| 239 | AAGU | 2 | GUUU | 2 | GAUU | 3 | UUUA | 3 | AAAG | 4 |
| 240 | UUUA | 2 | AUCU | 2 | UAUU | 3 | AACU | 3 | UUUA | 4 |
| 241 | AUAU | 2 | UUAA | 2 | AGAU | 3 | GUUU | 3 | AAUC | 3 |
| 242 | GAAA | 2 | AAAC | 2 | AUAU | 3 | UAUU | 3 | UUAU | 2 |

| | | | | | | | | | | |
|---|---|---|---|---|---|---|---|---|---|---|
| 243 | AGAA | 2 | UAAU | 2 | GAAU | 2 | AUAA | 3 | UAAU | 2 |
| 244 | AUAA | 1 | AUAU | 1 | AAGU | 2 | AAGU | 2 | UUAA | 2 |
| 245 | AAAA | 1 | GAAU | 1 | UAAA | 2 | AAAU | 2 | AAAC | 1 |
| 246 | AGUU | 1 | UAUU | 1 | UUAA | 2 | GAAA | 2 | AUAU | 1 |
| 247 | UUAA | 1 | AAUA | 1 | UUUU | 2 | UUAU | 2 | AUAA | 1 |
| 248 | GAUU | 1 | AACU | 1 | AUAA | 1 | GAAU | 2 | UAUU | 1 |
| 249 | AAUU | 1 | UUUA | 1 | AAAC | 1 | AAAC | 2 | AACU | 1 |
| 250 | AAUA | 1 | AAUU | 1 | UUUA | 1 | AAUA | 1 | AAUU | 1 |
| 251 | AACU | 1 | AUAA | 0 | AAAA | 1 | UUAA | 1 | AUUA | 1 |
| 252 | UAAA | 1 | UAAA | 0 | AUUU | 1 | AUUU | 1 | UAAA | 0 |
| 253 | | | AAAU | 0 | AUUA | 1 | AAAA | 1 | AAAA | 0 |
| 254 | | | | | AAUU | 1 | | | | |
| 255 | | | | | AAUA | 1 | | | | |
| 256 | | | | | AAAU | 1 | | | | |
| Uncovered Sequence # | 4 | | 3 | | 0 | | 3 | | 3 | |

**Table S8. Biological tetraloops from bpRNA-1m(90) tabulated by different closing base-pairs, X:Y.** RNA sequences in the database with > 90% sequences similarities were removed when keeping at least 70% alignment coverage.

| Rank order | C:G closing | Counts | G:C closing | Counts | A:U closing | Counts | U:A closing | Counts | G:U closing | Counts | U:G closing | Counts |
|---|---|---|---|---|---|---|---|---|---|---|---|---|
| 1 | GAAA | 1855 | GAAA | 1433 | GAAA | 457 | GAAA | 233 | UAGG | 31 | GAGA | 823 |
| 2 | GCAA | 1753 | GCAA | 640 | GAGA | 429 | GUGA | 143 | GGAA | 24 | GAAA | 303 |
| 3 | UUCG | 1670 | GUAA | 481 | GCAA | 309 | GCAA | 95 | GAAA | 23 | UUCG | 285 |
| 4 | GUGA | 1627 | GUGA | 480 | GUGA | 253 | UAAC | 85 | UACG | 13 | UUAG | 112 |
| 5 | AGCC | 867 | CUUG | 309 | GUAA | 217 | GGAA | 75 | AAAU | 11 | UAAC | 94 |
| 6 | UACG | 855 | GCGA | 284 | UUUA | 194 | GUAA | 71 | UCAU | 9 | UAGG | 83 |
| 7 | GAAG | 771 | GGAA | 199 | AACA | 189 | UUCG | 57 | UUAA | 8 | GGAA | 74 |
| 8 | GCGA | 611 | GAGA | 190 | GCGA | 165 | GCGA | 55 | UGAU | 8 | GUGA | 66 |
| 9 | GGAA | 453 | UGAA | 171 | AGCA | 98 | GAGA | 48 | UUUA | 7 | GCAA | 66 |
| 10 | UAAC | 443 | UUCG | 96 | UCAU | 65 | GAAG | 45 | AAAG | 7 | CAGG | 54 |
| 11 | GUAA | 391 | ACGA | 85 | GAGG | 44 | GCCA | 42 | UGAG | 7 | GUAA | 53 |
| 12 | GAGA | 376 | GCAU | 82 | GGAA | 41 | UUUU | 39 | UAAU | 6 | GCAU | 38 |
| 13 | CCCC | 244 | GAAG | 67 | CUUG | 37 | AAAA | 36 | UAAA | 6 | GGGA | 37 |
| 14 | GCAU | 237 | GGGA | 56 | UUUU | 30 | GAUU | 35 | AAAC | 6 | GCGA | 34 |
| 15 | UUUU | 191 | AGAA | 55 | UUCG | 25 | AACA | 34 | GAGU | 6 | GCAG | 33 |
| 16 | UUAG | 161 | UUUA | 54 | UUAA | 23 | UUUC | 33 | AGUU | 6 | UCCU | 30 |
| 17 | CAAG | 159 | UUUU | 40 | AAAA | 20 | UUAU | 30 | UAUU | 5 | UGAG | 26 |
| 18 | GCCA | 143 | CUUC | 40 | UUAU | 20 | GCAG | 28 | UGAA | 5 | UAGA | 21 |

| | | | | | | | | | | | | |
|---|---|---|---|---|---|---|---|---|---|---|---|---|
| 19 | AGCA | 136 | ACGG | 26 | AGAA | 16 | UUUA | 26 | UAGU | 5 | UUUG | 21 |
| 20 | UCCG | 130 | AAAA | 25 | UAAA | 15 | UAAU | 22 | CCUU | 5 | ACCU | 21 |
| 21 | UUUG | 120 | CAUG | 24 | ACCA | 15 | UAUA | 22 | CAAU | 5 | UCCG | 20 |
| 22 | CUCG | 92 | UAUA | 24 | AUAA | 13 | AUAA | 22 | GUAA | 4 | UAAU | 17 |
| 23 | UAAG | 91 | ACUU | 24 | AUAU | 13 | UAAA | 21 | GCGA | 4 | CGAA | 17 |
| 24 | AAAC | 69 | AUGA | 19 | UCCG | 13 | GAUA | 21 | AAUA | 4 | CUCA | 17 |
| 25 | GGGA | 67 | ACCU | 19 | AAAC | 12 | AAAU | 20 | UUCA | 4 | UUUU | 16 |
| 26 | CACG | 67 | AAGU | 18 | UAUU | 12 | AUUU | 20 | CUUG | 4 | GAAG | 16 |
| 27 | CUCA | 64 | CAAG | 17 | AUUU | 12 | GACA | 19 | UGCG | 4 | ACAG | 16 |
| 28 | CGCC | 64 | GUCA | 17 | UUGA | 12 | AACU | 17 | AUCA | 4 | CGCA | 16 |
| 29 | UUAC | 57 | AUAC | 17 | AUCU | 12 | UUAA | 16 | AAGU | 4 | UUAA | 15 |
| 30 | GCUA | 52 | UAAC | 16 | UAAU | 11 | AAAG | 16 | UGUG | 4 | GAUA | 15 |
| 31 | AACA | 50 | AAAC | 16 | UAAC | 11 | UAUU | 15 | GUGA | 3 | AAAA | 15 |
| 32 | AACC | 49 | UAAA | 16 | GGGA | 11 | AAUA | 15 | GCAA | 3 | AUCC | 14 |
| 33 | AUUU | 48 | AGUA | 16 | GAAG | 11 | CUUU | 15 | UUUU | 3 | UCAC | 13 |
| 34 | UCAC | 48 | AUUU | 15 | CUGG | 11 | AACC | 15 | GAUA | 3 | UCAG | 13 |
| 35 | GACA | 47 | GUUU | 15 | AAUU | 11 | UUAG | 14 | AUUU | 3 | CUCG | 13 |
| 36 | AAAA | 45 | AAUU | 15 | UUGG | 10 | GUCA | 14 | UCUU | 3 | UUAC | 12 |
| 37 | GUCA | 44 | GUUA | 15 | UUGU | 10 | GAAU | 14 | UGUU | 3 | CAGU | 12 |
| 38 | UUAU | 42 | AGCA | 14 | ACGU | 10 | CAAA | 13 | ACAA | 3 | UAAA | 11 |
| 39 | UAAA | 41 | AAUC | 14 | UACG | 9 | UUUG | 12 | UCUC | 3 | ACUU | 11 |
| 40 | CUAG | 41 | GUGG | 14 | UCGG | 9 | AUAU | 12 | UGCU | 3 | UCAU | 10 |
| 41 | ACCC | 40 | UCCG | 13 | UAGG | 8 | UUCU | 12 | UAGA | 3 | CUAG | 10 |
| 42 | AAUA | 39 | UUCU | 13 | UUCA | 8 | CAUC | 12 | UUGG | 3 | CUAA | 10 |

| | | | | | | | | | | | | |
|---|---|---|---|---|---|---|---|---|---|---|---|---|
| 43 | GUUU | 39 | ACAU | 13 | AGUA | 8 | AAAC | 11 | AAGC | 3 | UACG | 9 |
| 44 | AAUU | 38 | GGAG | 13 | CCUC | 8 | GAGG | 11 | UAAC | 2 | UUUA | 9 |
| 45 | UUCU | 35 | AUAA | 13 | GGAG | 8 | AAUU | 11 | UAUA | 2 | CUUG | 9 |
| 46 | ACAU | 35 | UUGA | 13 | GUAG | 8 | GACC | 10 | AUAA | 2 | AUAG | 9 |
| 47 | UGCG | 35 | CCUG | 13 | GUAU | 8 | UCAU | 9 | CUUU | 2 | GGGU | 9 |
| 48 | CACC | 35 | CUCG | 12 | GGUU | 8 | UUCA | 9 | AACC | 2 | UUCC | 9 |
| 49 | UGGU | 34 | GUCU | 12 | UAGU | 7 | UCUU | 9 | UUUG | 2 | UCCC | 9 |
| 50 | CUUG | 33 | CGUG | 12 | AAUA | 7 | AUUA | 9 | AUAU | 2 | UUCA | 8 |
| 51 | UGAC | 32 | UAAG | 11 | UCUU | 7 | GAAC | 9 | AUUA | 2 | CAAC | 8 |
| 52 | ACGU | 30 | CAAC | 11 | UUUG | 7 | AAUG | 9 | GAAC | 2 | UGCC | 8 |
| 53 | GUUA | 30 | ACAA | 11 | AACC | 7 | UACC | 9 | AAUG | 2 | CGUA | 8 |
| 54 | CAAC | 30 | GAGU | 11 | UUCU | 7 | GGAG | 9 | GGGA | 2 | CGGG | 8 |
| 55 | UUUC | 30 | AUUA | 9 | GGCA | 7 | UUGU | 9 | UCAC | 2 | UUAU | 8 |
| 56 | UUUA | 28 | GAAU | 9 | AAAU | 6 | UGAA | 8 | CAAC | 2 | GAAU | 8 |
| 57 | GCAG | 28 | UAUU | 9 | UGAU | 6 | UGUU | 8 | GUUU | 2 | UACC | 8 |
| 58 | UUAA | 27 | AUAU | 9 | GAGU | 6 | GGGA | 8 | GUGG | 2 | GCCU | 8 |
| 59 | UCAA | 27 | UUAU | 8 | UGAA | 6 | UCAC | 8 | UUAC | 2 | GAGU | 7 |
| 60 | GUAG | 26 | AAAU | 8 | UUAC | 6 | CAAC | 8 | UAAG | 2 | CCUU | 7 |
| 61 | GGAG | 25 | CCAA | 8 | GAAC | 6 | AGCU | 8 | AGAA | 2 | GGAG | 7 |
| 62 | GAUA | 25 | GUUG | 8 | GUUA | 6 | CUCG | 8 | CCAA | 2 | CCCA | 7 |
| 63 | UGAG | 25 | UUAG | 7 | GUCA | 6 | UUCC | 8 | ACUA | 2 | GAUG | 7 |
| 64 | UUGC | 25 | UUUG | 7 | AUCC | 6 | GUAG | 8 | GUAC | 2 | AAAC | 6 |
| 65 | CGGU | 25 | CGCC | 7 | AUGA | 6 | GUAU | 8 | UGUA | 2 | AAUA | 6 |
| 66 | GUAU | 24 | AAUA | 7 | AGUU | 5 | UACG | 7 | UUGA | 2 | UGUG | 6 |

| | | | | | | | | | | | | |
|---|---|---|---|---|---|---|---|---|---|---|---|---|
| 67 | UCAU | 23 | GCAG | 7 | GAUA | 5 | UAGU | 7 | UGGU | 2 | UAUA | 6 |
| 68 | UUGU | 23 | UUAA | 7 | UAUA | 5 | CUUG | 7 | UGCA | 2 | UGGC | 6 |
| 69 | AAAG | 23 | UAAU | 7 | CUUU | 5 | GUUU | 7 | ACGA | 2 | CAAG | 6 |
| 70 | UAUG | 22 | AACU | 7 | AUUA | 5 | GUGG | 7 | CCAU | 2 | CACG | 6 |
| 71 | UCUU | 21 | AUUC | 7 | GUUU | 5 | ACUU | 7 | AGUG | 2 | CCUA | 6 |
| 72 | GGCA | 21 | AAGA | 7 | UGUA | 5 | GCUA | 7 | ACUG | 2 | CCCC | 6 |
| 73 | AUCC | 21 | UGCG | 6 | UUAG | 5 | AUCU | 7 | AAGG | 2 | UAGC | 6 |
| 74 | AUUA | 21 | GUAG | 6 | AUAC | 5 | UACA | 7 | UGAC | 2 | UGGG | 6 |
| 75 | CAAA | 21 | UUGC | 6 | CAGU | 5 | AUAC | 7 | GCGU | 2 | AUGG | 6 |
| 76 | ACAA | 21 | UCUU | 6 | GAAU | 5 | CUAA | 7 | CGGC | 2 | UAGU | 5 |
| 77 | AGCU | 21 | CCCG | 6 | AAGA | 5 | UCCA | 7 | GAGA | 1 | UGCG | 5 |
| 78 | UCAG | 21 | UCCC | 6 | GACG | 5 | CUAC | 7 | GAAG | 1 | AUAA | 5 |
| 79 | AAAU | 20 | AGAU | 6 | GCGG | 5 | GAGU | 6 | AAAA | 1 | UAAG | 5 |
| 80 | AUAA | 19 | GAUC | 6 | AAAG | 4 | CCUU | 6 | UUUC | 1 | AAGG | 5 |
| 81 | GAAU | 19 | GACG | 6 | CCUU | 4 | CAAU | 6 | GACA | 1 | UUUC | 5 |
| 82 | AUGA | 18 | GAUU | 6 | AAGU | 4 | UGCG | 6 | UUAG | 1 | GACA | 5 |
| 83 | CGCA | 18 | CCGC | 6 | UCAC | 4 | AUCA | 6 | GUCA | 1 | UUCU | 5 |
| 84 | GCCC | 18 | UACG | 5 | UAAG | 4 | ACAA | 6 | UUCU | 1 | GCUA | 5 |
| 85 | AUCG | 17 | GCCA | 5 | ACUU | 4 | UCUC | 6 | AGCU | 1 | GGCA | 5 |
| 86 | AUGU | 17 | UCAC | 5 | UACA | 4 | UUAC | 6 | ACUU | 1 | GUUA | 5 |
| 87 | AGGA | 17 | CUAG | 5 | GGUC | 4 | UAAG | 6 | GCUA | 1 | UAUC | 5 |
| 88 | UAUU | 16 | GGUA | 5 | GGUA | 4 | AGAA | 6 | AUCU | 1 | UCUG | 5 |
| 89 | GGAU | 16 | AAGC | 5 | CAAA | 4 | GGCA | 6 | UACA | 1 | CCAG | 5 |
| 90 | UGUG | 16 | CCCA | 5 | ACAU | 4 | UCAA | 6 | AUAC | 1 | UUGU | 5 |

| | | | | | | | | | | | | |
|---|---|---|---|---|---|---|---|---|---|---|---|---|
| 91 | UAAU | 15 | CUAU | 5 | ACCG | 4 | AAGA | 6 | GGCA | 1 | GUAG | 5 |
| 92 | UUCA | 15 | AGAC | 5 | GUUC | 4 | CUCU | 6 | UCAA | 1 | CUCC | 5 |
| 93 | UGAA | 15 | CAUU | 5 | AUCA | 3 | UGAU | 5 | GUUA | 1 | GUCU | 5 |
| 94 | AAGU | 15 | ACUC | 5 | UGUU | 3 | CCAA | 5 | CUGA | 1 | CAGA | 5 |
| 95 | GGUA | 15 | CAGG | 5 | ACAA | 3 | GUUA | 5 | UCAG | 1 | AUCA | 4 |
| 96 | AACU | 14 | AACC | 4 | UGCU | 3 | CUGA | 5 | CUAG | 1 | UGUU | 4 |
| 97 | AAUC | 14 | UGGU | 4 | CCAA | 3 | UCCU | 5 | UAUG | 1 | UUGG | 4 |
| 98 | CCCG | 14 | UUUC | 4 | AAUG | 3 | UCCG | 5 | AUAG | 1 | CUUU | 4 |
| 99 | UGCC | 14 | GAUA | 4 | ACGA | 3 | CCCC | 5 | UGGC | 1 | UGGU | 4 |
| 100 | CUGC | 14 | AUCC | 4 | CCUA | 3 | GUGU | 5 | UAUC | 1 | UGCA | 4 |
| 101 | UUGA | 13 | AGGA | 4 | UCUG | 3 | CACA | 5 | CUGU | 1 | GUCA | 4 |
| 102 | UCUG | 13 | GGAU | 4 | CUUC | 3 | GAUC | 5 | UCGG | 1 | AUCU | 4 |
| 103 | CUGA | 13 | UGCC | 4 | AUUG | 3 | AAGU | 4 | GUUG | 1 | CUGU | 4 |
| 104 | CAUG | 13 | CUGC | 4 | CUGA | 3 | UGCU | 4 | AGUA | 1 | UCGG | 4 |
| 105 | AAGC | 13 | GCUU | 4 | UCCU | 3 | ACUA | 4 | CCUC | 1 | CCUG | 4 |
| 106 | UCCC | 13 | AUGG | 4 | CUCA | 3 | GUAC | 4 | AGCC | 1 | CGUG | 4 |
| 107 | UGGC | 13 | GAGG | 4 | CUCG | 3 | UGUA | 4 | CACC | 1 | AAGA | 4 |
| 108 | AGAA | 12 | GUAC | 4 | UCUA | 3 | UCAG | 4 | CAAG | 1 | GUGU | 4 |
| 109 | GAAC | 12 | GCCG | 4 | AGUC | 3 | CUAG | 4 | CACG | 1 | AUGA | 4 |
| 110 | CAGU | 12 | CCGA | 4 | AACU | 3 | UAUG | 4 | UCUG | 1 | CUGG | 4 |
| 111 | UGUU | 12 | GUGC | 4 | UCCA | 3 | GCAU | 4 | CCAG | 1 | ACCC | 4 |
| 112 | CCAU | 12 | CUAC | 4 | CUCU | 3 | CAGU | 4 | CUUC | 1 | AGCA | 4 |
| 113 | GUCC | 12 | GCUA | 3 | GGCU | 3 | CUCC | 4 | AUUG | 1 | UGAU | 3 |
| 114 | UCGC | 12 | GACA | 3 | GCUG | 3 | GACG | 4 | GGUA | 1 | UAUU | 3 |

| | | | | | | | | | | | | |
|---|---|---|---|---|---|---|---|---|---|---|---|---|
| 115 | GAGC | 12 | UCAA | 3 | AUUC | 3 | ACCA | 4 | CCCU | 1 | AUUA | 3 |
| 116 | ACCA | 11 | UGAG | 3 | ACAC | 3 | AACG | 4 | GCUC | 1 | GAAC | 3 |
| 117 | AUCU | 11 | UAUG | 3 | AGAU | 3 | GCAC | 4 | UCGA | 1 | GUUU | 3 |
| 118 | CCUC | 11 | CAAA | 3 | CAUG | 3 | GCCC | 4 | CUAU | 1 | AGAA | 3 |
| 119 | UGAU | 11 | AGCU | 3 | UGAG | 2 | AUUC | 4 | CAGG | 1 | UGAC | 3 |
| 120 | AUCA | 11 | GAAC | 3 | CAAU | 2 | ACAC | 4 | GGGU | 1 | GCGU | 3 |
| 121 | UCCU | 11 | GAGC | 3 | AAGC | 2 | UGAG | 3 | UGCC | 1 | UACA | 3 |
| 122 | CCAG | 11 | ACCA | 3 | CAAC | 2 | UGUG | 3 | CCUA | 1 | UCAA | 3 |
| 123 | AUAU | 10 | CCUC | 3 | AAGG | 2 | UAGA | 3 | CCUG | 1 | UAUG | 3 |
| 124 | GAGU | 10 | UCCU | 3 | GUGG | 2 | UUGA | 3 | CGUG | 1 | GUUG | 3 |
| 125 | AGUU | 10 | AGUU | 3 | ACUG | 2 | AUAG | 3 | GGUC | 1 | GCCA | 3 |
| 126 | AUAC | 10 | UUCC | 3 | GGGU | 2 | UGGC | 3 | CCGA | 1 | GAGG | 3 |
| 127 | ACAC | 10 | CUCC | 3 | CAAG | 2 | UAUC | 3 | CUUA | 1 | GUAU | 3 |
| 128 | GUGU | 10 | CUAA | 3 | CACG | 2 | CUGU | 3 | AGGC | 1 | CACA | 3 |
| 129 | GACC | 10 | GCCU | 3 | UUUC | 2 | UCGG | 3 | CGUA | 1 | GACG | 3 |
| 130 | ACGA | 9 | ACGC | 3 | GACA | 2 | GUUG | 3 | CGGG | 1 | ACCA | 3 |
| 131 | UCUA | 9 | AGUG | 3 | GCUA | 2 | AGUA | 3 | ACGC | 1 | AACG | 3 |
| 132 | AUUC | 9 | CGAG | 3 | UAUC | 2 | CCUC | 3 | CGAC | 1 | UCUA | 3 |
| 133 | AGAU | 9 | CCUA | 3 | CGUG | 2 | AGCC | 3 | CGCG | 1 | CUGC | 3 |
| 134 | CUUA | 9 | GGGG | 3 | UAUG | 2 | CACC | 3 | GGGC | 1 | GUCC | 3 |
| 135 | GCUU | 9 | CAAU | 3 | GUUG | 2 | ACAG | 3 | AGAG | 1 | UCGC | 3 |
| 136 | CUGU | 9 | ACUG | 3 | AGCU | 2 | UCCC | 3 | GCGC | 1 | UGGA | 3 |
| 137 | GCAC | 9 | GGCG | 3 | AGAG | 2 | CCCA | 3 | AGGA | 1 | AAAU | 2 |
| 138 | GUCG | 9 | CCCC | 2 | UCGA | 2 | GAUG | 3 | GGGG | 1 | AGUU | 2 |

| | | | | | | | | | | | | |
|---|---|---|---|---|---|---|---|---|---|---|---|---|
| 139 | GAUC | 9 | ACCC | 2 | CUUA | 2 | GUCU | 3 | CGAU | 1 | CAAU | 2 |
| 140 | CUUU | 8 | UGAC | 2 | GCAU | 2 | AUGA | 3 | CCGG | 1 | UCUU | 2 |
| 141 | UACA | 8 | UCAU | 2 | ACCU | 2 | CUGG | 3 | UUCG | 0 | AACC | 2 |
| 142 | CCAA | 8 | GGCA | 2 | CGCA | 2 | UCUA | 3 | GCCA | 0 | CCAA | 2 |
| 143 | AUUG | 8 | AUCG | 2 | UUCC | 2 | ACUC | 3 | GAUU | 0 | ACUA | 2 |
| 144 | UCCA | 8 | AUGU | 2 | GAUG | 2 | AAUC | 3 | AACA | 0 | CCAU | 2 |
| 145 | UUCC | 8 | UGUG | 2 | UAGC | 2 | CCCG | 3 | UUAU | 0 | AGCU | 2 |
| 146 | CUCC | 8 | CAGU | 2 | CUCC | 2 | GUGC | 3 | GCAG | 0 | AGUA | 2 |
| 147 | CUAA | 8 | CCAU | 2 | GCCA | 2 | GUCG | 3 | AACU | 0 | CUUC | 2 |
| 148 | GCCU | 8 | UGAU | 2 | ACUC | 2 | CAUA | 3 | GAAU | 0 | AUUG | 2 |
| 149 | ACGC | 8 | AUCA | 2 | AUCG | 2 | AGGU | 3 | CAAA | 0 | GGUC | 2 |
| 150 | CCCA | 8 | ACAC | 2 | AUGU | 2 | CGUU | 3 | CAUC | 0 | ACGC | 2 |
| 151 | CCAC | 8 | GCAC | 2 | UCGU | 2 | AGUU | 2 | GAGG | 0 | CGAC | 2 |
| 152 | UAUA | 7 | GUCG | 2 | UUGC | 2 | UUGG | 2 | AAUU | 0 | CGCG | 2 |
| 153 | AAUG | 7 | CCAC | 2 | GGAU | 2 | UGGU | 2 | GACC | 0 | GGGC | 2 |
| 154 | CUUC | 7 | AAUG | 2 | GAUU | 2 | UGCA | 2 | UACC | 0 | AGAG | 2 |
| 155 | AGUC | 7 | CAGA | 2 | GGUG | 2 | ACGA | 2 | GGAG | 0 | AACA | 2 |
| 156 | CUAU | 7 | AUAG | 2 | AGAC | 2 | CAAG | 2 | UUGU | 0 | GACC | 2 |
| 157 | CAGA | 7 | CCCU | 2 | GCUU | 2 | CACG | 2 | CUCG | 0 | GCAC | 2 |
| 158 | AGGU | 7 | AACG | 2 | UACU | 2 | UCUG | 2 | UUCC | 0 | GCCC | 2 |
| 159 | UCUC | 7 | CUCU | 2 | AAUC | 2 | CCAG | 2 | GUAG | 0 | ACUC | 2 |
| 160 | ACUA | 7 | GAUG | 2 | CCCG | 2 | CUUC | 2 | GUAU | 0 | GAGC | 2 |
| 161 | AUAG | 7 | UACU | 2 | AUGC | 2 | AUUG | 2 | CUAA | 0 | AUCG | 2 |
| 162 | CCCU | 7 | CGAC | 2 | CGAG | 2 | GGUA | 2 | UCCA | 0 | AUGU | 2 |

| | | | | | | | | | | | | |
|---|---|---|---|---|---|---|---|---|---|---|---|---|
| 163 | UACC | 7 | CGAA | 2 | CGCU | 2 | CCCU | 2 | CUAC | 0 | GCGG | 2 |
| 164 | CACA | 7 | CAUA | 2 | UGUG | 1 | GCUC | 2 | AAGA | 0 | UCGU | 2 |
| 165 | AACG | 7 | GCUG | 2 | UGCG | 1 | UCGA | 2 | CUCU | 0 | AGUC | 2 |
| 166 | CGGA | 7 | CGCG | 2 | UGGU | 1 | CUAU | 2 | UCCU | 0 | UUGC | 2 |
| 167 | AGUA | 6 | UGCU | 2 | UGCA | 1 | CGAA | 2 | UCCG | 0 | GGAU | 2 |
| 168 | UAGU | 6 | UAUC | 2 | GCGU | 1 | CUCA | 2 | CCCC | 0 | ACGU | 2 |
| 169 | ACUU | 6 | UUGG | 2 | CCAU | 1 | CGCA | 2 | GUGU | 0 | CCGU | 2 |
| 170 | CUCU | 6 | GCGG | 2 | GUAC | 1 | UAGC | 2 | CACA | 0 | AAAG | 1 |
| 171 | UCGA | 6 | UGGG | 2 | UCAG | 1 | ACCC | 2 | GAUC | 0 | UGAA | 1 |
| 172 | AGAC | 6 | GUUC | 2 | CUAG | 1 | CUGC | 2 | GCAU | 0 | AAGU | 1 |
| 173 | AUGC | 6 | CACG | 1 | CGGG | 1 | GUCC | 2 | CAGU | 0 | ACAA | 1 |
| 174 | GCGU | 6 | CUCA | 1 | CCAG | 1 | GAGC | 2 | CUCC | 0 | AAGC | 1 |
| 175 | ACAG | 6 | UUAC | 1 | CUGU | 1 | AUCG | 2 | GACG | 0 | AUAU | 1 |
| 176 | CCGU | 6 | AACA | 1 | UCAA | 1 | ACGG | 2 | ACCA | 0 | GUAC | 1 |
| 177 | CAGC | 6 | CACC | 1 | AGCC | 1 | GGCU | 2 | AACG | 0 | UGUA | 1 |
| 178 | CACU | 6 | GUAU | 1 | GCUC | 1 | GGUG | 2 | GCAC | 0 | UUGA | 1 |
| 179 | AGUG | 6 | AAAG | 1 | CCGA | 1 | AGAU | 2 | GCCC | 0 | AUAC | 1 |
| 180 | AUGG | 6 | CGCA | 1 | CACC | 1 | CGUC | 2 | AUUC | 0 | CCUC | 1 |
| 181 | CAUU | 6 | UUCA | 1 | CUAU | 1 | CCAU | 1 | ACAC | 0 | AGCC | 1 |
| 182 | CGUU | 6 | UCUG | 1 | AGGA | 1 | AGUG | 1 | ACAG | 0 | GGUA | 1 |
| 183 | GAGG | 5 | CUGA | 1 | CGAU | 1 | ACUG | 1 | UCCC | 0 | CCCU | 1 |
| 184 | AAGA | 5 | UGGC | 1 | CCGG | 1 | CAGG | 1 | CCCA | 0 | GCUC | 1 |
| 185 | GACG | 5 | UGUU | 1 | ACAG | 1 | GGGU | 1 | GAUG | 0 | UCGA | 1 |
| 186 | GGCU | 5 | GUCC | 1 | CUAA | 1 | UGCC | 1 | GUCU | 0 | CCGA | 1 |

| | | | | | | | | | | | | |
|---|---|---|---|---|---|---|---|---|---|---|---|---|
| 187 | GUGG | 5 | UCGC | 1 | UCCC | 1 | CCUA | 1 | AUGA | 0 | CUUA | 1 |
| 188 | GGGU | 5 | AUCU | 1 | GCCU | 1 | CCUG | 1 | CUGG | 0 | GCGC | 1 |
| 189 | GAUG | 5 | CCAG | 1 | GUCU | 1 | CGUG | 1 | UCUA | 0 | GAUU | 1 |
| 190 | UACU | 5 | UCUA | 1 | CAGA | 1 | GGUC | 1 | ACUC | 0 | AACU | 1 |
| 191 | CGAG | 5 | CUUA | 1 | GUGU | 1 | CCGA | 1 | AAUC | 0 | CAAA | 1 |
| 192 | GCUC | 5 | CUGU | 1 | ACCC | 1 | CUUA | 1 | CCCG | 0 | CAUC | 1 |
| 193 | GUCU | 5 | CUUU | 1 | GUCC | 1 | AGGC | 1 | GUGC | 0 | AAUU | 1 |
| 194 | UGUC | 5 | UACA | 1 | GCAC | 1 | ACCU | 1 | GUCG | 0 | UCCA | 1 |
| 195 | UAGA | 5 | UCCA | 1 | CCGU | 1 | AUCC | 1 | CAUA | 0 | CUCU | 1 |
| 196 | CCUG | 5 | AGGU | 1 | GCCG | 1 | UGGG | 1 | AGGU | 0 | ACGG | 1 |
| 197 | CGAC | 5 | UCUC | 1 | CAGC | 1 | CAGA | 1 | CGUU | 0 | GGCU | 1 |
| 198 | CGAA | 5 | UACC | 1 | GUGC | 1 | AGCA | 1 | CGAA | 0 | GGUG | 1 |
| 199 | AGCG | 5 | UAGU | 1 | GUCG | 1 | UCGC | 1 | CUCA | 0 | ACAU | 1 |
| 200 | CAUA | 5 | AUGC | 1 | AGGU | 1 | AUGU | 1 | CGCA | 0 | AGAC | 1 |
| 201 | CCUA | 4 | ACAG | 1 | UGUC | 1 | GCGG | 1 | UAGC | 0 | GCUU | 1 |
| 202 | GCUG | 4 | CAGC | 1 | CACU | 1 | UCGU | 1 | ACCC | 0 | GCCG | 1 |
| 203 | CGUG | 4 | CGUU | 1 | UAGA | 0 | AGUC | 1 | CUGC | 0 | GCUG | 1 |
| 204 | UAGC | 4 | GGCU | 1 | UCUC | 0 | ACAU | 1 | GUCC | 0 | CAGC | 1 |
| 205 | CGCU | 4 | GCUC | 1 | UGAC | 0 | AGAC | 1 | GAGC | 0 | GGAC | 1 |
| 206 | UGCA | 4 | UGUC | 1 | ACUA | 0 | GCUU | 1 | AUCG | 0 | GACU | 1 |
| 207 | CGUA | 4 | UAGA | 1 | AGUG | 0 | GCCG | 1 | ACGG | 0 | CAUU | 1 |
| 208 | CGCG | 4 | AGCG | 1 | CGGC | 0 | GCUG | 1 | GGCU | 0 | UACU | 1 |
| 209 | GGGC | 4 | UAGC | 1 | CAGG | 0 | CAGC | 1 | GGUG | 0 | AGCG | 1 |
| 210 | AGGC | 4 | CGCU | 1 | AUAG | 0 | GGAC | 1 | AGAU | 0 | GGCC | 1 |

| | | | | | | | | | | | | | |
|---|---|---|---|---|---|---|---|---|---|---|---|---|---|
| 211 | GGGG | 4 | UGCA | 1 | UGCC | 0 | GACU | 1 | CGUC | 0 | GGUU | 1 |
| 212 | UGGA | 4 | UGGA | 1 | CGUA | 0 | CCGC | 1 | ACCU | 0 | CGGA | 1 |
| 213 | UCGG | 3 | UGUA | 1 | UGGC | 0 | AUGC | 1 | AUCC | 0 | AUUU | 0 |
| 214 | UAGG | 3 | CGAU | 1 | CCUG | 0 | UGUC | 1 | UGGG | 0 | UCUC | 0 |
| 215 | UGUA | 3 | CGGC | 1 | ACGC | 0 | ACCG | 1 | CAGA | 0 | UGCU | 0 |
| 216 | UGCU | 3 | GCGC | 1 | CGAC | 0 | UAGG | 0 | AGCA | 0 | AAUG | 0 |
| 217 | CAAU | 3 | GGAC | 1 | CGCG | 0 | AAGC | 0 | UCGC | 0 | GUGG | 0 |
| 218 | AAGG | 3 | GGCC | 1 | GGGC | 0 | AAGG | 0 | AUGU | 0 | ACGA | 0 |
| 219 | ACUG | 3 | UCGU | 1 | CCCU | 0 | UGAC | 0 | GCGG | 0 | AGUG | 0 |
| 220 | UAUC | 3 | GACU | 1 | GCGC | 0 | GCGU | 0 | UCGU | 0 | ACUG | 0 |
| 221 | AGAG | 3 | CGUC | 1 | AGGC | 0 | CGGC | 0 | AGUC | 0 | CGGC | 0 |
| 222 | ACCU | 3 | CCUU | 1 | GGGG | 0 | CGUA | 0 | ACAU | 0 | CUGA | 0 |
| 223 | GAUU | 3 | CCGG | 1 | GCAG | 0 | CGGG | 0 | AGAC | 0 | CACC | 0 |
| 224 | GUAC | 3 | CAUC | 1 | CGAA | 0 | ACGC | 0 | GCUU | 0 | CUAU | 0 |
| 225 | CGAU | 3 | GGUU | 1 | UACC | 0 | CGAC | 0 | GCCG | 0 | AGGC | 0 |
| 226 | GCCG | 3 | GGUG | 1 | CCCA | 0 | CGCG | 0 | GCUG | 0 | AGGA | 0 |
| 227 | CGGC | 3 | AGCC | 0 | CCCC | 0 | GGGC | 0 | CAGC | 0 | GGGG | 0 |
| 228 | GCGC | 3 | ACGU | 0 | UGGG | 0 | AGAG | 0 | GGAC | 0 | CGAU | 0 |
| 229 | GGAC | 3 | CGGU | 0 | AUGG | 0 | GCGC | 0 | GACU | 0 | CCGG | 0 |
| 230 | GGCC | 3 | UUGU | 0 | CACA | 0 | AGGA | 0 | CCGC | 0 | CUAC | 0 |
| 231 | CCGC | 3 | UCAG | 0 | AACG | 0 | GGGG | 0 | AUGC | 0 | GAUC | 0 |
| 232 | UUGG | 2 | GCCC | 0 | CUGC | 0 | CGAU | 0 | UGUC | 0 | AUUC | 0 |
| 233 | GCGG | 2 | GUGU | 0 | UCGC | 0 | CCGG | 0 | ACCG | 0 | ACAC | 0 |
| 234 | GUUG | 2 | GACC | 0 | UGGA | 0 | GCCU | 0 | GCCU | 0 | AAUC | 0 |

| | | | | | | | | | | | | |
|---|---|---|---|---|---|---|---|---|---|---|---|---|
| 235 | UCGU | 2 | AUUG | 0 | GACC | 0 | AUGG | 0 | AUGG | 0 | CCCG | 0 |
| 236 | CGGG | 2 | AGUC | 0 | GCCC | 0 | UGGA | 0 | UGGA | 0 | GUGC | 0 |
| 237 | CCGA | 2 | ACUA | 0 | GAGC | 0 | UUGC | 0 | UUGC | 0 | GUCG | 0 |
| 238 | UGGG | 2 | CACA | 0 | CAUC | 0 | GGAU | 0 | GGAU | 0 | CAUA | 0 |
| 239 | GACU | 2 | CGGA | 0 | ACGG | 0 | ACGU | 0 | ACGU | 0 | AGGU | 0 |
| 240 | CGUC | 2 | UCGA | 0 | GGAC | 0 | CCGU | 0 | CCGU | 0 | CGUU | 0 |
| 241 | AGGG | 2 | GCGU | 0 | GACU | 0 | CAUU | 0 | CAUU | 0 | AGAU | 0 |
| 242 | CUGG | 1 | CCGU | 0 | CAUU | 0 | UACU | 0 | UACU | 0 | CGUC | 0 |
| 243 | CCUU | 1 | CACU | 0 | AGCG | 0 | AGCG | 0 | AGCG | 0 | CCGC | 0 |
| 244 | GGUC | 1 | GGGU | 0 | GGCC | 0 | GGCC | 0 | GGCC | 0 | AUGC | 0 |
| 245 | ACCG | 1 | CGUA | 0 | CGGA | 0 | GGUU | 0 | GGUU | 0 | UGUC | 0 |
| 246 | ACUC | 1 | GGGC | 0 | CUAC | 0 | CGGA | 0 | CGGA | 0 | ACCG | 0 |
| 247 | CCGG | 1 | AGGC | 0 | GAUC | 0 | CAUG | 0 | CAUG | 0 | CAUG | 0 |
| 248 | GUGC | 1 | UCGG | 0 | CAUA | 0 | CGCC | 0 | CGCC | 0 | CGCC | 0 |
| 249 | CAUC | 1 | UAGG | 0 | CGUU | 0 | CGAG | 0 | CGAG | 0 | CGAG | 0 |
| 250 | ACGG | 1 | AAGG | 0 | CGUC | 0 | GGCG | 0 | GGCG | 0 | GGCG | 0 |
| 251 | GGCG | 1 | AGAG | 0 | CCGC | 0 | CCAC | 0 | CCAC | 0 | CCAC | 0 |
| 252 | GGUU | 0 | CGGG | 0 | CGCC | 0 | GUUC | 0 | GUUC | 0 | GUUC | 0 |
| 253 | GUUC | 0 | AGGG | 0 | GGCG | 0 | CGCU | 0 | CGCU | 0 | CGCU | 0 |
| 254 | GGUG | 0 | CUGG | 0 | CCAC | 0 | CGGU | 0 | CGGU | 0 | CGGU | 0 |
| 255 | CAGG | 0 | GGUC | 0 | CGGU | 0 | CACU | 0 | CACU | 0 | CACU | 0 |
| 256 | CUAC | 0 | ACCG | 0 | AGGG | 0 | AGGG | 0 | AGGG | 0 | AGGG | 0 |
| uncovered sequence # | 5 | | 30 | | 54 | | 41 | | 116 | | 44 | |

## Appendix S1. Calculation of the Spearman's rank correlation coefficient.

The original ranking of the 34 sequences tested, based on normalized NGS data, was re-designated as $\boldsymbol{d}_1$ and ranged from 1 to 34. A new ranking, referred to as $\boldsymbol{d}_2$, was established based on the observed yield of loop-closing ligation. The differences between the two rankings were calculated using the formula $\boldsymbol{d}_{\boldsymbol{i}} = \boldsymbol{d}_1 - \boldsymbol{d}_2$, where $\boldsymbol{i}$ ranges from 1 to 34. Consequently, the Spearman's rank correlation coefficient was calculated to be ρ = 0.84, as determined by the following equation and data:

$$\rho = 1 - \frac{6\sum d_i^2}{n(n^2 - 1)}$$

| NGS-rank original | reference rank, $\boldsymbol{d}_1$ | Observed yields of loop-closing ligation | Loop-closing rank, $\boldsymbol{d}_2$ | $\boldsymbol{d}_i$^2 = ($\boldsymbol{d}_1 - \boldsymbol{d}_2$)^2 |
|---|---|---|---|---|
| UGCG-1 | 1 | 60.7 | 3 | 0 |
| UUCG-3 | 2 | 63.0 | 1 | 0 |
| UUUG-4 | 3 | 43.3 | 6 | 9 |
| UACG-5 | 4 | 53.7 | 4 | 0 |
| UCCG-6 | 5 | 63.0 | 2 | 4 |
| UUAG-8 | 6 | 53.3 | 5 | 1 |
| UUGG-11 | 7 | 32.3 | 13 | 36 |
| UCGC-12 | 8 | 26.0 | 16 | 64 |
| UUGC-14 | 9 | 23.0 | 20 | 121 |
| UCGG-15 | 10 | 42.0 | 7 | 9 |
| CCUG-16 | 11 | 31.7 | 14 | 9 |
| CAGG-17 | 12 | 21.3 | 21 | 81 |
| UCUG-18 | 13 | 40.3 | 9 | 16 |
| CUCG-21 | 14 | 41.0 | 8 | 36 |
| GGGA-22 | 15 | 18.7 | 22 | 49 |
| CUGG-23 | 16 | 18.7 | 23 | 49 |
| CAAG-25 | 17 | 33.0 | 12 | 25 |
| UGGG-27 | 18 | 24.3 | 19 | 1 |
| GAGA-32 | 19 | 26.0 | 17 | 4 |
| GUGA-34 | 20 | 25.3 | 18 | 4 |
| CCAG-35 | 21 | 37.3 | 11 | 100 |
| UAAC-38 | 22 | 18.7 | 24 | 4 |
| UGGC-39 | 23 | 13.7 | 26 | 9 |
| GCGA-44 | 24 | 38.0 | 10 | 196 |

| | | | | |
|---|---|---|---|---|
| UUCA-69 | 25 | 9.0 | 27 | 4 |
| UCCA-75 | 26 | 7.3 | 28 | 4 |
| UUUA-86 | 27 | 16.0 | 25 | 4 |
| GAAA-87 | 28 | 31.3 | 15 | 169 |
| UGGU-93 | 29 | 3.0 | 29 | 0 |
| GCCC-240 | 30 | 0.4 | 30 | 0 |
| GACU-245 | 31 | 0.1 | 34 | 9 |
| AAAU-252 | 32 | 0.2 | 31 | 1 |
| AAGC-253 | 33 | 0.1 | 33 | 0 |
| AACC-256 | 34 | 60.7 | 32 | 4 |

**Appendix S2. Hypergeometric test.**

Results are represented in main text and Figure S13. The comparison of overlaps between the top 40 sequences from loop-closing ligation and the top 40 biological tetraloop sequences casupported by …n be effectively described using the hypergeometric distribution. For illustration, consider the following scenario: a bag contains 256 apples, 40 of which are green and 216 are red. If one were to randomly draw 40 apples from the bag without replacement, what would be the expected number of green apples? The probability of this outcome can be modelled by the following

$$p_X(k) = \Pr(X = k) = \frac{\binom{K}{k}\binom{N-K}{n-k}}{\binom{N}{n}},$$

equation:

where *N* (256 apples) represents the total population size, *K* is the number of successes (40 green apples) in the population, *n* is the number of draws (20, 30, 40, or 50 as used in this study), and *k* is the number of observed successes (green apples).

The hypergeometric test is employed to measure the statistical significance of over-representation or under-representation. An observed number of successes greater than expected indicates over-representation, while a number lower than expected suggests under-representation. The hypergeometric p-value is calculated as the probability of observing *k* or more successes in a test for over-representation (as shown Figure S13). Conversely, for under-representation, the hypergeometric p-value is calculated as the probability of observing *k* or fewer successes.

**Uncropped, full-size gel images, with no contrast changing, presented in the manuscript.**

Gel image for Figure 5D:

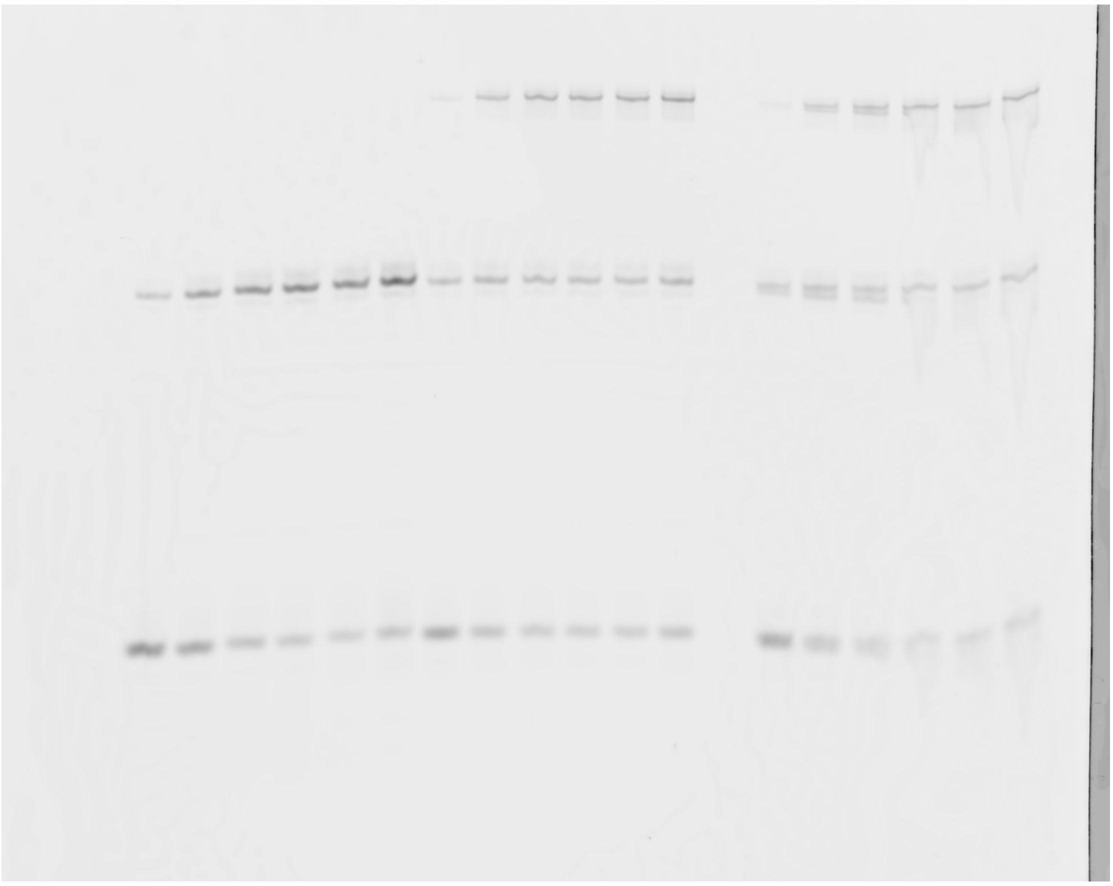

Gel image for Figure S2:

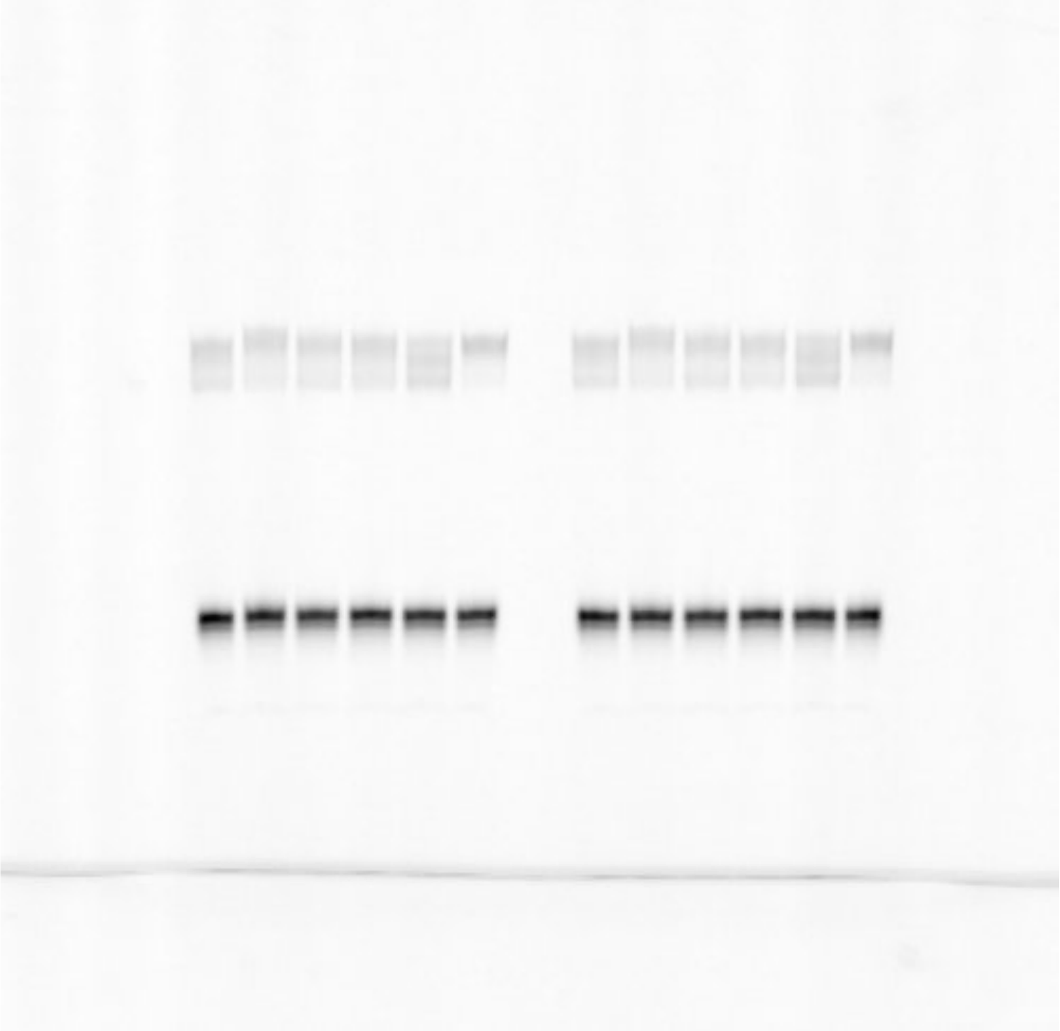

Gel image for Figure S3:

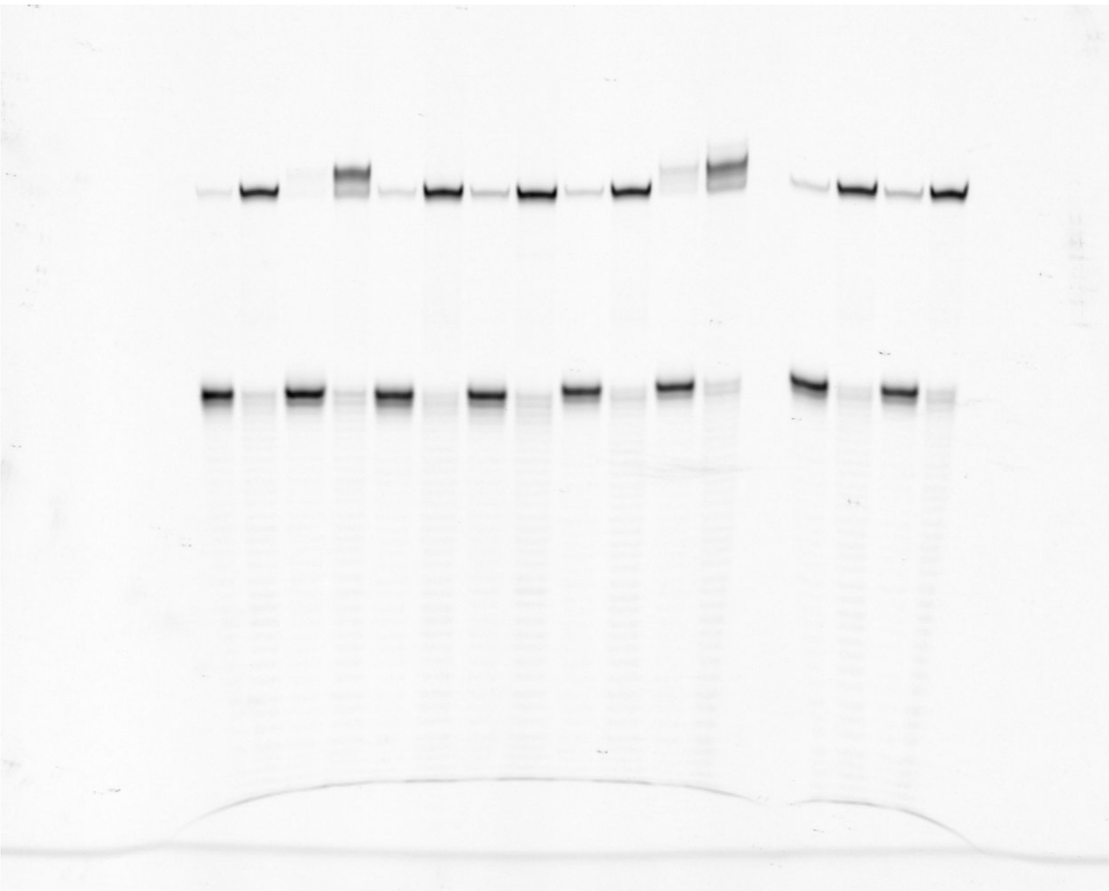

Gel image for Figure S5:

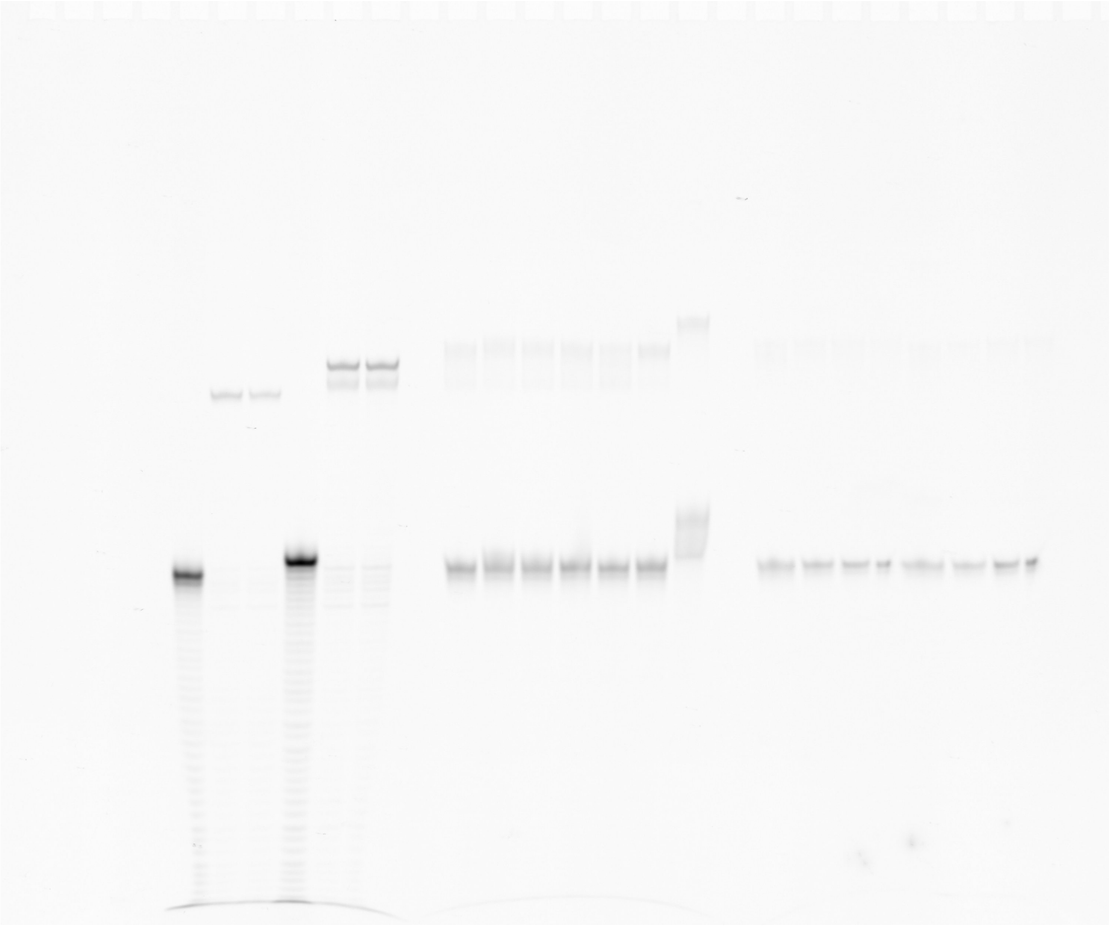

Gel image for Figure S10:

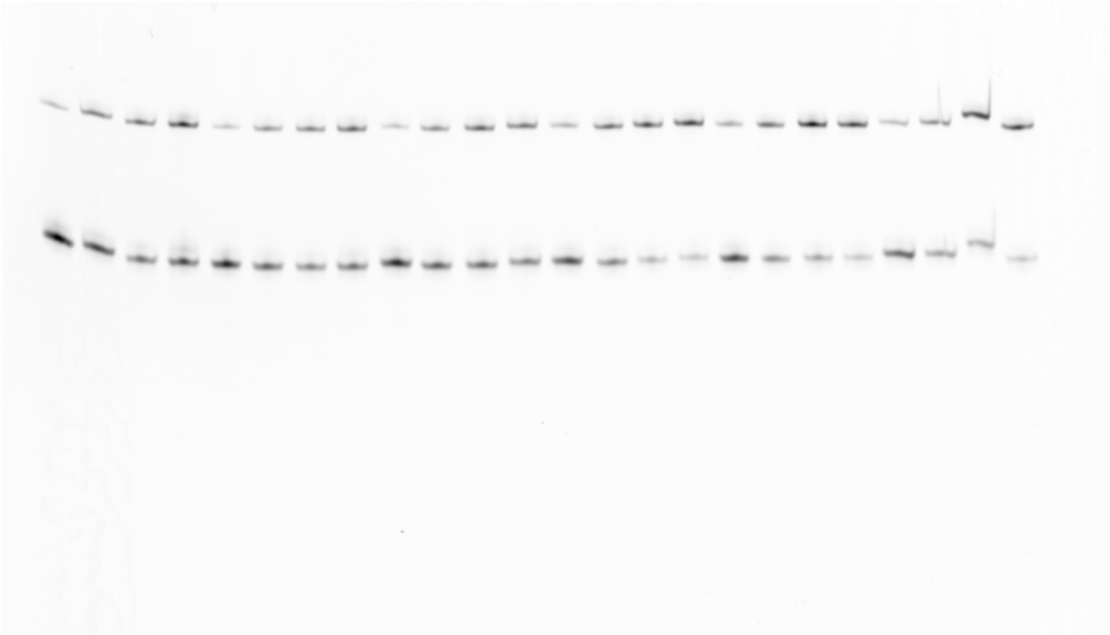

Supplement: Supplementary file 1 — Supporting Information [file ANIE-64-e202417370-s001.pdf]
